# Assortative mixing of opinions about COVID-19 vaccination in personal networks

**DOI:** 10.1038/s41598-024-53825-3

**Published:** 2024-02-09

**Authors:** Marian-Gabriel Hâncean, Jürgen Lerner, Matjaž Perc, José Luis Molina, Marius Geantă

**Affiliations:** 1 Department of Sociology, University of Bucharest, Panduri, 90-92, 050663 Bucharest, Romania; 2 The Research Institute of the University of Bucharest, University of Bucharest, Panduri, 90-92, 050663 Bucharest, Romania; 3 Department of Computer and Information Science, University of Konstanz, 78457 Konstanz, Germany; 4 Human Technology Center, RWTH Aachen University, 52062 Aachen, Germany; 5 Faculty of Natural Sciences and Mathematics, University of Maribor, Koroška Cesta 160, 2000 Maribor, Slovenia; 6 Department of Medical Research, China Medical University Hospital, China Medical University, Taichung, 404332 Taiwan; 7 Community Healthcare Center Dr. Adolf Drolc Maribor, Vošnjakova Ulica 2, 2000 Maribor, Slovenia; 8 Complexity Science Hub Vienna, Josefstädterstraße 39, 1080 Vienna, Austria; 9 Department of Physics, Kyung Hee University, 26 Kyungheedae-Ro, Dongdaemun-Gu, Seoul, Republic of Korea; 10 GRAFO - Department of Social and Cultural Anthtropology, Universitat Autònoma de Barcelona, 08193 Bellaterra (Cerdanyola del Vallès), Barcelona, Spain; 11 Center for Innovation in Medicine, Th. Pallady 42J, 032266 Bucharest, Romania

**Keywords:** Complex networks, Computational science

## Abstract

Many countries worldwide had difficulties reaching a sufficiently high vaccination uptake during the COVID-19 pandemic. Given this context, we collected data from a panel of 30,000 individuals, which were representative of the population of Romania (a country in Eastern Europe with a low 42.6% vaccination rate) to determine whether people are more likely to be connected to peers displaying similar opinions about COVID-19 vaccination. We extracted 443 personal networks, amounting to 4430 alters. We estimated multilevel logistic regression models with random-ego-level intercepts to predict individual opinions about COVID-19 vaccination. Our evidence indicates positive opinions about the COVID-19 vaccination cluster. Namely, the likelihood of having a positive opinion about COVID-19 vaccination increases when peers have, on average, a more positive attitude than the rest of the nodes in the network (OR 1.31, *p* < 0.001). We also found that individuals with higher education and age are more likely to hold a positive opinion about COVID-19 vaccination. With the given empirical data, our study cannot reveal whether this assortative mixing of opinions is due to social influence or social selection. However, it may nevertheless have implications for public health interventions, especially in countries that strive to reach higher uptake rates. Understanding opinions about vaccination can act as an early warning system for potential outbreaks, inform predictions about vaccination uptake, or help supply chain management for vaccine distribution.

## Introduction

Vaccination has been the paramount pharmaceutical intervention to halt the coronavirus disease pandemic (COVID-19)^1^. However, despite the actions taken in Europe by the European Commission to ensure timely access to vaccines for the Member States, various mass vaccination campaigns have not realized their potential, especially in Eastern European countries. Therefore, understanding the social mechanisms underpinning vaccination willingness is pivotal to fighting against both the still ongoing COVID-19 pandemic and future other pandemics.

Vaccination acceptance and its associated determinants are multiplex^2^. In the case of COVID-19, most of the literature has focused on individual-level predictors such as demographic characteristics (gender/sex, age, ethnicity/race, education, income, occupation), personal health history (medical conditions, personal experience with COVID-19), and beliefs (perceptions about the harms or efficiency of the vaccine). Significantly fewer studies have addressed supra-individual level factors such as healthcare and societal determinants^3^.

Scientists agree that vaccine acceptance is a complex decision-making process influenced by "experience, risk perception, culture, confidence in authorities and medicine"^4^. However, as we move from one study to another, many of the reported empirical findings are mixed or unclear (especially concerning race, age group, gender, employment status, and education)^4^. This inconsistency in the results may suggest that researchers have overlooked some predictors. Notably, the potential role of human networks in forming, reinforcing, or spreading opinions about COVID-19 vaccination has not been fully considered.

Disregarding network data is striking. Evidence shows that social networks affect health outcomes^5–7^ (e.g., the spread of obesity, COVID-19 infections, smoking, health screening, HPV vaccination uptake, happiness, depression, sleep, or loneliness). People do not live in isolation, and their behavior is not detached from the behavior of others. Health is a social network outcome. Individuals are interconnected, so their health is interconnected (health preferences, decisions, or habits). Research has already illustrated *assortativity* (connected individuals tend to share traits and behavior^8^) as an essential property of human networks^9^. For example, previous work has revealed the association between node characteristics (behavior) and network structure in the case of influenza vaccination^10^, local and global COVID-19 spreading^11,12^, sexually transmitted infections^13^, alcohol consumption^14^, and, generally, in epidemiologic studies^8^. Additionally, theoretical demonstrations^15^ claim that opinions about vaccination are generally not randomly distributed in human networks but clustered.

Surprisingly, to our knowledge, analyzing opinions about COVID-19 vaccination using a social network perspective has proved nonexistent in the literature. Therefore, our paper examines the role of human networks in understanding and predicting opinions about COVID-19 vaccination. Specifically, our main research objective in this study is to assess whether assortativity positively contributes to predicting COVID-19 opinions. In this direction, we regard *personal networks* (individuals, their direct social contacts, and the interconnections among them) as the immediate social contexts embedding the individuals^16^.

We analyze the personal networks of 443 individuals (*egos*), their social contacts (*alters*), and the tie configurations embedding the alters and surrounding the egos (we collected the information between March 16 and March 30, 2022, in Romania). These networks are of an equal number of alters (ten alters per network), amounting to 4430 alters. We examine if the opinions of the social contacts are clustered (by assortativity) or randomly distributed. Specifically, our study aims to assess the assortative mixing of opinions among the alters of the same ego. Thus, alters' opinion about the COVID-19 vaccination is the dependent variable. We use multilevel regression models to cope with the fact that alters are clustered by ego (some egos may be in communities with more positive/negative opinions; different egos may have a different opinion of what constitutes a positive/negative opinion, etc.).

The dataset comprises socio-demographic variables for egos and alters (individual attributes: sex, education, income, and age) and network variables (characteristics of the ego-alter ties and the alter-alter tie configurations). This unique collection of personal networks is also significant because it comes from a population (Romania) that has exhibited the second-lowest COVID-19 vaccination among European Union (EU) countries. As of March 13, 2023, according to the European Centre for Disease Prevention and Control, Romania (an Eastern European EU country) reported 42.6% of the population with at least one dose uptake and only 9.2% with the first booster uptake. Our findings suggest that opinions about COVID-19 vaccination are clustered in personal networks. Specifically, we illustrate that accounting for information about social contacts (ego's alters) brings new insights and allows for predicting COVID-19 vaccination opinions.

## Assortativity by COVID-19 vaccination opinions

We claim that current mainstream research can benefit from linking people to their surrounding social context^17^. In this fashion, we aim to detect assortativity in the social organization of opinions about COVID-19 vaccination. On the one hand, existing evidence^18^ advocates the role of *social contagion (influence)* in adopting innovation. Adopting an idea, a vaccine, or a technology is dependent on the proportion of surrounding people who have already adopted it^19^. Further, actors mutually influence and inform each other, increasing homogeneity within structural subgroups^20^. Human networks can be addressed as conduits for the circulation of intangible or tangible resources: from COVID-19 infections^11,21^, opinions^22^ and ideas^23,24^ to goods and other objects^25,26^. Thus, the pattern of networks is essential for understanding the flow of information^27^. Acquiring a trait may result from interacting with only one source (e.g., SARS-COV-2 spreading) or multiple active sources (e.g., opinion formation)^19^. Scholars argue that close friends and relatives are critical for complex contagion, whereas acquaintances are instrumental for the circulation of information over long social distances^28^.

On the other hand, human networks are not fixed but the object of renovations from the part of their embedded members. Social interactions are governed by *social selection*. Individuals tend to prefer to interact with others similar in a space of socio-demographic multi-dimensions^29,30^ (*homophily*). Additionally, *contextual influence* (e.g., sharing the same environment: country, community, etc.) can be pivotal in acquiring specific traits^31^.

According to the literature^32^, social influence (contagion), social selection (homophily), and contextual influence (confounding) can lead to assortative mixing. Namely, people live most of their time in clusters of similar peers wherein opinions are formed and reinforced.

Assortativity is the key variable in our models. Therefore, we limit detecting the positive contribution of assortativity to predicting opinions about COVID-19 vaccination (outcome variable). (Disentangling the factors responsible for assortativity is beyond the scope of our study.) We observe the behavior of our key variable by controlling for some other potentially relevant aspects of our real-world networks. We account for *network composition* (actor features such as age, education, sex, and income). Also, we employ *betweenness centrality* to measure a given actor's importance for the information flow between pairs of nodes^33^. This property identifies the nodes that control the circulation of opinions about vaccination in a network.

Next, the *network density* (how many possible ties are observed) gives information about the speed of opinion circulation. We expect high-density networks to exert a higher social control over their members. Furthermore, consequently, to enhance a specific opinion. Low scores lead to brokerage positions (people connecting social circles (peers) that otherwise remain disconnected). Then, the number of *components* (parts of the network completely disconnected from one another) indicates divisions in the structure and potential lines of cleavages (e.g., pro and against vaccination sub-groups). Last, *network centralization* (the tendency of a single node to be more central than all the other nodes) shows whether positional advantages have unequal distributions in personal networks. This structural analysis of the local neighborhoods shows how strategic network positions can be related to vaccination opinions (how the social texture affects individuals' opinions).

When predicting alters' opinions on vaccination, we employ multilevel analysis with random ego-level ("group-level") intercepts to account for two properties of our data. First, alters connected to the same ego will likely share unobserved variables (e.g., political attitudes, trust in institutions), which may influence their vaccination opinions. Second, all alters' variables are reported by their respective egos—and we could suspect that different egos have a different understanding of what constitutes a positive or negative opinion. By allowing the expected ratio of pro-vaccination alters to vary randomly by ego, our analysis essentially seeks to predict the difference of opinion among alters in the same personal network.

## Results

Tables 1 and 2 provide detailed descriptive statistics about the variables of interest. In Table 1, we note that our 443 respondents (egos) are preponderantly pro-vaccination (ƒ = 319; 72.0%), females (ƒ = 335; 75,6%), have higher education (ƒ = 285; 64.3%), and a monthly income between the national minimum and median salary (ƒ = 193; 43.6%). Overall, in their social contexts, the interviewees are surrounded by females (ƒ = 2717; 61.3%) and contacts who reportedly have pro-vaccination opinions (ƒ = 2895; 65.4%). Further, people with higher education are slightly prevalent in egos' networks (ƒ = 2226; 50.3%). Table 2 shows that respondents are relatively young (*Median* = 34.0 years old, *Range* = 56.0) and embedded in personal networks of durable relationships (*ego-alter tie duration: Mdn* = 23.0 years; *R* = 77.0). Their networks have only one component (*components: Mdn* = 1.0, *R* = 9.0), with a low degree of centralization (*centralization: Mdn* = 0.3, *R* = 0.7) and moderate density (*density: Mdn* = 0.4, *R* = 1.0).

**Table 1:** Descriptive statistics. Categorical variables of interest.

	Egos		Alters	
Opinions about COVID-19 vaccination				
Very good & good	319	72.01%	2895	65.35%
Very bad & bad	87	19.64%	1055	23.81%
Missing	37	8.35%	480	10.84%
Total	443	100.00%	4430	100.00%
Sex				
Male	108	24.38%	1713	38.67%
Female	335	75.62%	2717	61.33%
Missing	0	0.00%	0	0.00%
Total	443	100.00%	4430	100.00%
Higher education studies				
Yes	285	64.33%	2226	50.25%
No	158	35.67%	2204	49.75%
Missing	0	0.00%	0	0.00%
Total	443	100.0%	4430	100.00%
Income				
Less than minimum wage	78	17.61%		
In-between minimum & median wage	193	43.57%		
In-between median wage & median wage *plus* one minimum wage	145	32.73%		
More than median wage *plus* one minimum wage	27	6.09%		
Missing	0	0.00%		
Total	443	100.00%

**Table 2:** Descriptive statistics. Numeric variables of interest.

Egos						
	Age	Betweenness	Constraint	Centralization	Density	Components
Mean	36.47	24.07	0.32	0.31	0.47	1.67
Std.Dev	11.20	8.90	0.04	0.15	0.20	1.28
Min	19.00	0.00	0.10	0.00	0.00	1.00
Median	34.00	25.00	0.33	0.31	0.44	1.00
Max	75.00	45.00	0.38	0.73	1.00	10.00
Skewness	0.66	− 0.56	− 1.89	0.06	0.57	3.07
SE.Skewness	0.04	0.12	0.12	0.12	0.12	0.12
Kurtosis	− 0.17	0.24	5.40	− 0.17	0.24	12.56
N.Valid	443.00	443.00	443.00	443.00	443.00	443.00

Alters						
	Age	Betweenness	Ties to anti-vaccination peers	Ties to pro-vaccination peers	Degree	Ego alter tie duration (years)
Mean	41.71	2.14	1.04	2.90	4.19	22.82
Std.Dev	15.05	4.82	1.48	2.23	2.49	14.99
Min	18.00	0.00	0.00	0.00	0.00	1.00
Median	40.00	0.00	0.00	3.00	4.00	23.00
Max	90.00	34.00	9.00	9.00	9.00	78.00
Skewness	0.57	3.17	1.81	0.74	0.32	0.34
SE.Skewness	0.04	0.04	0.04	0.04	0.04	0.04
Kurtosis	− 0.29	10.74	3.49	0.00	− 0.74	− 0.66
N.Valid	4430.00	4430.00	4212.00	4212.00	4430.00	4430.00

Looking at the 4430 alters (Table 2), their median age is similar to the one of the egos (*Mdn* = 40.0; *R* = 72.0), which may express an age selection effect^34^. Given the high number of alters in each network (*Mean* = 6.5, *Std. Dev.* = 3.0, *Mdn* = 7, *R* = 10), the number of ties to peers that are in favor of COVID-19 vaccination ('ties to pro-vaccination peers', *M* = 2.9, *SD* = 2.2, *Mdn* = 3.0, *R* = 9.0) is higher than the one to peers that are against ('ties to anti-vaccination peers', *M* = 1.0, *SD* = 1.5, *Mdn* = 0.0, *R* = 9.0). Further, alters have, on average, 4.2 ties (*SD* = 2.5, *Mdn* = 4.0, *R* = 9.0) and display low betweenness scores (*M* = 2.1, *SD* = 4.8, *Mdn* = 0.0, *R* = 34.0). We emphasize that the maximum number of ties that an alter can have in a personal network (the degree centrality of an alter) is nine (all personal networks have ten alters). Visualizations of the descriptive statistics are available in the Supplementary Material.

In Table 3, we report the results of the multilevel logistic regression models fitted to predict alters' opinions about COVID-19 vaccination. And specifically, to detect evidence of possible assortativity effects in personal networks.

**Table 3:** Multilevel logistic regression models explaining alters' opinions about vaccination.

	Model 0 ('intercept')		Model 1 ('attributes')		Model 2 ('network')		Model 3 ('full')	
	Log-odds (95% CI)	OR (p)	Log-odds (95% CI)	OR (p)	Log-odds (95% CI)	OR (p)	Log-odds (95% CI)	OR (p)
alter sex			0.08 (− 0.12; 0.27)	1.08 (0.435)			0.11 (− 0.08; 0.31)	1.12 (0.260)
alter edu			**0.46 (0.25; 0.67)**	**1.58 (< 0.001)**			**0.44 (0.23; 0.65)**	**1.55 (< 0.001)**
alter age			**0.15 (0.04; 0.26)**	**1.16 (0.005)**			**0.16 (0.04; 0.28)**	**1.17 (0.008)**
ego sex			0.08 (− 0.27; 0.42)	1.08 (0.658)			0.06 (− 0.29; 0.41)	1.06 (0.739)
ego edu			− 0.28 (− 0.61; 0.05)	0.75 (0.094)			− 0.28 (− 0.62; 0.06)	0.75 (0.102)
ego income			0.18 (− 0.01; 0.38)	1.20 (0.069)			0.19 (− 0.01; 0.39)	1.21 (0.065)
ego age			0.05 (− 0.11; 0.20)	1.05 (0.561)			0.04 (− 0.12; 0.20)	1.04 (0.612)
ego covid			**2.14 (1.80; 2.48)**	**8.48 (< 0.001)**			**2.12 (1.77; 2.46)**	**8.30 (< 0.001)**
ego alter duration					0.05 (− 0.05; 0.15)	1.05 (0.324)	0.00 (− 0.12; 0.12)	1.00 (0.997)
assortativity					**0.30 (0.21; 0.38)**	**1.34 (< 0.001)**	**0.27 (0.19; 0.36)**	**1.31 (< 0.001)**
alter betw					0.07 (− 0.03; 0.17)	1.07 (0.156)	0.10 (0.00; 0.20)	1.10 (0.058)
comp					0.03 (− 0.18; 0.25)	1.04 (0.758)	0.09 (− 0.09; 0.27)	1.10 (0.312)
dens					0.05 (− 0.19; 0.28)	1.05 (0.696)	0.09 (− 0.10; 0.28)	1.10 (0.345)
centraliz					0.03 (− 0.18; 0.24)	1.03 (0.809)	0.03 (− 0.14; 0.20)	1.03 (0.736)
Intercept	**1.46 (1.28; 1.65)**	**4.32 (< 0.001)**	**− 0.69 (− 1.17; − 0.20)**	**0.50 (0.005)**	**1.47 (1.28; 1.66)**	**4.36 (< 0.001)**	**− 0.66 (− 1.16; − 0.17)**	**0.51 (0.008)**
Num. obs	3588		3588		3588		3588	
Num. groups: ego_id	401		401		401		401	
ICC	0.414		0.260		0.412		0.265	
AIC	3616.287		3462.61		3576.006		3428.7	
BIC	3628.658		3524.464		3625.489		3527.665	
Log Likelihood	− 1806.144 (df = 2)		− 1721.305 (df = 10)		− 1780.003 (df = 8)		− 1698.35 (df = 16)	
Var (SD): ego_id (Intercept)	2.323 (1.524)		1.153 (1.074)		2.305 (1.518)		1.183 (1.088)	

Significant values are in bold.

We find that alters with a higher level of education (*Model 1,* OR 1.58, 95% CI 1.28, 1.95, *p* < 0.001; *Model 3,* OR 1.55, 95% CI 1.26, 1.91, *p* < 0.001) and older alters (*M1,* OR 1.16, 95% CI 1.05, 1.29, *p* = 0.005; *M3*, OR 1.17, 95% CI 1.04, 1.32, *p* = 0.008) are more likely to have a positive opinion on COVID-19 vaccination. The only significant effect among the network-based covariates is the assortativity variable, which is consistently positive (*M2,* OR 1.34, 95% CI 1.24, 1.46, *p* < 0.001; *M3:* OR 1.31, 95% CI 1.21, 1.43, *p* < 0.001). This indicates that alters with similar opinions cluster together. Namely, people are more likely to have a positive attitude if their neighbors, on average, have a more positive attitude than the average attitude in the network. We also find that ego's opinion about vaccination makes a statistically significant contribution in predicting alters' opinions (*M1,* OR 8.48, 95% CI 6.03, 11.94, *p* < 0.001; *M3:* OR 8.30, 95% CI 5.87, 11.73, *p* < 0.001). We note that our multilevel models are mixed-effects models since they contain both random effects (namely the ego-specific intercept) and fixed effects (all other parameters in Table 3).

We assess the robustness of our results (Table 3) by fitting standard logistic regression models without any multilevel structure (Table 4). In these models, the additional variable *proportion of alters that are pro-vaccination (excluding the alter of reference),* i.e., *prop vacc ex alter*, controls for the average opinion of all other alters in the network. Interestingly, the two families of models (the multilevel logistic and standard logistic regression models) qualitatively yield the same results for almost all effects. The only qualitative difference in the standard logistic regression models, compared to the multilevel models, is that in the "joint" model (*M3*, in Table 4), alters whose ego has a higher level of education are less likely to have a positive opinion (*M1,* OR 0.79, 95% CI 0.65–0.96, *p* = 0.021; *M3,* OR 0.80, 95% CI 0.65–0.98, *p* = 0.030). While this might seem strange at first glance, we have to consider that all alter data is reported by ego. The negative effect of ego's education could reveal a social prejudice that higher educated people think more often that their alters have a negative attitude toward vaccination. Similarly, in the multilevel logistic regression models (Table 3), ego's education has a negative effect. However, this is not statistically significant (*M1,* OR 0.75, 95% CI 0.54, 1.05, *p* = 0.094; *M3,* OR 0.75, 95% CI 0.54, 1.06, *p* = 0.102).

**Table 4:** (Standard) logistic regression models explaining alters' opinions about vaccination.

	Model 0 ('intercept')		Model 1 ('attributes')		Model 2 ('network')		Model 3 ('full')	
	Log-odds (95% CI)	OR (p)	Log-odds (95% CI)	OR (p)	Log-odds (95% CI)	OR (p)	Log-odds (95% CI)	OR (p)
alter sex			0.05 (− 0.13; 0.23)	1.05 (0.568)			0.09 (− 0.09; 0.28)	1.10 (0.318)
alter edu			**0.43 (0.25; 0.62)**	**1.54 (< 0.001)**			**0.41 (0.22; 0.60)**	**1.51 (< 0.001)**
alter age			**0.14 (0.04; 0.23)**	**1.15 (0.006)**			**0.14 (0.03; 0.25)**	**1.15 (0.013)**
ego sex			0.02 (− 0.19; 0.23)	1.02 (0.845)			− 0.00 (− 0.22; 0.21)	1.00 (0.988)
ego edu			**− 0.24 (− 0.44; − 0.04)**	**0.79 (0.021)**			**− 0.23 (− 0.43; − 0.02)**	**0.80 (0.030)**
ego income			0.02 (− 0.10; 0.14)	1.02 (0.719)			0.02 (− 0.10; 0.14)	1.02 (0.731)
ego age			− 0.03 (− 0.13; 0.07)	0.97 (0.549)			− 0.04 (− 0.15; 0.06)	0.94 (0.424)
ego covid			**0.72 (0.50; 0.94)**	**2.05 (< 0.001)**			**0.67 (0.44; 0.89)**	**1.95 (< 0.001)**
ego alter duration					0.04 (− 0.05; 0.12)	1.04 (0.412)	0.01 (− 0.09; 0.12)	1.01 (0.793)
assortativity					**0.28 (0.20; 0.35)**	**1.32 (< 0.001)**	**0.26 (0.18; 0.33)**	**1.30 (< 0.001)**
alter betw					0.08 (− 0.01; 0.18)	1.09 (0.081)	0.10 (0.00; 0.19)	1.10 (0.050)
comp					0.05 (− 0.06; 0.16)	1.05 (0.358)	0.08 (− 0.03; 0.19)	1.08 (0.175)
dens					0.07 (− 0.04; 0.18)	1.07 (0.225)	0.09 (− 0.02; 0.21)	1.10 (0.107)
centraliz					0.03 (− 0.06; 0.13)	1.04 (0.491)	0.04 (− 0.06; 0.14)	1.04 (0.467)
prop vacc ex alter	**1.01 (0.93; 1.10)**	**2.74 (< 0.001)**	**0.82 (0.72; 0.92)**	**2.71 (< 0.001)**	**1.01 (0.93; 1.10)**	**2.75 (< 0.001)**	**0.84 (0.73; 0.94)**	**2.31 (< 0.001)**
Intercept	**1.27 (1.18; 1.36)**	**3.56 (< 0.001)**	**0.56 (0.23; 0.88)**	**3.27 (< 0.001)**	**1.28 (1.19; 1.37)**	**3.61 (< 0.001)**	**0.61 (0.28; 0.94)**	**1.84 (< 0.001)**
Num. obs	3588		3588		3588		3588	
AIC	3428.97		3377.527		3380.57		3338.17	
BIC	3441.34		3439.38		3430.053		3437.136	
Log Likelihood	− 1712.485 (df = 2)		− 1678.763 (df = 10)		− 1682.285 (df = 8)		− 1653.085 (df = 16)	
Deviance	3424.97		3357.527		3364.57		3306.17	

Significant values are in bold.

In Table 4, the additional control variable *prop vacc ex alter* (proportion of alters that are pro-vaccination, excluding the alter of reference), giving the average opinion among the alters in the same network (minus the alter of reference), has a strong positive effect on the attitude of the alter of reference. While this was expected (in fact, everything else would be a surprise), it underlines the need to control for the average opinion in the network. The multilevel models control for varying average opinions via the random ego-level intercepts.

## Discussion

Our study suggests that people with similar opinions about COVID-19 vaccination tend to cluster together (or be partitioned) in personal networks. We find assortativity to be a positive statistically significant effect (which directly contributes to our research objective). Namely, assortative mixing of opinions among the alters of the same ego. The likelihood of having a positive opinion about the COVID-19 vaccination increases when peers (neighbors) have, on average, a more positive attitude than the rest of the nodes in the network. Further, we discover a social correlation between the opinions held by egos (respondents) and alters (social contacts). This positive association suggests a possible ego-alter contagion effect or, alternatively, a social selection effect in the sense that egos tend to select alters with the same opinion. Unfortunately, our data do not permit a detailed examination of the causes of this assortative mixing. Future research and longitudinal data are needed to distinguish between social selection, contagion (or social influence), and confounding.

Our models also control for the attributes of the actors (education, sex, age) and their structural positions in the networks. We discover that alters with higher education^35^ and older^36^ are more likely to hold a positive opinion. Additionally, educated respondents (egos) think more often than those less educated that their social contacts (alters) have a negative opinion about vaccination. This result may indicate social prejudices (perceptions about the virtues of vaccination associated with education). Also, it might be a sign that egos with different levels of education have a different understanding of what constitutes a positive opinion. At the same time, the structural positions (node-level betweenness) and the organization of the relationships in personal networks (components, density, centralization) do not significantly contribute to predicting COVID-19 vaccination opinions.

Our readers should note several things regarding the interpretation of our results. An inherent feature of any network research design is that *egos* (respondents) report information about *alters* (their social contacts). Potentially, this can create biases percolating through the models and interpretations: false consensus effect^37^ (the tendency to see one's own choices as relatively common and appropriate) and inaccurate reports^38^. We included the duration of ego-alter ties (years) in our models (75% of all ego-alter ties have a duration of at least eight years). Also, we asked our respondents to provide information about those people with whom they communicate most often. At the same time, we employed two different statistical procedures (multilevel and standard logistic regression models) that eventually yielded similar findings. These remedies should counteract these biases' effects and improve the quality of the collected data. That is, we expect people who frequently interact over longer intervals to have more accurate data on their peers. Longitudinal cohort (balanced panel) data can improve control of the magnitude of these biases in the future. However, it cannot completely filter them out. Therefore, as a caveat, our findings may reflect the perceived clustering of vaccination opinions within networks as reported by the egos, which is relevant in its own right as perceptions can influence behavior. We acknowledge this limitation and suggest it as a direction for future research, proposing a design that includes the direct collection of vaccination opinions from both egos and alters. Also, future work can replicate our design in diverse settings and gather longitudinal data that may be beneficial in investigating related topics such as the evolution of opinion polarization. Additionally, when employed alongside Computer-Assisted Web Interviewing (CAWI), different data collection methodologies can provide valuable insights into potential biases inherent in relying solely on CAWI for data gathering.

In sum, our study claims that assortativity impacts COVID-19 vaccination opinions. Thus, we align to the stream of work that has already shown the role of assortativity in vaccination dynamics^8,39^ and status^40^ or disease spread^41^. In the special case of COVID-19 vaccination, in our sample, people with positive opinions declare having in their social proximity peers holding rather similar positive opinions. We suspect these results can be generalized to the whole population. We build on the growing evidence affirming not only the opinion clustering^42,43^ or social selection in the adoption of health behavior^44^ but also the clustered vaccination adoption (childhood vaccination refusals^45,46^, seasonal influenza vaccine uptake^47^, the imitation of vaccination behavior^48^). However, only future work could reveal if fixed network size leads to a biased mix of favorable and unfavorable vaccination opinions^49^. Our study can have broader implications that go beyond the specificity of the COVID-19 context and reach future pandemics. Understanding people's opinions on vaccination can act as an early warning system for potential outbreaks, inform predictions about vaccination uptake, or help supply chain management for vaccine distribution. Public health officials may address the challenges posed by assortativity in social networks and educational disparities by employing various strategies. For example, targeted communication strategies (e.g., social media campaigns tailored to specific social networks or communities), educational outreach programs (e.g., focused on areas with low levels of educational attainment), data-driven approaches and community engagement initiatives.

## Methods

### Study design, size, and selection of participants

We performed a real-world cross-sectional study and employed a personal network research design^50^ (*penet*). Using computer-assisted web interviewing, we collected questionnaire data from a random panel of 30,000 individuals (the panel was deemed representative of the Romanian population). Individuals were at least 18 years old when filling out the questionnaire and could speak the Romanian language (the questions were formulated in Romanian). We initiated the data collection process on March 16, 2022, and halted it on March 30, 2022 (we stopped due to the lack of new respondents). The actual data collection process was outsourced (yet the research team outlined and created the content of the research design). We sent invitations to all panel members to participate in the study (an invitation and two additional reminders via email). The invitations informed each potential participant about the research objectives and granted anonymity and the possibility of opting out at any moment (even after submitting the filled-out questionnaire). The email invitations also included the link to the questionnaire. The panel members participated in the study on a completely voluntary basis. The data collection process was single-stage. Individuals received an invitation to participate in the study and could choose to accept or decline. Those who agreed to participate were given immediate access to the self-administered questionnaire. This questionnaire included prompts, such as *'Please use acronyms or nicknames when mentioning alters'* to guide responses. An availability sample resulted in valid answers (questionnaires) from 896 respondents (dubbed *egos* according to the *penet* terminology). We note that the socio-demographic structure of our sample did not replicate the structure of the Romanian population.

We followed the conventional *penet* practice and organized the questionnaire into five components. (1) First, we addressed questions referring to the egos (socio-demographic items and opinions about COVID-19 vaccination). Then, (2) we included a *generator of alters* (persons connected to the ego). Each ego was required to elicit ten social contacts or alters (maximum five relatives and five friends). Namely, the egos were supposed to nominate people with whom they communicate most frequently. We restrained the number of alters to ten to avoid the respondent burden^51^. For example, a respondent (an ego) nominating ten alters must assess 45 pairs of alter-alter relationships, equating to 45 questions, i.e., (10*9)/2 = 45 pairs. If the number of nominated alters were to increase to 20, the number of required assessments will rise to 190 pairs ((20*19)/2 = 190). To maintain manageability and ensure high-quality data, we opted for a fixed number of alters, thus preventing undue burden on respondents, e.g., higher numbers of alters could be too time-consuming, mentally taxing, or otherwise overwhelming, potentially leading to lower response rates, incomplete data, or compromised data quality due to respondent fatigue or disengagement. Afterward, (3) we applied a *name interpreter*. We asked egos to report socio-demographic information about each of their nominated alters. Further, participants were asked about alters' opinions on COVID-19 vaccination. (4) We assessed alter-alter ties by inquiring whether each ego was aware of communication occurring between their respective alters in their absence (*Do [Alter X] and [Alter Y] contact each other independently of you?*). Namely, that was a question about the existence of relationships among alters as perceived by the respondent. Lastly, (5) we measured *ego-alter ties* in terms of duration (*For how many years do you know this alter?*). Notably, we instructed the egos to use acronyms or nicknames when eliciting information about alters (social contacts). In this way, we avoided disclosing the identity of the alters and harming them in any way. Additionally, we coached the respondents to devise the acronyms in such a way that it would allow them to respond to the alter-alter-tie questions.

Out of the 896 egos with valid responses, only 443 elicited the theoretical maximum number of ten alters. We kept this sub-sample of 443 egos for the statistical analysis and modeling. We excluded smaller ego-networks (network size less than ten) to ensure methodological consistency and comparability. This approach mitigated the potential for confounding effects that could arise from analyzing networks of varying sizes. (For instance, as shown elsewhere^19^, complex contagion is a function of the number of individuals that act as active social reinforcement sources; in our case, either pro or against COVID-19 vaccination. Researchers should be aware of several risks before replicating or performing a similar network data collection. Notably, we refer to low response rates, technical selection biases leading to non-representation (e.g., some groups may have limited access to internet), difficulties in capturing complex network relationships through standard survey methods, and the risk of skewed results due to response bias.

**Figure 1:**
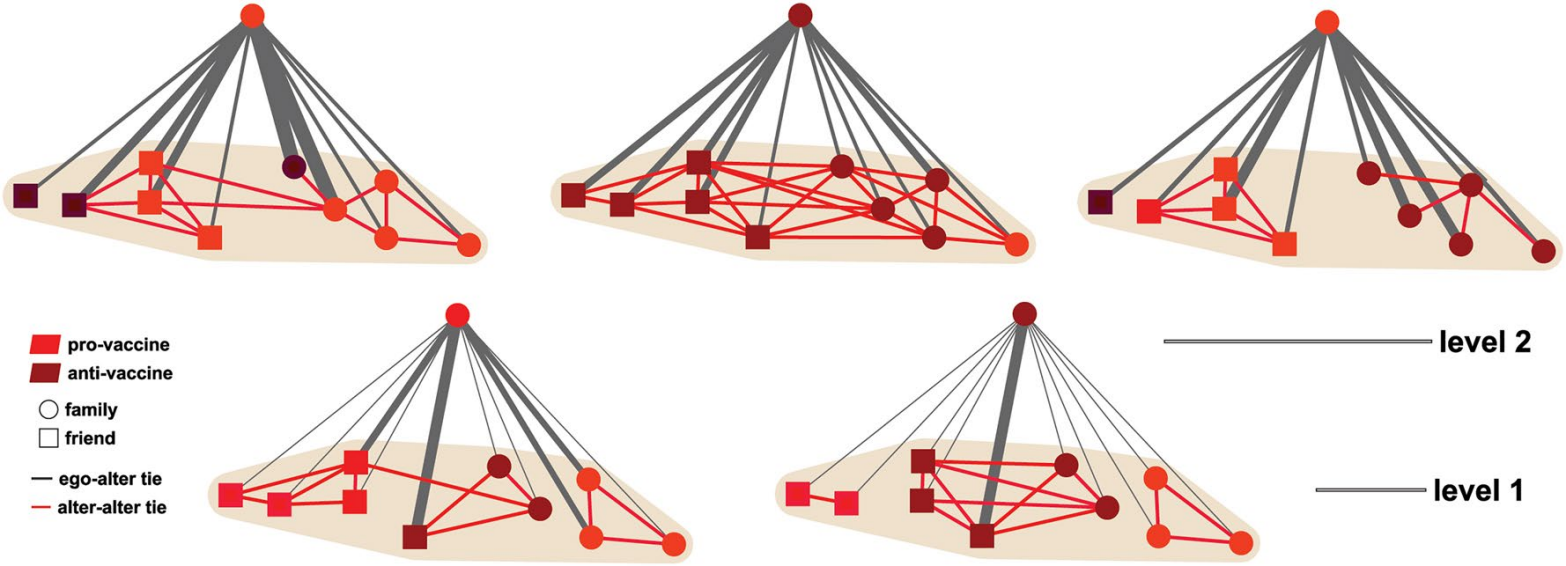
Networks resulted from the personal network research design. Ego-alter tie duration (in years) is marked by the tie thickness. Alters (level 1) are nested in egos (level 2). The horizontal lines indicating the data levels (level 1 and level 2) serve solely to denote these levels without reflecting any actual personal network connections.

Figure 1 provides an example of the personal networks that resulted after the administration of the questionnaire. First, we underline the multilevel (hierarchical) organization of the data: alters (*the first level*) are grouped by respondents (*the second level*). Next, we stress the existence of *within-network dependencies*: various patterns display how alters are interconnected. Also, we highlight the attributes describing *the nodes* (both egos and alters) and *the ties* (both ego-alter and alter-alter relationships). The variables included in Fig. 1 are only a selection for expository purposes.

### Variables

In this Section, we narratively describe our variables of interest, while in the following Section ('The 'assortativity' variable'), we present them in a summarized list format. We start the presentation of the variables used in our study with the *actor-level variables,* and then we continue with the *network-level variables*. We collected socio-demographic data referring to *sex* (0: males, 1: females), *education* (0: no higher education, 1: higher education), *age* (numerical, $\ge$ 18 years old), and *income* (0: less than minimum wage, 1: between minimum & median wage, 2: one minimum wage over the median wage, 3: more than one minimum wage over the median wage; the thresholds reflect Romanian national wages at the moment of data collection). For clarification purposes, the *education* variable in our study categorizes individuals into two groups: those who do not have a university degree, representing 'no higher education,' and those who possess a university degree, representing 'higher education.' We also measured *actors' opinions about COVID-19 vaccination*. Each study participant was required to answer the following question: *What opinion do you have about COVID-19 vaccination?* As stated in the presentation of the study design, we did not interview the alters (this is a common practice in personal network research studies). For this reason, we used a *proxy* to capture information about them. Namely, we requested egos to inform us about the socio-demographics (sex, age, education) and the opinions of each of their nominated alters about vaccination (*What opinion does Alter X have about COVID-19 vaccination?*). The questions concerning COVID-19 vaccination had the following pre-defined answers: *very bad, bad, good,* and *very good*. We intentionally omitted a neutral point given several methodological arguments, e.g., avoiding ambiguity, establishing a clear direction (either positive or negative), better discrimination among participants, and increasing engagement in providing answers. Later, we re-coded the responses into binary variables: either an ego (alter) has a *positive* (good or very good) or *negative* (bad or very bad) opinion vis-à-vis COVID-19 vaccination.

In terms of network-level measurements, first, we accounted for the *composition of the personal networks*. We computed the proportion of female alters, alters with higher education, alters with a positive opinion about COVID-19 vaccination, and the average age of the alters. We derived these variables from the actor-level variables (see the paragraph above). Then, we summarized *the properties of the ego-alter ties* in each personal network: the mean duration (in years). Further, we computed *node-level properties* for all the nodes (both egos and alters), such as Freeman's betweenness centrality (i.e., it quantifies the number of times a node acts as a bridge along the shortest path between two other nodes^33,52^). We also scrutinized the maximum scores of Freeman's betweenness centrality among the alters with a positive opinion about vaccination (i.e., people in favor of vaccination that connect social circles that otherwise would remain disconnected).

Lastly, we looked at the alter-alter ties and described the *overall structure of each personal network*. We calculated the density (the ratio between the number of observed ties and the number of theoretically possible ties), the centralization (i.e., the tendency of the ties in a network to revolve around a node; as the centralization score increases, this tendency becomes more pronounced^33^), and the number of components (i.e., a subset of the network's nodes (individuals, in our case) and the ties (relationships or interactions) connecting them, such that every node in the component is reachable from every other node within the same component, but not from nodes in different components). To accurately calculate the number of components containing alters within a personal network, we removed the ego. This is because the ego is connected to all alters; if we include egos in the analysis, their personal networks will consist of only one component^50^. A detailed discussion of the network measurements is available for the interested readership in the Supplementary Material.

### The 'assortativity' variable

Overall, for the analysis, we used data on ${n}_{{\text{e}}}$ = 443 egos and ${n}_{a}$ = 4430 alters. For alter $i$, let $j\left[i\right]$ denote the ego of $i$. Note that there is no overlap among the alters of different egos, so each alter is assigned to exactly one ego.

For each ego $j$, the binary variable ${y}_{j}^{\left(e\right)}$ indicates $j$’s vaccination opinion (1 for positive opinion; 0 for negative opinion). Likewise, for each alter $i$, the binary variable ${y}_{i}^{\left(a\right)}$ indicates $i$’s vaccination opinion (1 for positive opinion; 0 for negative opinion). In our analysis, we estimated models explaining alters' opinions ${y}_{i}^{\left(a\right)}$ and egos' opinions ${y}_{j}^{\left(e\right)}$ (dependent variables). Given the objectives of our study, we report here only the models predicting alters' opinions ${y}_{i}^{\left(a\right)}$. However, the models predicting egos' opinions ${y}_{j}^{\left(e\right)}$ are available in the Supplementary Material. Interested readers may consult these *ego models* if they look for further insights to contextualize the results reported in the body of the paper.

For ego $j$, $j$ = 1, …, ${n}_{e}$, we have a vector of covariates ${u}_{j}$, comprising the following covariates:

#### Attribute-level

- ${u}_{j1}$: ego's education ('ego edu')
- ${u}_{j2}$: ego's sex ('ego sex')
- ${u}_{j3}$: ego's income ('ego income')
- ${u}_{j4}$: ego's age ('ego age')

#### Network-level

- ${u}_{j5}$: proportion of female alters ('prop fem')
- ${u}_{j6}$: proportion of alters with higher education studies ('prop edu')
- ${u}_{j7}$: mean age of alters ('mean age')
- ${u}_{j8}$: proportion of alters in favor of vaccination ('prop vacc')
- ${u}_{j9}$: ego-alter mean duration (' mean duration')
- ${u}_{j10}$: ego-betweenness ('ego betw')
- ${u}_{j11}$: highest betweenness of an alter that is in favor of vaccination ('alter betw max pro')
- $${u}_{j12}$$: centralization ('centraliz')
- ${u}_{j13}$: network components ('comp')

For alter $i$*,* $i$ = 1,…, ${n}_{a}$*,* we have a vector of covariates ${x}_{i}$, comprising the following covariates:

#### Attribute-level

- ${x}_{i1}$: alter's education ('alter edu')
- ${x}_{i2}$: alter's age ('alter age')
- ${x}_{i3}$: alter's sex ('alter sex')
- moreover, ${x}_{i}$ contains all the ego covariates ${u}_{j\left[i\right]}$ and ego's opinion ${y}_{j}^{\left(e\right)}$ (note that these variables are well-defined since each alter $i$ is assigned to exactly one ego $j\left[i\right]$.

#### Network-level

- ${x}_{i4}$: proportion of alters in favor of vaccination, except for the alter of reference ('prop vacc ex alter')
- ${x}_{i5}$: duration of ego-alter ties ('ego alter tie duration')
- ${x}_{i6}$: alter covariate assortativity ('assortativity')
- ${x}_{i7}$: alter-betweenness ('alter betw')
- ${x}_{i8}$: number of components in a personal network ('components)
- ${x}_{i9}$: network density ('density)
- ${x}_{i10}$: network centralization ('centraliz)

Of particular relevance (and requiring additional explanation) is the alter covariate *assortativity variable* ('assortativity'), which tests whether alter's opinion is likely to be influenced by the opinions of those alters to which they are connected. Quantitatively, the assortativity variable indicates whether those alters connected to alter $i$ have, on average, a higher or a lower vaccination opinion than all of $j\left[i\right]$’s alters, different from $i$*.* More precisely, for alter $i$, let ${A}_{j\left[i\right]}^{\left(i\right)}$ be the set of alters of ego $j\left[i\right]$, different from $i$*.* (Note that in our data, these are always exactly nine alters since the size of all personal networks is ten.) Let ${N}_{i}$ be the *neighbors* of alter $i$*,* that is, those other alters of ego $j\left[i\right]$ who are connected to $i$ by an alter-alter tie. Then, for an alter $i$ with $\left|{N}_{i}\right|>0$ (that is, excluding the isolated alters), we define the assortativity variable to be the difference between the average opinion of $i$*’*s neighbors and the average opinion among all of $j\left[i\right]$’s alters, different from $i$*.* In formulas,

$${assortativity}_{i}=\frac{{\sum }_{{i}{\prime}\in {N}_{i}}{y}_{{i}{\prime}}^{\left(a\right)}}{\left|{N}_{i}\right|}-\frac{{\sum }_{{i}{\prime}\in {A}_{j\left[i\right]}^{\left(i\right)}}{y}_{{i}{\prime}}^{\left(a\right)}}{\left|{A}_{j\left[i\right]}^{\left(i\right)}\right|}$$

For alters with no neighbors (that is, isolated alters with $\left|{N}_{i}\right|=0$), we consider this variable as undefined and drop the respective alter from the analysis.

To provide an example, assume that among the nine alters of ego $j\left[i\right]$ that are different from $i$, six have a positive opinion. Further, assume that alter $i$ has alter-alter ties to four alters, among which two have a positive opinion. Then $i$*’s* assortativity variable would equal to $\frac{2}{4}-\frac{6}{9}=-\frac{1}{6}$. Indeed, $i$*’s* neighbors have a below average opinion for this network, justifying the negative value.

We emphasize that the definition of the assortativity variable for alter $i$ does not take into account $i$’s vaccination opinion to avoid circular dependency in the data.

The assortativity variable tells us whether $i$*’s* neighbors are more or less positive about vaccination than all the other alters of ego $j\left[i\right]$. The normalization obtained by subtracting the average opinion over the other alters is necessary since, without this normalization, the effect of this variable would be confounded by the overall ratio of positive opinion in $j\left[i\right]$’s network.

In a network with a high ratio of positive opinion, we would expect by chance alone that the average opinion among the neighbors of every alter is likely to lean on the positive side—and in addition, most alters in this network themselves are expected to have a positive opinion. Thus, without normalization, we would expect a positive correlation (taken over all the alters in our data) between $i$*’s* opinion and the average opinion over $i$*’s* neighbors—even if there is no assortativity of opinion present. (As a simple exercise, we estimated models with the average opinion of $i$*’s* neighbors as a covariate and found a strong positive effect—which we claim to be a useless finding since we cannot tell whether that effect indicates assortativity or just a correlation of opinion caused by varying positive ratios over the egos.) By the normalization, we capture in the assortativity variable whether $i$*’s* neighbors are more or less positive than the rest of the alters in the same network.

### Statistical models

We estimated models explaining alters’ opinion about COVID-19 vaccination, ${y}_{i}^{\left(a\right)}$, via multilevel logistic regression with random ego-level intercepts^53^. The multilevel approach is necessary to account for two characteristics of our data. First, alters are clustered within egos (see the *study design* previously presented). Therefore, alters of the same ego might have similar values in unobserved covariates, e.g., political attitude, trust in institutions, etc. Second, alters’ opinions are always reported by ego, and it is questionable whether all egos have the same understanding of "positive" or "negative" opinions. In fact, by the multilevel approach, we do not attempt to explain the absolute level of opinion of the alters—but rather whether alters have a more or less positive opinion compared to the other alters in the same network. An alternative approach using fixed (rather than random) ego-level intercepts is not feasible since there are egos in whose networks no alter or all alters have a positive opinion—which would theoretically lead to ego-level intercepts that are minus infinity or infinity, respectively. Formally, the multilevel logistic regression models specify, for each alter $i$ = 1, …, ${n}_{a}$, the probability that $i$ has a positive opinion via

$$logitPr\left({y}_{i}^{\left(a\right)}=1\right)={\alpha }_{j\left[i\right]}+\beta {x}_{i}$$

$${\alpha }_{j} \sim N\left({\mu }_{e},{\sigma }_{e}^{2}\right)$$

In the first equation, the probability that alter $i$ has a positive opinion is the logistic transformation of the ego-level intercept ${\alpha }_{j\left[i\right]}$ plus the sum over the values in the covariate vector ${x}_{i}$, multiplied with the values in the parameter vector $\beta$. In the second equation, the ego-level intercepts ${\alpha }_{j}$, for $j$ = 1, …, ${n}_{e}$, are assumed to be drawn from a normal distribution with mean ${\mu }_{e}$ and variance ${\sigma }_{e}^{2}$. Given the data, we estimate ${\mu }_{e}$, ${\sigma }_{e}^{2}$, ${\alpha }_{j}$, for $j$ = 1, …, ${n}_{e}$, and the parameter vector $\beta$ with the function *glmer* from the R package *lme4*^54^.

As an alternative to the multilevel models described, we fit standard logistic regression models that have no ego-level intercept but instead include the average opinion over all other alters $\frac{{\sum }_{{i}{\prime}\in {A}_{j\left[i\right]}^{\left(i\right)}}{y}_{{i}{\prime}}^{\left(a\right)}}{\left|{A}_{j\left[i\right]}^{\left(i\right)}\right|}$ (compare with the discussion of the assortativity variable) as an additional predictor for explaining the alter’s opinion ${y}_{i}^{\left(a\right)}$. This additional variable *prop vacc ex alter* controls for the average opinion of all other alters in the network (see “Results” Section). By doing so, we explain the difference between the opinion of alter $i$ and that of all other alters. In other words, we explain why some alters have a higher or lower opinion than the other alters in the same network. We believe the multilevel models are a better and more principled approach for our data. On the other hand, the pure logistic regression models (without any multilevel structure) are easier to understand and probably more common. In any case, it is interesting to compare the results yielded by the two classes of models.

Additionally, we estimated standard logistic regression models to predict egos’ opinions about COVID-19 vaccination, ${y}_{j}^{\left(e\right)}$. These models are not directly relevant to the objectives of our study. Yet, it may be useful for further insights into the general topic of opinions about vaccination. For this reason, these models are included and presented in the Supplementary Material.

The dataset, the entire code, and all the statistical analyses (diagnostics, further information about the variables included in the models and detailed results) are open access^55^ and available for replication (see also Supplementary Material). Moreover, we annotated the code (R script) with explanatory comments and spatially arranged it for human readability. In the code, we made the statistical models exactly reproducible by pseudo-random number generators, which are explicitly seeded.

### Statement

All methods were carried out in accordance with the relevant guidelines and regulations (specifically, those provided by the Romanian Sociologists Society, i.e., the Professional Association of Romanian Sociologists).

All the experimental protocol was approved by a named institutional/licensing committee. Specifically, the research protocol was approved by the Center for Innovation in Medicine Ethics Committee (2022/7/2 D) (Bucharest, Romania).

Informed consent was obtained from all subjects.

### Ethical declaration

The conducted research was performed in compliance with the Ethics Code of the University of Bucharest. The data collection process was outsourced. The authors analyzed raw data that were already anonymized. The research team received the data set from MEDNET Research based on the written accord no. 1 / 27.07.2022.

### Supplementary Information

Supplementary Information.

## Data Availability

The dataset analyzed in the current study is available in the *figshare* repository https://doi.org/10.6084/m9.figshare.22309174.v1.
