# Supplementary material for: Assortative mixing of opinions about COVID-19 vaccination in personal networks
Source: Sci Rep. 2024 Feb 9;14:3385. doi: 10.1038/s41598-024-53825-3 (PMC10858210; doi:10.1038/s41598-024-53825-3)
Supplement: Supplementary file 1 — Supplementary Information. [file 41598_2024_53825_MOESM1_ESM.pdf]

Supplementary material: Assortative mixing of opinions about COVID-19 vaccination in personal networks

Hâncean, MG, Lerner, J, Perc, M, Molina, JL, Geantă, M

2024-02-01

Contents

1. Read data . . . 3
    1.1 Alter data frame . . . 3
    1.2 Ego data frame . . . 7
    1.3 Alter-alter data frame . . . 10
2. Predicting alters' opinions about COVID-19 vacc. . . . 11
    2.1 Descriptive statistics . . . 11
        2.1.1 Factor variables . . . 11
        2.1.2 Numeric variables . . . 14
        2.1.3 Group by egos (personal networks) . . . 17
            Categorical . . . 17
            Visualizations . . . 30
            Numeric . . . 36
    2.2 Alter analysis . . . 37
        2.2.1 The models . . . 37
            Model 0 ('null model') . . . 37
            Model 1 ('attributes model') . . . 40
            Model 2 ('network model') . . . 44
            Model 3 ('full model') . . . 47
        2.2.2 Print other results . . . 53
            Log-likelihood, AIC, BIC . . . 53
            Confidence intervals for the estimates . . . 55
            Model deviance . . . 56
            Model comparison . . . 56
            Multi-collinearity . . . 58
            Model fit assessment . . . 59
3. Predicting egos' opinions (optional analysis) . . . 61
    3.1 Descriptive statistics . . . 61
        3.1.1 Factor variables . . . 61
        3.1.2 Numeric . . . 62
    3.2 Egos' analysis . . . 64
        3.2.1 The ego-models . . . 64
            Ego-Model 1 ('attributes model') . . . 64
            Ego-Model 2 ('network model') . . . 65
            Ego-Model 3 ('full model') . . . 66
        3.2.2 Print other results . . . 67
4. Network-level measurements (auxiliary section) . . . 72
    4.1 Components . . . 72
    4.2 Density . . . 72

4.3 Centralization . . . 72
4.4 Betweenness centrality . . . 72
4.5 Computing network-level variables . . . 72
5. Model evaluation . . . 75
5.1 Plotting the estimates from our models of interest . . . 75
5.2 Model interpretation . . . 75
5.3 Model comparison interpretation . . . 80
5.4 Predictive performance . . . 80
5.5 Multi-collinearity check . . . 83
5.6 Statistical tests for variable inclusion . . . 84
5.7 Wald test . . . 90

1. Read data

We employ a personal network research design. We read three data set files that comprise: *data about alters* (n = 4,430), *egos* (n = 443), and *alter-alter ties* (n = 9,274). Namely, we are looking at a collection of 443 personal networks of equal size (n = 10). Within each personal network, we have ten alters (five family members and five friends).

```
library(readxl)
library(tidyverse)
library(texreg)
```

1.1 Alter data frame

We read the alter-alter data frame. Then, transform variables into numeric variables.

```
alter.df1 <- read.csv("alter.df.csv")
```

```
alter.df2 <- alter.df1 %>%
  mutate_at(c("alter.covid",
              "ego_id",
              "alter_id",
              "alter.sex",
              "alter.edu",
              "alter.age",
              "ego.alter.duration",
              "alter.deg",
              "ties.to.pro.vacc",
              "ties.to.anti.vacc",
              "alter.betw",
              "alter.const",
              "ego.sex",
              "ego.edu",
              "ego.age",
              "ego.income",
              "ego.income.cat",
              "ego.covid",
              "comp",
              "dens",
              "centraliz",
              "alters.pro.vacc",
              "alters.anti.vacc",
              "alters.na.vacc",
              "prop.vacc"),
            as.numeric)
```

The labels of the variables designate the following:

Attributes about alters

- "alter.covid" = whether alter has a good & very good opinion (1) about vaccination or bad & very bad (0).
- "alter_id" = alter's id.
- "alter.sex" = alter's sex: 0 (male), 1 (female).
- "alter.edu" = alter's education: 0 (no higher edu), 1 (higher education).
- "alter.age" = alter's age (numerical variable: >= 18 years-old).

- "ego.alter.duration" = ego-alter tie duration (in years).
- "alter.deg" = number of ties an alter has in the personal network.
- "ties.to.pro.vacc" = number of ties an alter has, in the personal network, to peers who are in favor of COVID-19 vaccination.
- "ties.to.anti.vacc" = number of ties to others who are against COVID-19 vaccination.
- "alter.betw" = alter's betweenness (Freeman's centrality measure).

Attributes about egos

- "ego_id" = ego's id.
- "ego.sex" = ego's sex: 0 (male), 1 (female).
- "ego.edu" = ego's education: 0 (no higher education), 1 (higher education)
- "ego.age" = ego's age (numerical variable: >= 18 years old)
- "ego.income" = ego's income: 0 (below median), 1 (above median)
- "ego.income.cat" = ego's income: 0 (Less than minimum wage), 1 (In-between minimum & median wage), 2 (In-between median wage & median wage plus one minimum wage), 3 (More than median wage plus one minimum wage).
- "ego.covid" = whether ego has a good & a very good opinion (1) or a bad & a very bad opinion (0) about vaccination

Network-level structural properties

- "comp" = number of components in the personal network (ego is excluded)
- "dens" = density of the personal network (ego is excluded)
- "centraliz" = centralization of the personal network (ego is excluded).
- "alters.pro.vacc" = total number of pro-vaccination alters by network.
- "alters.anti.vacc" = total number of anti-vaccination alters by network.
- "alters.na.vacc" = total number of alters with no opinion, by network.
- "prop.vacc" = proportion of pro-vaccination alters by network (ego is excluded).

Next, we create new variables: proportion of ties to pro-(anti-)vaccination peers. It accounts for alter.deg & ties.to.pro.vacc (ties.to.anti.vacc).

```
# proportion of peers that are pro-vaccine
alter.df2 <- alter.df2 %>%
  mutate(prop.pro.vacc.ties = ties.to.pro.vacc / alter.deg)

# proportion of peers that are against-vaccine
alter.df2 <- alter.df2 %>%
  mutate(prop.anti.vacc.ties = ties.to.anti.vacc / alter.deg)
```

We subtract the overall proportion of pro-vaccine alters in each network from the proportion of ties to pro-vacc. alters (i.e., normalization of prop.pro.vacc.ties)

```
alter.df2 <- alter.df2 %>%
  mutate(assortativity.var = prop.pro.vacc.ties - (10*prop.vacc-alter.covid)/9)
```

We compute the proportion of pro-vaccine alters in a network, without the alter of reference.

```
alter.df2 <- alter.df2 %>%
  mutate(prop.vacc.ex.alter = (10*prop.vacc-alter.covid)/9)
```

We subtract the overall proportion of anti-vaccine alters in each network from the proportion of ties to anti-vacc. alters (i.e., normalization of prop.anti.vacc.ties)

```
alter.df2 <- alter.df2 %>%
  mutate(assortativity.neg.var = prop.anti.vacc.ties - 1 + (10*prop.vacc-alter.covid)/9)
```

Finally, we save only complete observations.

```
alter.df2.complete <- alter.df2[complete.cases(alter.df2), ]
nrow(alter.df2.complete) # number of observations in the data frame
```

```
## [1] 3588
```

```
alter.df2.complete$ego_id <- as.character(alter.df2.complete$ego_id)
alter.df2.complete$alter_id <- as.character(alter.df2.complete$alter_id)
```

We standardize the variables (mean = zero, std dev = one)

```
alter.df3 <- alter.df2.complete%>%
  mutate_at (c("alter.age",
              "ego.alter.duration",
              "alter.deg",
              "ties.to.pro.vacc",
              "ties.to.anti.vacc",
              "alter.betw",
              "alter.const",
              "ego.age",
              "comp",
              "dens",
              "centraliz",
              "prop.anti.vacc.ties",
              "prop.pro.vacc.ties",
              "assortativity.var",
              "prop.vacc.ex.alter",
              "assortativity.neg.var"
  ),
  ~(scale(.) %>% as.vector))
```

We check the standardization

```
alter.df4 <- alter.df3 %>% select(alter.age,
                                  ego.alter.duration,
                                  alter.deg,
                                  ties.to.pro.vacc,
                                  ties.to.anti.vacc,
                                  alter.betw,
                                  alter.const,
                                  ego.age,
                                  comp,
                                  dens,
                                  centraliz,
                                  prop.anti.vacc.ties,
                                  prop.pro.vacc.ties,
                                  assortativity.var,
                                  prop.vacc.ex.alter,
                                  assortativity.neg.var)
```

We compute means over standardized variables

```
means <- colMeans(alter.df4[sapply(alter.df4, is.numeric)], na.rm = TRUE)
hist(means,
     main = 'Standardized variables',
     col = "red",
     xlab = 'means',
     ylim = c(0, 16)) # all scores are very closed to zero
```

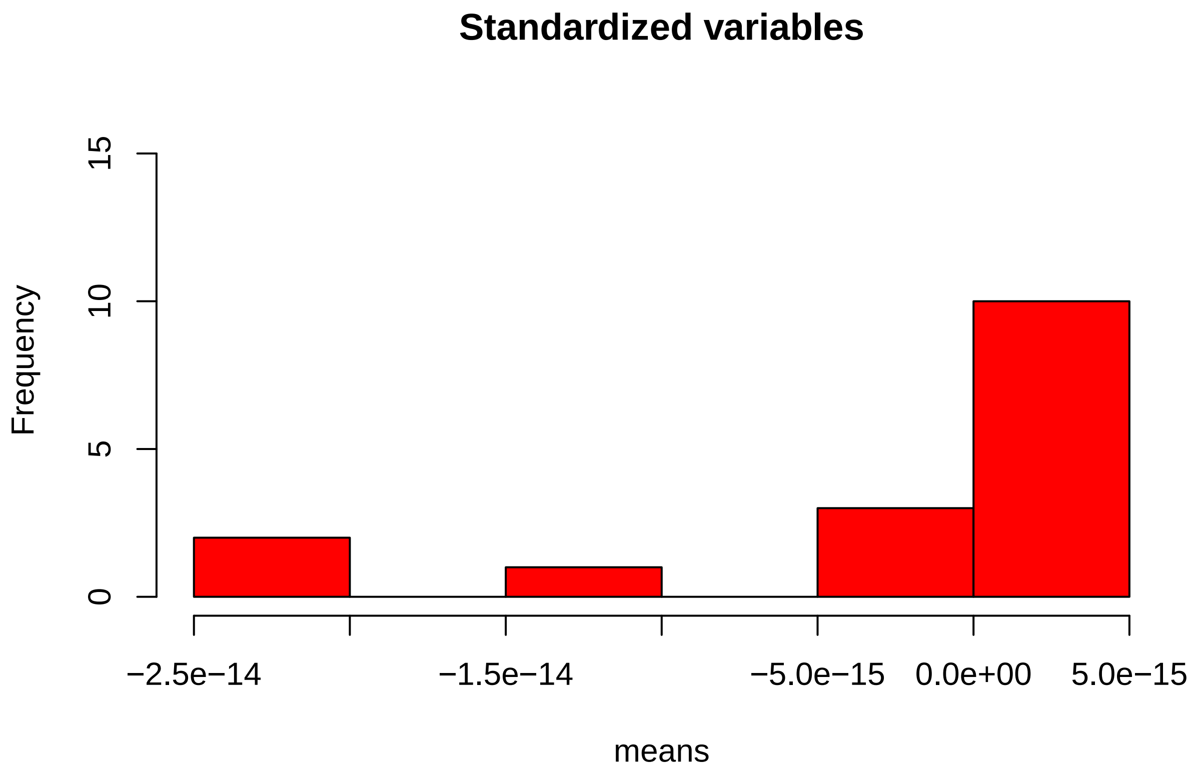

```
dev.off()
```

All variables have a standard deviation of one

```
apply(numeric.variables <- select_if(alter.df4, is.numeric),2, sd, na.rm=TRUE)
```

```
##              alter.age    ego.alter.duration             alter.deg
##                      1                     1                     1
##       ties.to.pro.vacc     ties.to.anti.vacc            alter.betw
##                      1                     1                     1
##            alter.const               ego.age                  comp
##                      1                     1                     1
##                   dens            centraliz   prop.anti.vacc.ties
##                      1                     1                     1
##     prop.pro.vacc.ties     assortativity.var    prop.vacc.ex.alter
##                      1                     1                     1
## assortativity.neg.var
##                      1
```

1.2 Ego data frame

We read the ego data frame. Then, transform variables into numeric variables.

```
ego.df <- read.csv("ego.attr.csv")
```

```
ego.df <- ego.df %>%
  mutate_at(c(
    "ego.sex"
    ,"ego.age"
    ,"ego.edu"
    ,"ego.income.cat"
    ,"ego.covid"
    , "ego.constraint"
    , "ego.betw"
    , "alter.const.max.pro"
    , "alter.betw.max.pro"
    , "mean.age"
    , "mean.duration"
    , "ego.alter.close"
    , "centraliz"
    , "dens"
    , "comp"
    , "prop.fem"
    , "prop.edu"
    , "prop.vacc"
  ), as.numeric)
```

The labels of the variables designate the following:

Ego's attributes

- "ego.sex" = ego's sex: 0 male, 1 female
- "ego.age" = ego's age: >= 18 years old
- "ego.edu" = ego's education: 0 = no higher edu., 1 = higher edu.
- "ego.income.cat" = ego's income: 0 (Less than minimum wage), 1 (In-between minimum & median wage), 2 (In-between median wage & median wage plus one minimum wage), 3 (More than median wage plus one minimum wage)
- "ego.covid" = whether ego has a 'good' or 'very good opinion' (1) or a 'bad' & 'very bad' opinion (0) about COVID-19 vaccination

Ego's positional features

- "ego.betw" = ego's (Freeman's) betweenness centrality score

Alter's positional attributes

- "alter.betw.max.pro" = the largest betweenness score displayed by a pro vaccination alter

Alters' attributes

- "mean.age" = mean of the alters' age in a personal network

Ego-alter tie features

- "mean.duration" = avg. duration (years) of ego-alter ties in a personal network

Network features (computed without the ego)

- "centraliz" = the centralization score of the personal network.
- "dens" = the personal network density.
- "comp" = number of components in the personal network.

Personal network composition

- "prop.fem" = proportion of female alters in a personal network.
- "prop.edu" = proportion of alters with higher education studies in a personal network
- "prop.vacc" = proportion of alters with a 'good' & 'very good' opinions about vaccination

```
ego.df <- ego.df %>% mutate_at("ego_id", as.character)
```

We standardize the numerical variables (mean = zero, std dev = one)

```
ego.df2 <- ego.df%>%
  mutate_at (c('prop.vacc',
               'mean.age',
               'prop.edu',
               'prop.fem',
               'dens',
               'centraliz',
               'mean.duration',
               'ego.age',
               'ego.betw',
               'alter.betw.max.pro',
               'comp'),
             ~(scale(.) %>% as.vector))
```

We check the standardization

```
ego.df3 <- ego.df2 %>%
  select(prop.vacc,
         mean.age,
         prop.edu,
         prop.fem,
         dens,
         centraliz,
         mean.duration,
         ego.age,
         ego.betw,
         alter.betw.max.pro,
         comp)
```

We compute means over standardized variables

```
means <- colMeans(ego.df3[sapply(ego.df3, is.numeric)],
                  na.rm = TRUE)
```

```
hist(means,
     main = 'Standardized variables',
     col = "red",
     xlab = 'means',
     ylim = c(0, 16)) # all scores are very closed to zero
```

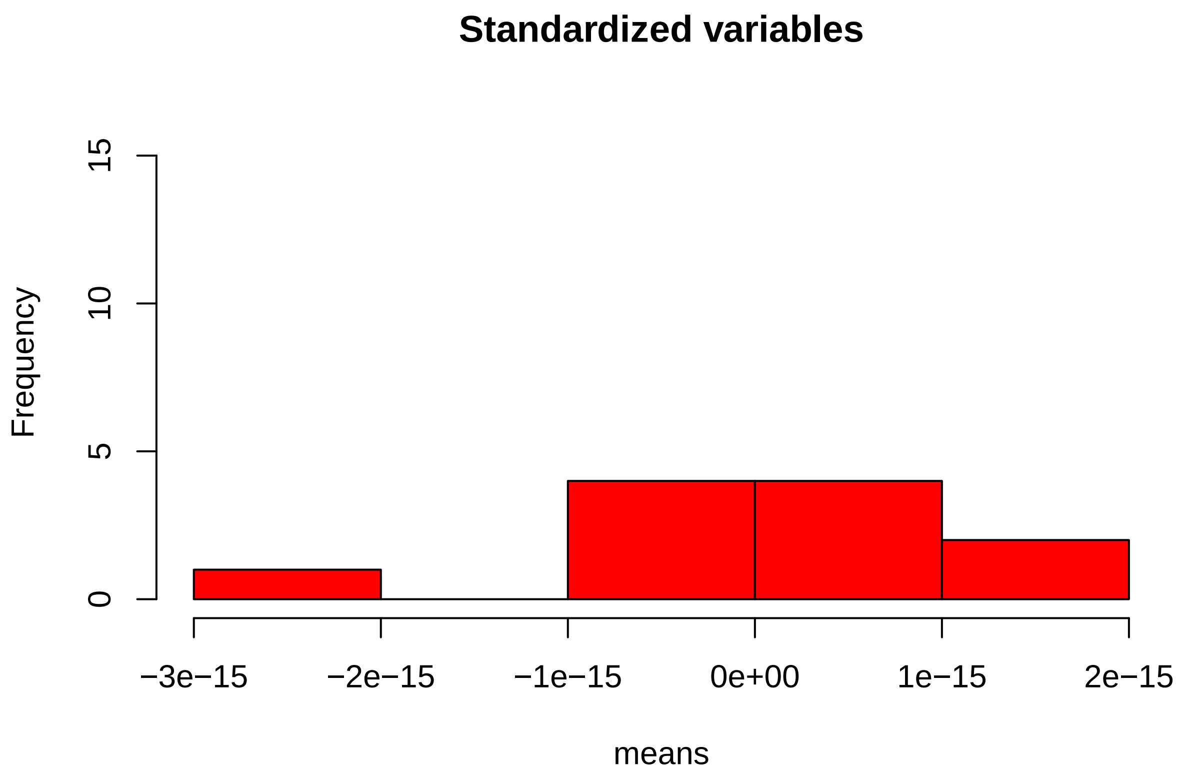

All standardized variables have standard deviation of one

```
apply(numeric.variables <- select_if(ego.df3, is.numeric),2,
      sd, na.rm=TRUE)
```

```
##          prop.vacc           mean.age           prop.edu           prop.fem
##                  1                  1                  1                  1
##               dens          centraliz      mean.duration            ego.age
##                  1                  1                  1                  1
##           ego.betw alter.betw.max.pro               comp
##                  1                  1                  1
```

```
dev.off()
```

We keep the complete observations

```
ego.df2.complete <- ego.df2[complete.cases(ego.df2), ]
nrow(ego.df2.complete) # number of observations in the data frame
```

```
## [1] 262
```

1.3 Alter-alter data frame

We read the alter-alter tie data frame.

```
aa.ties <- read.csv("aa.ties.df.csv")
```

2. Predicting alters' opinions about COVID-19 vacc.

Because we want to predict alters' opinions about COVID-19 vaccination, we will use the data frame containing information about each alter (i.e., observations are alters).

We use a shorter label for the dataset

```
dat <- alter.df2
nrow(dat) # number of rows in the dataset (n = 4,430)
```

```
## [1] 4430
```

```
library(summarytools)
```

2.1 Descriptive statistics

2.1.1 Factor variables We group all the factor variables into a special dataframe

```
dat.factor <- dat %>% select(alter.covid, alter.sex, alter.edu,
                             ego.sex, ego.edu, ego.income.cat, ego.covid)

dat.factor <- dat.factor %>%
  mutate_at(vars(alter.covid, alter.sex, alter.edu,
                 ego.sex, ego.edu, ego.income.cat,
                 ego.covid), factor)
```

```
# alter's opinion about COVID-19 vaccination
dat.factor$alter.covid <- factor(dat.factor$alter.covid,
                                 levels = c(0,1),
                                 labels = c("'bad & very bad' opinion",
                                            "'good & very good' opinion"))
freq(dat.factor$alter.covid)
```

```
## Frequencies
## dat.factor$alter.covid
## Type: Factor
##
##                                     Freq   % Valid   % Valid Cum.   % Total   % Total Cum.
## -------------------------------- ------ --------- -------------- --------- --------------
##         'bad & very bad' opinion   1055     26.71          26.71     23.81          23.81
##       'good & very good' opinion   2895     73.29         100.00     65.35          89.16
##                             <NA>    480                              10.84         100.00
##                            Total   4430    100.00         100.00    100.00         100.00
```

```r
# alter's sex
dat.factor$alter.sex <- factor(dat.factor$alter.sex,
                               levels = c(0,1),
                               labels = c("male", "female"))
freq(dat.factor$alter.sex)
```

```
## Frequencies
## dat.factor$alter.sex
## Type: Factor
##
##                Freq   % Valid   % Valid Cum.   % Total   % Total Cum.
## ------------ ------ --------- -------------- --------- --------------
##         male   1713     38.67          38.67     38.67          38.67
##       female   2717     61.33         100.00     61.33         100.00
##         <NA>      0                               0.00         100.00
##        Total   4430    100.00         100.00    100.00         100.00
```

```r
# alter's education
dat.factor$alter.edu <- factor(dat.factor$alter.edu,
                               levels = c(0,1),
                               labels = c("no - higher edu.",
                                          "yes - higher edu."))
freq(dat.factor$alter.edu)
```

```
## Frequencies
## dat.factor$alter.edu
## Type: Factor
##
##                           Freq   % Valid   % Valid Cum.   % Total   % Total Cum.
## ----------------------- ------ --------- -------------- --------- --------------
##        no - higher edu.   2204     49.75          49.75     49.75          49.75
##       yes - higher edu.   2226     50.25         100.00     50.25         100.00
##                    <NA>      0                               0.00         100.00
##                   Total   4430    100.00         100.00    100.00         100.00
```

```r
# ego's opinion about COVID-19 vaccination

dat.ego.factor <- ego.df # we use the ego dataset: ego-level variables
dat.ego.factor$ego.covid <- factor(dat.ego.factor$ego.covid,
                                   levels = c(0,1),
                                   labels = c("'very bad & bad' opinion",
                                              "'very good & good' opinion"))
freq(dat.ego.factor$ego.covid)
```

```
## Frequencies
## dat.ego.factor$ego.covid
## Type: Factor
##
##                                    Freq   % Valid   % Valid Cum.   % Total   % Total Cum.
## -------------------------------- ------ --------- -------------- --------- --------------
##         'very bad & bad' opinion     87     21.43          21.43     19.64          19.64
##       'very good & good' opinion    319     78.57         100.00     72.01          91.65
##                             <NA>     37                               8.35         100.00
##                            Total    443    100.00         100.00    100.00         100.00
```

```r
# ego's sex
dat.ego.factor$ego.sex <- factor(dat.ego.factor$ego.sex,
                                 levels = c(0,1),
                                 labels = c("male", "female"))
freq(dat.ego.factor$ego.sex)
```

```
## Frequencies
## dat.ego.factor$ego.sex
## Type: Factor
##
##               Freq   % Valid   % Valid Cum.   % Total   % Total Cum.
## ------------ ------ ---------- -------------- ---------- --------------
##         male    108      24.38          24.38      24.38          24.38
##       female    335      75.62         100.00      75.62         100.00
##         <NA>      0                                 0.00         100.00
##        Total    443     100.00         100.00     100.00         100.00
```

```r
# ego's education
dat.ego.factor$ego.edu <- factor(dat.ego.factor$ego.edu,
                                 levels = c(0,1),
                                 labels = c("no - higher edu.",
                                            "yes - higher edu."))

freq(dat.ego.factor$ego.edu)
```

```
## Frequencies
## dat.ego.factor$ego.edu
## Type: Factor
##
##                           Freq   % Valid   % Valid Cum.   % Total   % Total Cum.
## ----------------------- ------ ---------- -------------- ---------- --------------
##        no - higher edu.    158      35.67          35.67      35.67          35.67
##       yes - higher edu.    285      64.33         100.00      64.33         100.00
##                    <NA>      0                                 0.00         100.00
##                   Total    443     100.00         100.00     100.00         100.00
```

```r
# ego's income
dat.ego.factor$ego.income.cat <- factor(dat.ego.factor$ego.income.cat,
        levels = c(0, 1, 2, 3),
        labels = c("Less than minimum wage",
        "In-between minimum & median wage",
        "In-between median wage & median wage plus one minimum wage",
        "More than median wage plus one minimum wage"))

freq(dat.ego.factor$ego.income.cat)
```

```
## Frequencies
## dat.ego.factor$ego.income.cat
## Type: Factor
##
##                                                                       Freq   % Valid   % Valid Cum.   %
## ----------------------------------------------------------------- ------ --------- -------------- ---
##                                            Less than minimum wage     78     17.61          17.61
##                                  In-between minimum & median wage    193     43.57          61.17
##       In-between median wage & median wage plus one minimum wage    145     32.73          93.91
##                       More than median wage plus one minimum wage     27      6.09         100.00
##                                                              <NA>      0
##                                                             Total    443    100.00         100.00
```

2.1.2 Numeric variables We collect all numerical variables into a special dataframe object

```r
dat.alter.num <- dat %>%
  select(alter.age, alter.betw, alter.deg,
         ego.alter.duration, prop.pro.vacc.ties,
         assortativity.var, comp, dens, centraliz, prop.vacc,
         ties.to.pro.vacc, ties.to.anti.vacc,
         alters.pro.vacc, alters.anti.vacc, alters.na.vacc)
```

We report descriptive statistics (n = 4,430)

```
descr(dat.alter.num) # alter-level variables
```

```
## Descriptive Statistics
## dat.alter.num
## N: 4430
##
##                      alter.age   alter.betw   alter.deg   alters.anti.vacc   alters.na.vacc
## ----------------- ----------- ------------ ----------- ------------------ ----------------
##              Mean       41.71         2.14        4.19               2.38             1.08
##           Std.Dev       15.05         4.82        2.49               2.41             2.20
##               Min       18.00         0.00        0.00               0.00             0.00
##                Q1       30.00         0.00        2.00               0.00             0.00
##            Median       40.00         0.00        4.00               2.00             0.00
##                Q3       52.00         1.50        6.00               4.00             1.00
##               Max       90.00        34.00        9.00              10.00            10.00
##               MAD       16.31         0.00        2.97               2.97             0.00
##               IQR       22.00         1.50        4.00               4.00             1.00
##                CV        0.36         2.25        0.59               1.01             2.03
##          Skewness        0.57         3.17        0.32               1.07             2.62
##       SE.Skewness        0.04         0.04        0.04               0.04             0.04
##          Kurtosis       -0.29        10.74       -0.74               0.53             6.62
##           N.Valid     4430.00      4430.00     4430.00            4430.00          4430.00
##         Pct.Valid      100.00       100.00      100.00             100.00           100.00
##
## Table: Table continues below
##
##
##
##                      alters.pro.vacc   assortativity.var   centraliz      comp      dens
## ----------------- ----------------- ------------------- ----------- --------- ---------
##              Mean              6.53                0.01        0.31      1.67      0.47
##           Std.Dev              2.95                0.20        0.15      1.28      0.20
##               Min              0.00               -0.89        0.00      1.00      0.00
##                Q1              4.00               -0.06        0.22      1.00      0.33
##            Median              7.00                0.00        0.31      1.00      0.44
##                Q3              9.00                0.11        0.40      2.00      0.58
##               Max             10.00                0.89        0.73     10.00      1.00
##               MAD              2.97                0.13        0.13      0.00      0.19
##               IQR              5.00                0.17        0.18      1.00      0.25
##                CV              0.45               14.53        0.47      0.76      0.42
##          Skewness             -0.67               -0.45        0.06      3.08      0.57
##       SE.Skewness              0.04                0.04        0.04      0.04      0.04
##          Kurtosis             -0.56                3.37       -0.16     12.62      0.26
##           N.Valid           4430.00             3774.00     4430.00   4430.00   4430.00
##         Pct.Valid            100.00               85.19      100.00    100.00    100.00
##
## Table: Table continues below
##
##
##
##                      ego.alter.duration   prop.pro.vacc.ties   prop.vacc   ties.to.anti.vacc
## ----------------- -------------------- -------------------- ----------- -------------------
##              Mean                22.82                 0.67        0.65                1.04
```

```
##            Std.Dev             14.99                0.36        0.30                1.48
##                Min              1.00                0.00        0.00                0.00
##                 Q1              8.00                0.40        0.40                0.00
##             Median             23.00                0.78        0.70                0.00
##                 Q3             33.00                1.00        0.90                2.00
##                Max             78.00                1.00        1.00                9.00
##                MAD             19.27                0.33        0.30                0.00
##                IQR             25.00                0.60        0.50                2.00
##                 CV              0.66                0.54        0.45                1.43
##           Skewness              0.34               -0.70       -0.67                1.81
##        SE.Skewness              0.04                0.04        0.04                0.04
##           Kurtosis             -0.66               -0.92       -0.56                3.49
##            N.Valid           4430.00             4212.00     4430.00             4212.00
##          Pct.Valid            100.00               95.08      100.00               95.08
##
## Table: Table continues below
##
##
##
##                     ties.to.pro.vacc
## ----------------- ------------------
##              Mean               2.90
##           Std.Dev               2.23
##               Min               0.00
##                Q1               1.00
##            Median               3.00
##                Q3               4.00
##               Max               9.00
##               MAD               2.97
##               IQR               3.00
##                CV               0.77
##          Skewness               0.74
##       SE.Skewness               0.04
##          Kurtosis               0.00
##           N.Valid            4212.00
##         Pct.Valid              95.08
```

```r
descr(ego.df$ego.age) # ego-level variables
```

```
## Descriptive Statistics
## ego.df$ego.age
## N: 443
##
##                     ego.age
## ----------------- ---------
##              Mean     36.47
##           Std.Dev     11.20
##               Min     19.00
##                Q1     28.00
##            Median     34.00
##                Q3     44.00
##               Max     75.00
##               MAD     11.86
##               IQR     16.00
##                CV      0.31
```

```
##          Skewness      0.66
##       SE.Skewness      0.12
##          Kurtosis     -0.18
##           N.Valid    443.00
##         Pct.Valid    100.00
```

We compute the range for the variables

```
(range.alter.vars <- sapply(dat.alter.num,
               function(dat.alter.num) max(dat.alter.num, na.rm = TRUE) -
                              min(dat.alter.num, na.rm = TRUE)))
```

```
##           alter.age           alter.betw            alter.deg ego.alter.duration
##           72.000000            34.000000             9.000000          77.000000
## prop.pro.vacc.ties  assortativity.var                 comp               dens
##            1.000000             1.777778             9.000000           1.000000
##           centraliz            prop.vacc    ties.to.pro.vacc  ties.to.anti.vacc
##            0.730000             1.000000             9.000000           9.000000
##     alters.pro.vacc     alters.anti.vacc       alters.na.vacc
##           10.000000            10.000000            10.000000
```

2.1.3 Group by egos (personal networks) We report statistics computed within each personal network.

Categorical We gather all the categorical variables in a distinct dataframe object.

```
dat.factor <- dat %>% select(alter.covid, alter.sex, alter.edu,
                             ego.sex, ego.edu, ego.income.cat, ego.covid,
                             ego_id)

dat.factor <- dat.factor %>%
  mutate_at(vars(alter.covid, alter.sex, alter.edu,
                 ego.sex, ego.edu, ego.income.cat, ego.covid),
            factor)
```

```r
# alters' opinions about COVID-19 (proportions in each pers. netw.)
dat.factor$alter.covid <- factor(dat.factor$alter.covid,
        levels = c(0,1),
        labels = c("'bad & very bad opinion'",
        "'very good & good opinion'"))
prop.table(table(dat.factor$alter.covid, dat$ego_id), 2)
```

```
##
##                                     1         2         3         4         6
##    'bad & very bad opinion'   0.1000000 0.6666667 0.2000000 0.0000000 0.2000000
##    'very good & good opinion' 0.9000000 0.3333333 0.8000000 1.0000000 0.8000000
##
##                                     8         9        10        12        13
##    'bad & very bad opinion'   0.3000000 0.0000000 0.2000000 0.3750000 1.0000000
##    'very good & good opinion' 0.7000000 1.0000000 0.8000000 0.6250000 0.0000000
##
##                                    14        16        17        19        24
##    'bad & very bad opinion'   0.0000000 0.3000000 0.3000000 0.1428571 0.4444444
##    'very good & good opinion' 1.0000000 0.7000000 0.7000000 0.8571429 0.5555556
##
##                                    28        29        31        32        34
##    'bad & very bad opinion'   0.3333333 0.1250000 0.6000000 0.6000000 0.2000000
##    'very good & good opinion' 0.6666667 0.8750000 0.4000000 0.4000000 0.8000000
##
##                                    36        38        43        47        48
##    'bad & very bad opinion'   0.0000000 1.0000000 0.4000000 0.6000000 0.0000000
##    'very good & good opinion' 1.0000000 0.0000000 0.6000000 0.4000000 1.0000000
##
##                                    50        51        54        59        61
##    'bad & very bad opinion'   0.0000000 0.5000000 0.2222222 0.6666667 0.9000000
##    'very good & good opinion' 1.0000000 0.5000000 0.7777778 0.3333333 0.1000000
##
##                                    62        64        65        66        67
##    'bad & very bad opinion'   0.5000000 0.0000000 0.0000000 0.1000000 0.1000000
##    'very good & good opinion' 0.5000000 1.0000000 1.0000000 0.9000000 0.9000000
##
##                                    68        69        72        73        74
##    'bad & very bad opinion'   0.1000000 0.0000000 0.1000000 0.5555556 0.0000000
##    'very good & good opinion' 0.9000000 1.0000000 0.9000000 0.4444444 1.0000000
##
##                                    78        79        81        84        85
##    'bad & very bad opinion'   0.2000000 0.1000000 0.8000000 0.5000000 0.2000000
##    'very good & good opinion' 0.8000000 0.9000000 0.2000000 0.5000000 0.8000000
##
##                                    86        87        90        91 92
##    'bad & very bad opinion'   0.4000000 0.1000000 0.2222222 0.0000000
##    'very good & good opinion' 0.6000000 0.9000000 0.7777778 1.0000000
##
##                                    95        97        98        99       100
##    'bad & very bad opinion'   0.3000000 0.1000000 0.1111111 0.0000000 0.7000000
##    'very good & good opinion' 0.7000000 0.9000000 0.8888889 1.0000000 0.3000000
##
##                                   101       103       104       105       106
##    'bad & very bad opinion'   0.4000000 0.1111111 0.6000000 0.1000000 0.0000000
```

```
##   ’very good & good opinion’ 0.6000000 0.8888889 0.4000000 0.9000000 1.0000000
##
##                                    107       108       109       110       111
##   ’bad & very bad opinion’   0.0000000 0.6000000 0.9000000 0.0000000 0.8000000
##   ’very good & good opinion’ 1.0000000 0.4000000 0.1000000 1.0000000 0.2000000
##
##                                    112       113       114       115       118
##   ’bad & very bad opinion’   0.5000000 0.0000000 0.0000000 0.6000000 0.0000000
##   ’very good & good opinion’ 0.5000000 1.0000000 1.0000000 0.4000000 1.0000000
##
##                                    122       125       130       131       133
##   ’bad & very bad opinion’   0.0000000 0.0000000 0.9000000 0.3000000 0.0000000
##   ’very good & good opinion’ 1.0000000 1.0000000 0.1000000 0.7000000 1.0000000
##
##                                    138       139       140       142       143
##   ’bad & very bad opinion’   0.0000000 0.7500000 0.6666667 0.0000000 0.6666667
##   ’very good & good opinion’ 1.0000000 0.2500000 0.3333333 1.0000000 0.3333333
##
##                                    146       148       150       151       156
##   ’bad & very bad opinion’   0.1250000 0.1111111 0.0000000 0.2857143 0.4000000
##   ’very good & good opinion’ 0.8750000 0.8888889 1.0000000 0.7142857 0.6000000
##
##                                    157       158       161       164       165
##   ’bad & very bad opinion’   0.2222222 0.0000000 0.3000000 0.3333333 0.4000000
##   ’very good & good opinion’ 0.7777778 1.0000000 0.7000000 0.6666667 0.6000000
##
##                                    168       169       171       173       175
##   ’bad & very bad opinion’   0.2500000 0.1000000 0.3333333 1.0000000 0.3000000
##   ’very good & good opinion’ 0.7500000 0.9000000 0.6666667 0.0000000 0.7000000
##
##                                    178       179       181       184       185
##   ’bad & very bad opinion’   0.2000000 0.1000000 0.2500000 0.4000000 1.0000000
##   ’very good & good opinion’ 0.8000000 0.9000000 0.7500000 0.6000000 0.0000000
##
##                                    188       189       190       192       194
##   ’bad & very bad opinion’   0.1000000 0.2000000 0.6000000 0.4000000 0.1000000
##   ’very good & good opinion’ 0.9000000 0.8000000 0.4000000 0.6000000 0.9000000
##
##                                    198       199       201       202       204
##   ’bad & very bad opinion’   0.2000000 0.0000000 0.4000000 0.3000000 0.1000000
##   ’very good & good opinion’ 0.8000000 1.0000000 0.6000000 0.7000000 0.9000000
##
##                                    205       207 208       209       210
##   ’bad & very bad opinion’   0.6000000 0.2000000     0.2500000 0.1000000
##   ’very good & good opinion’ 0.4000000 0.8000000     0.7500000 0.9000000
##
##                                    211       213       216       218       219
##   ’bad & very bad opinion’   0.6250000 0.2000000 0.4000000 0.3000000 0.0000000
##   ’very good & good opinion’ 0.3750000 0.8000000 0.6000000 0.7000000 1.0000000
##
##                                    221       222       224       227       228
##   ’bad & very bad opinion’   0.0000000 0.0000000 0.7500000 0.2222222 0.2500000
##   ’very good & good opinion’ 1.0000000 1.0000000 0.2500000 0.7777778 0.7500000
##
```

```
##                                   229       232       235       236       237
##   'bad & very bad opinion'   0.0000000 0.8000000 0.5555556 1.0000000 0.2000000
##   'very good & good opinion' 1.0000000 0.2000000 0.4444444 0.0000000 0.8000000
##
##                                   239       242       243       245       246
##   'bad & very bad opinion'   0.0000000 0.4000000 0.1000000 0.0000000 0.8000000
##   'very good & good opinion' 1.0000000 0.6000000 0.9000000 1.0000000 0.2000000
##
##                                   247       250 252       254 255       256
##   'bad & very bad opinion'   0.3000000 0.4000000     0.5000000     1.0000000
##   'very good & good opinion' 0.7000000 0.6000000     0.5000000     0.0000000
##
##                                   257       258       262       263       264
##   'bad & very bad opinion'   0.0000000 0.0000000 0.6000000 0.0000000 0.2222222
##   'very good & good opinion' 1.0000000 1.0000000 0.4000000 1.0000000 0.7777778
##
##                                   266       267       271       275       276
##   'bad & very bad opinion'   0.5000000 0.0000000 0.5000000 0.7000000 0.2000000
##   'very good & good opinion' 0.5000000 1.0000000 0.5000000 0.3000000 0.8000000
##
##                                   277       280       281       282       284
##   'bad & very bad opinion'   0.4000000 0.2000000 0.0000000 0.7777778 0.7000000
##   'very good & good opinion' 0.6000000 0.8000000 1.0000000 0.2222222 0.3000000
##
##                                   285       286       287       288       289
##   'bad & very bad opinion'   0.9000000 0.2000000 0.1666667 0.4000000 0.2000000
##   'very good & good opinion' 0.1000000 0.8000000 0.8333333 0.6000000 0.8000000
##
##                                   292       296       298       300       301
##   'bad & very bad opinion'   0.0000000 0.0000000 0.4444444 0.1000000 0.1000000
##   'very good & good opinion' 1.0000000 1.0000000 0.5555556 0.9000000 0.9000000
##
##                                   303       306       307       308       317
##   'bad & very bad opinion'   0.4285714 0.2000000 0.3000000 0.2000000 0.1000000
##   'very good & good opinion' 0.5714286 0.8000000 0.7000000 0.8000000 0.9000000
##
##                                   319       321       324       325       326
##   'bad & very bad opinion'   0.7000000 0.2000000 0.1250000 0.0000000 0.1428571
##   'very good & good opinion' 0.3000000 0.8000000 0.8750000 1.0000000 0.8571429
##
##                                   328       329       330       334       336
##   'bad & very bad opinion'   0.0000000 0.1000000 0.0000000 0.0000000 0.1000000
##   'very good & good opinion' 1.0000000 0.9000000 1.0000000 1.0000000 0.9000000
##
##                                   340       341       342 343       344
##   'bad & very bad opinion'   0.1111111 0.7000000 0.1250000     0.3000000
##   'very good & good opinion' 0.8888889 0.3000000 0.8750000     0.7000000
##
##                                   346       348       350       351       355
##   'bad & very bad opinion'   0.5000000 0.5000000 0.2000000 0.0000000 0.5000000
##   'very good & good opinion' 0.5000000 0.5000000 0.8000000 1.0000000 0.5000000
##
##                                   356       357       358       360       361
##   'bad & very bad opinion'   0.0000000 0.0000000 0.4000000 0.3000000 0.5000000
```

```
##    'very good & good opinion' 1.0000000 1.0000000 0.6000000 0.7000000 0.5000000
##                                                                                
##                                     363       364 366       374       375
##    'bad & very bad opinion'   0.0000000 0.1000000     0.6000000 0.3333333
##    'very good & good opinion' 1.0000000 0.9000000     0.4000000 0.6666667
##                                                                                
##                                     377       384       389       390       391
##    'bad & very bad opinion'   0.2000000 0.2000000 0.2000000 0.5555556 0.3000000
##    'very good & good opinion' 0.8000000 0.8000000 0.8000000 0.4444444 0.7000000
##                                                                                
##                                     393       395       396       397       399
##    'bad & very bad opinion'   0.1000000 0.1000000 0.1111111 0.7777778 0.3000000
##    'very good & good opinion' 0.9000000 0.9000000 0.8888889 0.2222222 0.7000000
##                                                                                
##                                     400       403       408       409       410
##    'bad & very bad opinion'   0.3000000 0.1000000 0.0000000 0.0000000 0.0000000
##    'very good & good opinion' 0.7000000 0.9000000 1.0000000 1.0000000 1.0000000
##                                                                                
##                                     411       414       417       420 422
##    'bad & very bad opinion'   0.0000000 0.3750000 0.1000000 0.0000000    
##    'very good & good opinion' 1.0000000 0.6250000 0.9000000 1.0000000    
##                                                                                
##                                     424       428       430       431       433
##    'bad & very bad opinion'   0.2000000 0.0000000 0.2000000 1.0000000 0.1000000
##    'very good & good opinion' 0.8000000 1.0000000 0.8000000 0.0000000 0.9000000
##                                                                                
##                                     434       438 439       445       449
##    'bad & very bad opinion'   0.4000000 0.5000000     0.5000000 0.8000000
##    'very good & good opinion' 0.6000000 0.5000000     0.5000000 0.2000000
##                                                                                
##                                     450       452 456       459       461
##    'bad & very bad opinion'   0.3000000 0.3333333     0.2000000 0.3000000
##    'very good & good opinion' 0.7000000 0.6666667     0.8000000 0.7000000
##                                                                                
##                                     463       464       469       470       472
##    'bad & very bad opinion'   0.6000000 0.3000000 0.2857143 0.3000000 0.0000000
##    'very good & good opinion' 0.4000000 0.7000000 0.7142857 0.7000000 1.0000000
##                                                                                
##                                     473       476       477       479       482
##    'bad & very bad opinion'   0.5000000 0.1111111 0.2000000 0.2000000 0.6000000
##    'very good & good opinion' 0.5000000 0.8888889 0.8000000 0.8000000 0.4000000
##                                                                                
##                                     484       485       486       493       494
##    'bad & very bad opinion'   0.0000000 0.3000000 0.0000000 0.1000000 0.3000000
##    'very good & good opinion' 1.0000000 0.7000000 1.0000000 0.9000000 0.7000000
##                                                                                
##                                     500       502       503       505       506
##    'bad & very bad opinion'   0.1000000 0.1000000 0.2222222 0.6000000 0.3333333
##    'very good & good opinion' 0.9000000 0.9000000 0.7777778 0.4000000 0.6666667
##                                                                                
##                                     508       511       513       514       517
##    'bad & very bad opinion'   0.2000000 0.4444444 0.0000000 0.2000000 0.3000000
##    'very good & good opinion' 0.8000000 0.5555556 1.0000000 0.8000000 0.7000000
##
```

```
##                                    518       519       521       523       524
##   'bad & very bad opinion'   0.2000000 1.0000000 0.3333333 0.1000000 0.2000000
##   'very good & good opinion' 0.8000000 0.0000000 0.6666667 0.9000000 0.8000000
##
##                                    531       532       534       536       537
##   'bad & very bad opinion'   0.0000000 0.3000000 0.1000000 0.0000000 0.2000000
##   'very good & good opinion' 1.0000000 0.7000000 0.9000000 1.0000000 0.8000000
##
##                                    538       539       540       541       548
##   'bad & very bad opinion'   0.6000000 0.0000000 0.1111111 0.0000000 0.1000000
##   'very good & good opinion' 0.4000000 1.0000000 0.8888889 1.0000000 0.9000000
##
##                                    561       563       568       572       574
##   'bad & very bad opinion'   0.5000000 0.2000000 0.0000000 0.0000000 0.6000000
##   'very good & good opinion' 0.5000000 0.8000000 1.0000000 1.0000000 0.4000000
##
##                                    577       578       582       584       587
##   'bad & very bad opinion'   0.5000000 0.0000000 0.1000000 1.0000000 0.3000000
##   'very good & good opinion' 0.5000000 1.0000000 0.9000000 0.0000000 0.7000000
##
##                                    593       596       599       600       602
##   'bad & very bad opinion'   0.4000000 1.0000000 0.2222222 0.0000000 0.2000000
##   'very good & good opinion' 0.6000000 0.0000000 0.7777778 1.0000000 0.8000000
##
##                                    603       605       607       619       620
##   'bad & very bad opinion'   0.3000000 0.0000000 0.4000000 0.0000000 0.1111111
##   'very good & good opinion' 0.7000000 1.0000000 0.6000000 1.0000000 0.8888889
##
##                                    621       626       630       631       632
##   'bad & very bad opinion'   0.2000000 0.5714286 0.2222222 0.1000000 0.0000000
##   'very good & good opinion' 0.8000000 0.4285714 0.7777778 0.9000000 1.0000000
##
##                                    635       636       637       638       639
##   'bad & very bad opinion'   0.0000000 0.2500000 0.1428571 0.1000000 0.5000000
##   'very good & good opinion' 1.0000000 0.7500000 0.8571429 0.9000000 0.5000000
##
##                                    640       642       644       645       646
##   'bad & very bad opinion'   0.2000000 0.8888889 0.0000000 0.1428571 0.0000000
##   'very good & good opinion' 0.8000000 0.1111111 1.0000000 0.8571429 1.0000000
##
##                                    647       649 651       652       654
##   'bad & very bad opinion'   0.1666667 0.0000000     0.0000000 0.0000000
##   'very good & good opinion' 0.8333333 1.0000000     1.0000000 1.0000000
##
##                                    655       658       660       661       662
##   'bad & very bad opinion'   0.3000000 0.1111111 0.0000000 0.7777778 0.3000000
##   'very good & good opinion' 0.7000000 0.8888889 1.0000000 0.2222222 0.7000000
##
##                                    663       664       665       666       667
##   'bad & very bad opinion'   0.5555556 0.1428571 0.6000000 0.2222222 0.2000000
##   'very good & good opinion' 0.4444444 0.8571429 0.4000000 0.7777778 0.8000000
##
##                                    668       672       673       675       676
##   'bad & very bad opinion'   0.3000000 0.4000000 0.0000000 0.8571429 0.0000000
```

```
##   ’very good & good opinion’ 0.7000000 0.6000000 1.0000000 0.1428571 1.0000000
##
##                                    684       685       686       689       690
##   ’bad & very bad opinion’   0.0000000 0.1000000 0.2000000 0.8571429 0.0000000
##   ’very good & good opinion’ 1.0000000 0.9000000 0.8000000 0.1428571 1.0000000
##
##                                    691       692       693       695       699
##   ’bad & very bad opinion’   0.0000000 0.1000000 0.0000000 0.6000000 0.0000000
##   ’very good & good opinion’ 1.0000000 0.9000000 1.0000000 0.4000000 1.0000000
##
##                                    700       701       702       705       708
##   ’bad & very bad opinion’   0.4444444 0.4000000 0.3000000 0.0000000 1.0000000
##   ’very good & good opinion’ 0.5555556 0.6000000 0.7000000 1.0000000 0.0000000
##
##                                    709       710       711       712       713
##   ’bad & very bad opinion’   0.1000000 0.5000000 0.0000000 0.3000000 0.1000000
##   ’very good & good opinion’ 0.9000000 0.5000000 1.0000000 0.7000000 0.9000000
##
##                                    719       720 721       722       723
##   ’bad & very bad opinion’   0.1000000 0.0000000     0.1000000 0.1000000
##   ’very good & good opinion’ 0.9000000 1.0000000     0.9000000 0.9000000
##
##                                    727       730       731       732       739
##   ’bad & very bad opinion’   0.1000000 0.3000000 0.1000000 0.1000000 0.3000000
##   ’very good & good opinion’ 0.9000000 0.7000000 0.9000000 0.9000000 0.7000000
##
##                                    740       745       746       747       750
##   ’bad & very bad opinion’   0.7500000 0.2000000 0.3333333 0.0000000 0.1000000
##   ’very good & good opinion’ 0.2500000 0.8000000 0.6666667 1.0000000 0.9000000
##
##                                    751       753       754       755       756
##   ’bad & very bad opinion’   0.2000000 0.5000000 0.6000000 0.0000000 0.6000000
##   ’very good & good opinion’ 0.8000000 0.5000000 0.4000000 1.0000000 0.4000000
##
##                                    757       761       762       764       765
##   ’bad & very bad opinion’   0.0000000 0.3000000 0.7777778 0.3333333 0.0000000
##   ’very good & good opinion’ 1.0000000 0.7000000 0.2222222 0.6666667 1.0000000
##
##                                    767       768       769       773       774
##   ’bad & very bad opinion’   0.8000000 0.1000000 0.3750000 0.0000000 0.0000000
##   ’very good & good opinion’ 0.2000000 0.9000000 0.6250000 1.0000000 1.0000000
##
##                                    776       778       779       780       783
##   ’bad & very bad opinion’   0.1000000 0.0000000 0.1111111 0.0000000 0.1111111
##   ’very good & good opinion’ 0.9000000 1.0000000 0.8888889 1.0000000 0.8888889
##
##                                    784       788       789       790       791
##   ’bad & very bad opinion’   0.5000000 0.9000000 0.3750000 0.6000000 0.2222222
##   ’very good & good opinion’ 0.5000000 0.1000000 0.6250000 0.4000000 0.7777778
##
##                                    792       793       795       796       798
##   ’bad & very bad opinion’   0.5714286 0.0000000 0.2000000 0.0000000 0.4000000
##   ’very good & good opinion’ 0.4285714 1.0000000 0.8000000 1.0000000 0.6000000
##
```

```
##                                  799       802       806       808       809
##   'bad & very bad opinion'   0.7000000 0.0000000 0.0000000 0.3750000 0.3333333
##   'very good & good opinion' 0.3000000 1.0000000 1.0000000 0.6250000 0.6666667
##
##                                  814       817       819       820       821
##   'bad & very bad opinion'   0.0000000 0.6000000 0.1000000 0.8000000 0.0000000
##   'very good & good opinion' 1.0000000 0.4000000 0.9000000 0.2000000 1.0000000
##
##                                  822       823       824       827       828
##   'bad & very bad opinion'   0.4000000 0.1000000 0.5000000 0.0000000 0.0000000
##   'very good & good opinion' 0.6000000 0.9000000 0.5000000 1.0000000 1.0000000
##
##                                  831       834       837       838       842
##   'bad & very bad opinion'   0.0000000 0.7000000 0.0000000 0.0000000 0.2000000
##   'very good & good opinion' 1.0000000 0.3000000 1.0000000 1.0000000 0.8000000
##
##                                  843       844       847       849       853
##   'bad & very bad opinion'   0.0000000 0.0000000 0.3333333 0.4000000 0.0000000
##   'very good & good opinion' 1.0000000 1.0000000 0.6666667 0.6000000 1.0000000
##
##                                  854       857       859       860       864
##   'bad & very bad opinion'   0.0000000 0.0000000 0.7000000 0.2000000 0.2857143
##   'very good & good opinion' 1.0000000 1.0000000 0.3000000 0.8000000 0.7142857
##
##                                  866       869       870       872       875
##   'bad & very bad opinion'   0.0000000 0.4000000 0.5000000 0.3000000 0.8000000
##   'very good & good opinion' 1.0000000 0.6000000 0.5000000 0.7000000 0.2000000
##
##                                  878       880       882       883       884
##   'bad & very bad opinion'   0.9000000 0.1000000 0.5555556 0.2000000 0.0000000
##   'very good & good opinion' 0.1000000 0.9000000 0.4444444 0.8000000 1.0000000
##
##                                  885       886       887       889       893
##   'bad & very bad opinion'   1.0000000 0.0000000 0.2500000 0.0000000 0.0000000
##   'very good & good opinion' 0.0000000 1.0000000 0.7500000 1.0000000 1.0000000
##
##                                  894       896
##   'bad & very bad opinion'   0.3000000 0.5000000
##   'very good & good opinion' 0.7000000 0.5000000
```

```r
# alters' sex (proportions in each pers. netw.)
dat.factor$alter.sex <- factor(dat.factor$alter.sex,
             levels = c(0,1),
             labels = c("male", "female"))
prop.table(table(dat.factor$alter.sex, dat$ego_id), 2)
```

```
##
##            1   2   3   4   6   8   9  10  12  13  14  16  17  19  24  28  29
##   male   0.2 0.4 0.8 0.4 0.1 0.5 0.2 0.3 0.6 0.3 0.5 0.1 0.2 0.4 0.0 0.2 0.6
##   female 0.8 0.6 0.2 0.6 0.9 0.5 0.8 0.7 0.4 0.7 0.5 0.9 0.8 0.6 1.0 0.8 0.4
##
##           31  32  34  36  38  43  47  48  50  51  54  59  61  62  64  65  66
##   male   0.2 0.6 0.3 0.6 0.2 0.6 0.4 0.4 0.4 0.2 0.2 0.3 0.5 0.5 0.3 0.2 0.3
##   female 0.8 0.4 0.7 0.4 0.8 0.4 0.6 0.6 0.6 0.8 0.8 0.7 0.5 0.5 0.7 0.8 0.7
##
##           67  68  69  72  73  74  78  79  81  84  85  86  87  90  91  92  95
##   male   0.4 0.3 0.4 0.7 0.3 0.7 0.3 0.4 0.5 0.4 0.5 0.2 0.5 0.3 0.4 0.6 0.3
##   female 0.6 0.7 0.6 0.3 0.7 0.3 0.7 0.6 0.5 0.6 0.5 0.8 0.5 0.7 0.6 0.4 0.7
##
##           97  98  99 100 101 103 104 105 106 107 108 109 110 111 112 113 114
##   male   0.2 0.2 0.3 0.2 0.4 0.7 0.2 0.0 0.6 0.4 0.7 0.5 0.8 0.6 0.2 0.4 0.3
##   female 0.8 0.8 0.7 0.8 0.6 0.3 0.8 1.0 0.4 0.6 0.3 0.5 0.2 0.4 0.8 0.6 0.7
##
##          115 118 122 125 130 131 133 138 139 140 142 143 146 148 150 151 156
##   male   0.3 0.5 0.4 0.3 0.2 0.4 0.3 0.4 0.4 0.8 0.5 0.3 0.6 0.2 0.7 0.4 0.4
##   female 0.7 0.5 0.6 0.7 0.8 0.6 0.7 0.6 0.6 0.2 0.5 0.7 0.4 0.8 0.3 0.6 0.6
##
##          157 158 161 164 165 168 169 171 173 175 178 179 181 184 185 188 189
##   male   0.3 0.6 0.2 0.4 0.3 0.2 0.4 0.6 0.3 0.3 0.2 1.0 0.1 0.4 0.4 0.3 0.3
##   female 0.7 0.4 0.8 0.6 0.7 0.8 0.6 0.4 0.7 0.7 0.8 0.0 0.9 0.6 0.6 0.7 0.7
##
##          190 192 194 198 199 201 202 204 205 207 208 209 210 211 213 216 218
##   male   0.3 0.6 0.7 0.3 0.4 0.2 0.7 0.2 0.3 0.2 0.6 0.5 0.5 0.3 0.6 0.2 0.9
##   female 0.7 0.4 0.3 0.7 0.6 0.8 0.3 0.8 0.7 0.8 0.4 0.5 0.5 0.7 0.4 0.8 0.1
##
##          219 221 222 224 227 228 229 232 235 236 237 239 242 243 245 246 247
##   male   0.2 0.4 0.2 0.9 0.4 0.8 0.4 0.4 0.7 0.4 0.2 1.0 0.0 0.3 0.3 0.4 0.2
##   female 0.8 0.6 0.8 0.1 0.6 0.2 0.6 0.6 0.3 0.6 0.8 0.0 1.0 0.7 0.7 0.6 0.8
##
##          250 252 254 255 256 257 258 262 263 264 266 267 271 275 276 277 280
##   male   0.4 0.4 0.3 0.3 0.7 0.7 0.4 0.6 0.3 0.5 0.7 0.3 0.3 0.6 0.4 0.3 0.3
##   female 0.6 0.6 0.7 0.7 0.3 0.3 0.6 0.4 0.7 0.5 0.3 0.7 0.7 0.4 0.6 0.7 0.7
##
##          281 282 284 285 286 287 288 289 292 296 298 300 301 303 306 307 308
##   male   0.8 0.1 0.3 0.6 0.1 0.4 0.2 0.5 0.4 0.8 0.1 0.7 0.2 0.1 0.4 0.2 0.2
##   female 0.2 0.9 0.7 0.4 0.9 0.6 0.8 0.5 0.6 0.2 0.9 0.3 0.8 0.9 0.6 0.8 0.8
##
##          317 319 321 324 325 326 328 329 330 334 336 340 341 342 343 344 346
##   male   0.3 0.2 0.4 0.8 0.3 0.1 0.4 0.8 0.7 0.6 0.7 0.8 0.7 0.4 0.5 0.7 0.2
##   female 0.7 0.8 0.6 0.2 0.7 0.9 0.6 0.2 0.3 0.4 0.3 0.2 0.3 0.6 0.5 0.3 0.8
##
##          348 350 351 355 356 357 358 360 361 363 364 366 374 375 377 384 389
##   male   0.0 0.3 0.1 0.3 0.1 0.3 0.2 0.5 0.2 0.2 0.3 0.5 0.6 0.6 0.5 0.2 0.1
##   female 1.0 0.7 0.9 0.7 0.9 0.7 0.8 0.5 0.8 0.8 0.7 0.5 0.4 0.4 0.5 0.8 0.9
```

```
##
##          390 391 393 395 396 397 399 400 403 408 409 410 411 414 417 420 422
##   male   0.4 0.5 0.6 0.3 0.4 0.3 0.3 0.1 0.4 0.3 0.2 0.2 0.4 0.3 0.8 0.6 0.3
##   female 0.6 0.5 0.4 0.7 0.6 0.7 0.7 0.9 0.6 0.7 0.8 0.8 0.6 0.7 0.2 0.4 0.7
##
##          424 428 430 431 433 434 438 439 445 449 450 452 456 459 461 463 464
##   male   0.4 0.5 0.6 0.2 0.2 0.3 0.3 0.2 0.8 0.3 0.4 0.0 0.4 0.3 0.2 0.1 0.5
##   female 0.6 0.5 0.4 0.8 0.8 0.7 0.7 0.8 0.2 0.7 0.6 1.0 0.6 0.7 0.8 0.9 0.5
##
##          469 470 472 473 476 477 479 482 484 485 486 493 494 500 502 503 505
##   male   0.5 0.5 0.3 0.3 0.3 0.4 0.5 0.4 0.7 0.1 0.3 0.2 0.2 0.4 0.7 0.5 0.5
##   female 0.5 0.5 0.7 0.7 0.7 0.6 0.5 0.6 0.3 0.9 0.7 0.8 0.8 0.6 0.3 0.5 0.5
##
##          506 508 511 513 514 517 518 519 521 523 524 531 532 534 536 537 538
##   male   0.2 0.3 0.6 0.4 0.2 0.2 0.7 0.5 0.5 0.6 0.1 0.7 0.4 0.2 0.4 0.1 0.5
##   female 0.8 0.7 0.4 0.6 0.8 0.8 0.3 0.5 0.5 0.4 0.9 0.3 0.6 0.8 0.6 0.9 0.5
##
##          539 540 541 548 561 563 568 572 574 577 578 582 584 587 593 596 599
##   male   0.6 0.5 0.3 0.4 0.2 0.6 0.2 0.5 0.5 0.6 0.4 0.3 0.8 0.4 0.2 0.2 0.3
##   female 0.4 0.5 0.7 0.6 0.8 0.4 0.8 0.5 0.5 0.4 0.6 0.7 0.2 0.6 0.8 0.8 0.7
##
##          600 602 603 605 607 619 620 621 626 630 631 632 635 636 637 638 639
##   male   0.8 0.4 0.3 0.2 0.6 0.3 0.3 0.4 0.2 0.1 0.3 0.3 0.3 0.2 0.2 0.1 0.3
##   female 0.2 0.6 0.7 0.8 0.4 0.7 0.7 0.6 0.8 0.9 0.7 0.7 0.7 0.8 0.8 0.9 0.7
##
##          640 642 644 645 646 647 649 651 652 654 655 658 660 661 662 663 664
##   male   0.6 0.6 0.4 0.3 0.3 0.4 0.1 0.7 0.4 0.4 0.8 0.6 0.3 0.2 0.4 0.2 0.2
##   female 0.4 0.4 0.6 0.7 0.7 0.6 0.9 0.3 0.6 0.6 0.2 0.4 0.7 0.8 0.6 0.8 0.8
##
##          665 666 667 668 672 673 675 676 684 685 686 689 690 691 692 693 695
##   male   0.2 0.2 0.4 0.2 0.3 0.3 0.2 0.2 0.1 0.4 0.8 0.1 0.7 0.4 0.4 0.4 0.3
##   female 0.8 0.8 0.6 0.8 0.7 0.7 0.8 0.8 0.9 0.6 0.2 0.9 0.3 0.6 0.6 0.6 0.7
##
##          699 700 701 702 705 708 709 710 711 712 713 719 720 721 722 723 727
##   male   0.3 0.1 0.6 0.3 0.7 0.2 0.2 0.3 0.6 0.7 0.2 0.3 0.4 0.2 0.5 0.2 0.5
##   female 0.7 0.9 0.4 0.7 0.3 0.8 0.8 0.7 0.4 0.3 0.8 0.7 0.6 0.8 0.5 0.8 0.5
##
##          730 731 732 739 740 745 746 747 750 751 753 754 755 756 757 761 762
##   male   0.2 0.3 0.6 0.4 0.3 0.3 0.2 0.1 0.2 0.3 0.3 0.1 0.7 0.5 0.5 0.3 0.2
##   female 0.8 0.7 0.4 0.6 0.7 0.7 0.8 0.9 0.8 0.7 0.7 0.9 0.3 0.5 0.5 0.7 0.8
##
##          764 765 767 768 769 773 774 776 778 779 780 783 784 788 789 790 791
##   male   0.2 0.3 0.4 0.4 0.5 0.2 0.7 0.2 0.1 0.8 0.5 0.1 0.6 0.6 0.2 0.3 0.7
##   female 0.8 0.7 0.6 0.6 0.5 0.8 0.3 0.8 0.9 0.2 0.5 0.9 0.4 0.4 0.8 0.7 0.3
##
##          792 793 795 796 798 799 802 806 808 809 814 817 819 820 821 822 823
##   male   0.4 0.5 0.2 0.7 0.6 0.2 0.6 0.8 0.7 0.6 0.1 0.5 0.6 0.5 0.4 0.2 0.2
##   female 0.6 0.5 0.8 0.3 0.4 0.8 0.4 0.2 0.3 0.4 0.9 0.5 0.4 0.5 0.6 0.8 0.8
##
##          824 827 828 831 834 837 838 842 843 844 847 849 853 854 857 859 860
##   male   0.4 0.2 0.5 0.5 0.3 0.3 0.3 0.4 0.2 0.5 0.2 0.4 0.5 0.2 0.5 0.3 0.5
##   female 0.6 0.8 0.5 0.5 0.7 0.7 0.7 0.6 0.8 0.5 0.8 0.6 0.5 0.8 0.5 0.7 0.5
##
##          864 866 869 870 872 875 878 880 882 883 884 885 886 887 889 893 894
```

```
##   male   0.3 0.3 0.7 0.3 0.4 0.4 0.2 0.2 0.4 0.2 0.3 0.4 0.7 0.5 0.4 0.8 0.2
##   female 0.7 0.7 0.3 0.7 0.6 0.6 0.8 0.8 0.6 0.8 0.7 0.6 0.3 0.5 0.6 0.2 0.8
##
##          896
##   male   0.5
##   female 0.5
```

```r
# alters' education (proportions in each pers. netw.)
dat.factor$alter.edu <- factor(dat.factor$alter.edu,
                               levels = c(0,1),
                               labels = c("no higher edu.",
                                          "yes higher edu."))
prop.table(table(dat.factor$alter.edu, dat$ego_id), 2)
```

```
##
##                       1   2   3   4   6   8   9  10  12  13  14  16  17  19  24
##   no higher edu.  0.4 0.3 0.8 1.0 0.4 0.7 0.4 0.3 0.9 0.3 0.5 0.6 0.4 0.6 0.3
##   yes higher edu. 0.6 0.7 0.2 0.0 0.6 0.3 0.6 0.7 0.1 0.7 0.5 0.4 0.6 0.4 0.7
##
##                      28  29  31  32  34  36  38  43  47  48  50  51  54  59  61
##   no higher edu.  0.7 0.3 0.2 0.7 0.7 0.6 0.3 0.5 0.5 0.9 0.0 0.6 0.3 0.5 0.6
##   yes higher edu. 0.3 0.7 0.8 0.3 0.3 0.4 0.7 0.5 0.5 0.1 1.0 0.4 0.7 0.5 0.4
##
##                      62  64  65  66  67  68  69  72  73  74  78  79  81  84  85
##   no higher edu.  0.5 0.4 0.1 0.6 0.5 0.3 0.4 1.0 0.5 0.8 0.5 0.7 0.8 0.9 0.4
##   yes higher edu. 0.5 0.6 0.9 0.4 0.5 0.7 0.6 0.0 0.5 0.2 0.5 0.3 0.2 0.1 0.6
##
##                      86  87  90  91  92  95  97  98  99 100 101 103 104 105 106
##   no higher edu.  0.7 0.4 0.2 0.9 0.6 0.2 1.0 0.6 0.9 0.6 0.7 0.3 0.4 0.7 0.9
##   yes higher edu. 0.3 0.6 0.8 0.1 0.4 0.8 0.0 0.4 0.1 0.4 0.3 0.7 0.6 0.3 0.1
##
##                     107 108 109 110 111 112 113 114 115 118 122 125 130 131 133
##   no higher edu.  0.5 0.3 0.2 0.9 0.8 0.7 0.2 1.0 0.8 0.4 0.9 0.3 0.8 0.7 0.0
##   yes higher edu. 0.5 0.7 0.8 0.1 0.2 0.3 0.8 0.0 0.2 0.6 0.1 0.7 0.2 0.3 1.0
##
##                     138 139 140 142 143 146 148 150 151 156 157 158 161 164 165
##   no higher edu.  0.0 0.7 0.3 0.5 0.2 0.0 0.7 0.2 0.3 0.2 0.5 0.3 0.3 0.1 0.3
##   yes higher edu. 1.0 0.3 0.7 0.5 0.8 1.0 0.3 0.8 0.7 0.8 0.5 0.7 0.7 0.9 0.7
##
##                     168 169 171 173 175 178 179 181 184 185 188 189 190 192 194
##   no higher edu.  0.7 0.4 0.8 0.6 0.3 0.4 0.9 0.5 0.3 1.0 0.7 0.3 0.5 0.4 0.7
##   yes higher edu. 0.3 0.6 0.2 0.4 0.7 0.6 0.1 0.5 0.7 0.0 0.3 0.7 0.5 0.6 0.3
##
##                     198 199 201 202 204 205 207 208 209 210 211 213 216 218 219
##   no higher edu.  0.2 0.8 0.4 0.6 0.0 0.9 0.3 0.0 1.0 0.8 0.7 0.8 0.5 0.0 0.5
##   yes higher edu. 0.8 0.2 0.6 0.4 1.0 0.1 0.7 1.0 0.0 0.2 0.3 0.2 0.5 1.0 0.5
##
##                     221 222 224 227 228 229 232 235 236 237 239 242 243 245 246
##   no higher edu.  0.2 0.4 0.9 0.5 0.4 0.9 0.8 0.4 0.9 0.3 0.2 0.4 0.2 0.5 1.0
##   yes higher edu. 0.8 0.6 0.1 0.5 0.6 0.1 0.2 0.6 0.1 0.7 0.8 0.6 0.8 0.5 0.0
##
##                     247 250 252 254 255 256 257 258 262 263 264 266 267 271 275
##   no higher edu.  0.7 0.8 0.4 0.4 0.9 0.3 0.9 0.2 0.2 0.7 0.1 0.3 0.9 0.3 0.5
##   yes higher edu. 0.3 0.2 0.6 0.6 0.1 0.7 0.1 0.8 0.8 0.3 0.9 0.7 0.1 0.7 0.5
##
##                     276 277 280 281 282 284 285 286 287 288 289 292 296 298 300
##   no higher edu.  0.6 0.5 0.7 0.2 0.6 0.5 0.9 0.2 0.7 0.7 0.9 0.2 0.7 0.1 0.8
##   yes higher edu. 0.4 0.5 0.3 0.8 0.4 0.5 0.1 0.8 0.3 0.3 0.1 0.8 0.3 0.9 0.2
##
##                     301 303 306 307 308 317 319 321 324 325 326 328 329 330 334
##   no higher edu.  0.2 0.6 0.8 0.5 0.1 1.0 0.3 0.3 0.8 0.4 0.7 0.4 0.4 0.9 0.5
```

```
##   yes higher edu. 0.8 0.4 0.2 0.5 0.9 0.0 0.7 0.7 0.2 0.6 0.3 0.6 0.6 0.1 0.5
##
##                   336 340 341 342 343 344 346 348 350 351 355 356 357 358 360
##   no higher edu.  0.7 0.4 0.6 0.2 0.9 0.7 0.4 0.5 0.9 0.7 0.5 0.1 1.0 0.2 0.0
##   yes higher edu. 0.3 0.6 0.4 0.8 0.1 0.3 0.6 0.5 0.1 0.3 0.5 0.9 0.0 0.8 1.0
##
##                   361 363 364 366 374 375 377 384 389 390 391 393 395 396 397
##   no higher edu.  0.7 0.4 0.4 0.7 0.8 0.5 0.7 0.3 0.2 0.6 0.0 0.8 0.1 0.7 0.7
##   yes higher edu. 0.3 0.6 0.6 0.3 0.2 0.5 0.3 0.7 0.8 0.4 1.0 0.2 0.9 0.3 0.3
##
##                   399 400 403 408 409 410 411 414 417 420 422 424 428 430 431
##   no higher edu.  0.4 0.3 0.5 0.3 0.9 0.0 0.2 0.8 0.9 0.5 0.8 0.2 0.9 0.3 0.5
##   yes higher edu. 0.6 0.7 0.5 0.7 0.1 1.0 0.8 0.2 0.1 0.5 0.2 0.8 0.1 0.7 0.5
##
##                   433 434 438 439 445 449 450 452 456 459 461 463 464 469 470
##   no higher edu.  0.5 0.4 0.4 0.4 0.7 0.4 0.5 0.4 0.8 0.5 0.1 0.6 0.3 0.1 0.4
##   yes higher edu. 0.5 0.6 0.6 0.6 0.3 0.6 0.5 0.6 0.2 0.5 0.9 0.4 0.7 0.9 0.6
##
##                   472 473 476 477 479 482 484 485 486 493 494 500 502 503 505
##   no higher edu.  0.1 0.7 0.1 0.6 0.4 0.3 0.2 0.8 0.6 1.0 0.2 0.6 0.5 0.5 0.8
##   yes higher edu. 0.9 0.3 0.9 0.4 0.6 0.7 0.8 0.2 0.4 0.0 0.8 0.4 0.5 0.5 0.2
##
##                   506 508 511 513 514 517 518 519 521 523 524 531 532 534 536
##   no higher edu.  0.6 0.5 0.1 1.0 0.6 0.3 0.6 0.7 0.9 0.4 0.4 0.3 0.5 0.5 0.5
##   yes higher edu. 0.4 0.5 0.9 0.0 0.4 0.7 0.4 0.3 0.1 0.6 0.6 0.7 0.5 0.5 0.5
##
##                   537 538 539 540 541 548 561 563 568 572 574 577 578 582 584
##   no higher edu.  0.5 0.2 0.5 0.2 0.3 0.0 0.1 0.2 0.2 0.2 0.3 0.0 0.0 0.2 1.0
##   yes higher edu. 0.5 0.8 0.5 0.8 0.7 1.0 0.9 0.8 0.8 0.8 0.7 1.0 1.0 0.8 0.0
##
##                   587 593 596 599 600 602 603 605 607 619 620 621 626 630 631
##   no higher edu.  0.3 0.3 0.5 0.3 0.0 0.4 0.2 0.1 0.9 0.2 0.3 0.9 0.3 0.8 0.5
##   yes higher edu. 0.7 0.7 0.5 0.7 1.0 0.6 0.8 0.9 0.1 0.8 0.7 0.1 0.7 0.2 0.5
##
##                   632 635 636 637 638 639 640 642 644 645 646 647 649 651 652
##   no higher edu.  0.2 0.7 0.6 0.8 0.4 0.9 0.5 0.7 0.2 1.0 0.6 0.8 0.7 0.8 0.3
##   yes higher edu. 0.8 0.3 0.4 0.2 0.6 0.1 0.5 0.3 0.8 0.0 0.4 0.2 0.3 0.2 0.7
##
##                   654 655 658 660 661 662 663 664 665 666 667 668 672 673 675
##   no higher edu.  0.2 0.6 0.3 0.2 0.8 0.7 0.7 0.6 0.8 0.5 0.5 0.7 1.0 0.3 0.6
##   yes higher edu. 0.8 0.4 0.7 0.8 0.2 0.3 0.3 0.4 0.2 0.5 0.5 0.3 0.0 0.7 0.4
##
##                   676 684 685 686 689 690 691 692 693 695 699 700 701 702 705
##   no higher edu.  0.9 0.5 0.1 0.2 0.6 0.2 0.9 0.8 0.9 0.8 0.5 0.2 0.3 0.1 0.9
##   yes higher edu. 0.1 0.5 0.9 0.8 0.4 0.8 0.1 0.2 0.1 0.2 0.5 0.8 0.7 0.9 0.1
##
##                   708 709 710 711 712 713 719 720 721 722 723 727 730 731 732
##   no higher edu.  0.8 0.6 0.3 0.3 0.4 0.2 0.7 0.5 0.2 0.6 0.8 0.1 0.2 0.6 0.3
##   yes higher edu. 0.2 0.4 0.7 0.7 0.6 0.8 0.3 0.5 0.8 0.4 0.2 0.9 0.8 0.4 0.7
##
##                   739 740 745 746 747 750 751 753 754 755 756 757 761 762 764
##   no higher edu.  0.5 1.0 0.7 0.2 0.7 0.1 0.7 0.3 0.3 1.0 0.7 0.1 0.3 0.5 0.3
##   yes higher edu. 0.5 0.0 0.3 0.8 0.3 0.9 0.3 0.7 0.7 0.0 0.3 0.9 0.7 0.5 0.7
##
```

```
##                    765 767 768 769 773 774 776 778 779 780 783 784 788 789 790
##   no higher edu.  0.2 0.9 0.5 0.4 0.2 0.0 0.2 0.5 0.5 0.4 0.2 0.7 0.2 0.7 0.9
##   yes higher edu. 0.8 0.1 0.5 0.6 0.8 1.0 0.8 0.5 0.5 0.6 0.8 0.3 0.8 0.3 0.1
##
##                    791 792 793 795 796 798 799 802 806 808 809 814 817 819 820
##   no higher edu.  1.0 0.1 0.4 0.4 0.3 0.6 0.4 0.0 0.6 0.4 0.7 0.2 0.2 0.5 0.6
##   yes higher edu. 0.0 0.9 0.6 0.6 0.7 0.4 0.6 1.0 0.4 0.6 0.3 0.8 0.8 0.5 0.4
##
##                    821 822 823 824 827 828 831 834 837 838 842 843 844 847 849
##   no higher edu.  0.1 0.6 0.4 0.8 0.4 0.0 0.4 0.6 0.1 0.8 0.2 0.6 0.6 0.5 0.8
##   yes higher edu. 0.9 0.4 0.6 0.2 0.6 1.0 0.6 0.4 0.9 0.2 0.8 0.4 0.4 0.5 0.2
##
##                    853 854 857 859 860 864 866 869 870 872 875 878 880 882 883
##   no higher edu.  0.1 0.7 0.5 0.4 0.5 0.9 0.5 0.3 0.8 0.5 1.0 0.2 0.8 0.7 0.5
##   yes higher edu. 0.9 0.3 0.5 0.6 0.5 0.1 0.5 0.7 0.2 0.5 0.0 0.8 0.2 0.3 0.5
##
##                    884 885 886 887 889 893 894 896
##   no higher edu.  0.4 0.8 0.8 0.1 0.6 0.2 0.8 0.6
##   yes higher edu. 0.6 0.2 0.2 0.9 0.4 0.8 0.2 0.4
```

Visualizations Information in the visualizations is computed by personal networks as grouping variable

```
myData1 <- alter.df2
```

```
myData <- myData1 %>%
  group_by(ego_id) %>%
  summarise(
    # proportion of alters with 'very good & good' opinions about COVID-19 vaccination,
    # i.e., 'pro-vaccination'
    prop.pro.covid = mean(alter.covid, na.rm = TRUE),

    # proportion of female alters in each personal network
    prop.females = mean(alter.sex, na.rm = TRUE),

    # proportion of alters with higher education studies in each personal network
    prop.high.edu = mean(alter.edu, na.rm = TRUE),

    # mean age of alters in each personal network
    a.mean.age = mean(alter.age, na.rm = TRUE),

    # mean of ego-alter tie duration in each personal network
    ea.mean.duration = mean(ego.alter.duration, na.rm = TRUE),

    # mean of alters' betweenness in each personal netw.
    a.mean.betw = mean(alter.betw, na.rm = TRUE),

    # mean of alters' degree in each pers. netw.
    mean.a.degree = mean(alter.deg, na.rm = TRUE),

    # mean of ties to pro-vaccination peers in each pers. netw.
    mean.ties.to.pro.vacc = mean(ties.to.pro.vacc, na.rm = TRUE),

    # mean of ties to anti-vaccination peers in each pers. netw.
    mean.ties.to.anti.vacc = mean(ties.to.anti.vacc, na.rm = TRUE)
```

```
  )
```

```
descr(myData$prop.pro.covid) # variables by ego.id
```

```
## Descriptive Statistics
## myData$prop.pro.covid
## N: 443
##
##                        prop.pro.covid
## ----------------- ----------------
##              Mean             0.73
##           Std.Dev             0.27
##               Min             0.00
##                Q1             0.60
##            Median             0.80
##                Q3             1.00
##               Max             1.00
##               MAD             0.30
##               IQR             0.40
##                CV             0.37
##          Skewness            -0.97
##       SE.Skewness             0.12
##          Kurtosis             0.12
##           N.Valid           432.00
##         Pct.Valid            97.52
```

```
par(mar = c(4, 2, 2, 2))
par(mfrow=c(3,4))

# 'pro-vaccination alters' (mean of 'alter.covid' by pers. netw.)
hist(myData$prop.pro.covid,
     col="snow2",
     border = "snow3",
     probability = TRUE,
     xlab = "alters pro vaccination (%)",
     main = "",
     xlim = c(0.00, 1.00),
     breaks = 9)
lines(density(myData$prop.pro.covid, na.rm = TRUE),
      lwd=2,
      col = "firebrick1")
abline(v = median(myData$prop.pro.covid, na.rm = TRUE),
       col = "slateblue4",
       lwd = 2,
       lty= 1)

# 'female-alters' proportion (mean of 'alter.sex' by pers. netw.)
hist(myData$prop.females,
     col="snow2",
     border = "snow3",
     probability = TRUE,
     xlab = "alters female (%)",
     main = "",
     breaks = 10)
```

```
lines(density(myData$prop.females, na.rm = TRUE),
      lwd=2,
      col = "firebrick1")
abline(v = median(myData$prop.females, na.rm = TRUE),
       col = "slateblue4",
       lwd = 2,
       lty= 1)

# higher education alters proportion (mean of 'alter.edu' by pers. network)
hist(myData$prop.high.edu,
     col="snow2",
     border = "snow3",
     probability = TRUE,
     xlab = "alters higher edu (%)",
     main = "",
     breaks = 9)
lines(density(myData$prop.high.edu, na.rm = TRUE),
      lwd=2,
      col = "firebrick1")
abline(v = median(myData$prop.high.edu, na.rm = TRUE),
       col = "slateblue4",
       lwd = 2,
       lty= 1)

# mean age of alters (mean of "alter.age" by pers. netw.)
hist(myData$a.mean.age,
     col="snow2",
     border = "snow3",
     probability = TRUE,
     xlab = "alters: mean age",
     main = "",
     breaks = 60,
     xlim = c(18, 80))
lines(density(myData$a.mean.age, na.rm = TRUE),
      lwd=2,
      col = "firebrick1")
abline(v = median(myData$a.mean.age, na.rm = TRUE),
       col = "slateblue4",
       lwd = 2,
       lty= 1)

# mean of ego-alter tie duration (mean of 'ego.alter.duration'by pers. netw.)
hist(myData$ea.mean.duration,
     col="snow2",
     border = "snow3",
     probability = TRUE,
     xlab = "ego-alter mean duration",
     main = "",
     breaks = 60)
lines(density(myData$ea.mean.duration, na.rm = TRUE),
      lwd=2,
      col = "firebrick1")
abline(v = median(myData$ea.mean.duration, na.rm = TRUE),
```

```
       col = "slateblue4",
       lwd = 2,
       lty= 1)

# alters: betweenness (mean) (mean of "alter.betw" in each pers. netw.)
hist(myData$a.mean.betw,
     col="snow2",
     border = "snow3",
     probability = TRUE,
     xlab = "alters betweenness (mean)",
     main = "",
     breaks = 9)
lines(density(myData$a.mean.betw, na.rm = TRUE),
      lwd=2,
      col = "firebrick1")
abline(v = median(myData$a.mean.betw, na.rm = TRUE),
       col = "slateblue4",
       lwd = 2,
       lty= 1)

# ties to pro-vacc peers (mean) (mean of 'ties.to.pro.vacc' by pers. netw.)
hist(myData$mean.ties.to.pro.vacc,
     col="snow2",
     border = "snow3",
     probability = TRUE,
     xlab = "ties to pro vacc peers (mean)",
     main = "",
     xlim = c(0, 9),
     breaks = 9)
lines(density(myData$mean.ties.to.pro.vacc, na.rm = TRUE),
      lwd=2,
      col = "firebrick1")
abline(v = median(myData$mean.ties.to.pro.vacc, na.rm = TRUE),
       col = "slateblue4",
       lwd = 2,
       lty= 1)

# ties to anti-vacc peers (mean) (mean of 'ties.to.anti.vacc' by pers. netw.)
hist(myData$mean.ties.to.anti.vacc,
     col="snow2",
     border = "snow3",
     probability = TRUE,
     xlab = "ties to anti vacc peers (mean)",
     main = "",
     xlim = c(0, 9),
     breaks = 9)
lines(density(myData$mean.ties.to.anti.vacc, na.rm = TRUE),
      lwd=2,
      col = "firebrick1")
abline(v = median(myData$mean.ties.to.anti.vacc, na.rm = TRUE),
       col = "slateblue4",
       lwd = 2,
       lty= 1)
```

```
# 'components' in each pers. netw.
hist(myData1$comp,
     col="snow2",
     border = "snow3",
     probability = TRUE,
     xlab = "components",
     main = "",
     breaks = 9,
     xlim = c(0, 10))
lines(density(myData1$comp, na.rm = TRUE),
      lwd=2,
      col = "firebrick1")
abline(v = median(myData1$comp, na.rm = TRUE),
       col = "slateblue4",
       lwd = 2,
       lty= 1)

# network 'density' in each pers. netw.
hist(myData1$dens,
     col="snow2",
     border = "snow3",
     probability = TRUE,
     xlab = "network density",
     main = "",
     breaks = 9)
lines(density(myData1$dens, na.rm = TRUE),
      lwd=2,
      col = "firebrick1")
abline(v = median(myData1$dens, na.rm = TRUE),
       col = "slateblue4",
       lwd = 2,
       lty= 1)

# 'network centralization' in pers. networks
hist(myData1$centraliz,
     col="snow2",
     border = "snow3",
     probability = TRUE,
     xlab = "centralization",
     main = "",
     xlim = c(0.00, 0.80),
     breaks = 9)
lines(density(myData1$centraliz, na.rm = TRUE),
      lwd=2,
      col = "firebrick1")
abline(v = median(myData1$centraliz, na.rm = TRUE),
       col = "slateblue4",
       lwd = 2,
       lty= 1)

# mean of alters' degree (mean of "alter.deg" in each pers. netw.)
hist(myData1$alter.deg,
     col="snow2",
```

```
      border = "snow3",
      probability = TRUE,
      xlab = "alters: mean degree",
      main = "",
      xlim = c(0, 10),
      breaks = 9)
lines(density(myData1$alter.deg, na.rm = TRUE),
       lwd=2,
       col = "firebrick1")
abline(v = median(myData1$alter.deg, na.rm = TRUE),
        col = "slateblue4",
        lwd = 2,
        lty= 1)
```

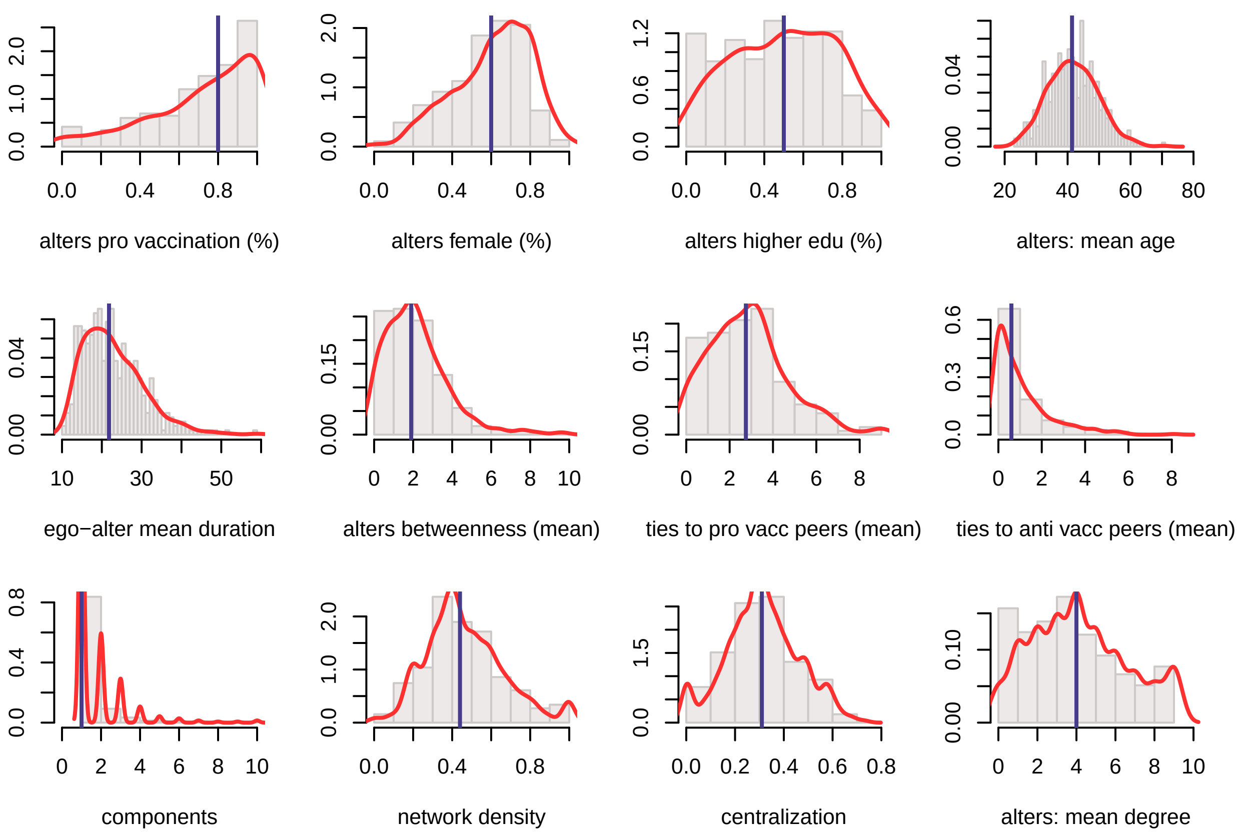

Fig. 1: Density-histogram plots of the descriptive statistics computed for each personal network. (The blue line marks the median scores).

```
dev.off()
```

Numeric We group by ego's id (personal networks)

```
dat <- alter.df2

dat.num <- dat %>%
  select(alter.age, alter.betw,
         ego.alter.duration, prop.pro.vacc.ties, ego_id)

dat.num$ego_id <- as.character(dat.num$ego_id)
```

```
dat.num %>%
  group_by(ego_id)%>%
  summarise(across(
    .cols = where(is.numeric),
    .fns = mean,
    na.rm = TRUE
  ))
```

```
## # A tibble: 443 x 5
##    ego_id alter.age alter.betw ego.alter.duration prop.pro.vacc.ties
##    <chr>      <dbl>      <dbl>              <dbl>              <dbl>
##  1 1           34.4       4.8                13.9              0.972
##  2 10          34         0                  11                1
##  3 100         36.8       1.10               15.6              0.128
##  4 101         45.3       1.9                37.5              0.699
##  5 103         63.2       3.2                37.9              0.917
##  6 104         47.8       1.6                24.8              0.207
##  7 105         38.6       5.00               16.8              0.883
##  8 106         37.8       2.40               20.5              0.223
##  9 107         32         1.7                15.6              0.65
## 10 108         46.9       1.90               28.6              0.410
## # i 433 more rows
```

The code snippet should be run on local machine to inspect all values. Given space limitation, we print here only a part of the output.

2.2 Alter analysis

```r
library(lme4)
```

2.2.1 The models

```
## Warning: package 'lme4' was built under R version 4.2.3

## Warning: package 'Matrix' was built under R version 4.2.3
```

```r
# Multi-level logistic regression model
m0 <- glmer(alter.covid # the dependent variable
            ~ 1 # the intercept term (the only predictor)
            + (1 | ego_id) # the intercept can vary by ego (pers. netw.)
            , na.omit(alter.df3) # use only complete observations
            , set.seed(1234)
            , family = "binomial"
            , nAGQ = 100
            , control=glmerControl(optimizer = "bobyqa")) # use R's optimization routine

summary(m0) # summary of the model
```

Model 0 ('null model')

```
## Generalized linear mixed model fit by maximum likelihood (Adaptive
##   Gauss-Hermite Quadrature, nAGQ = 100) [glmerMod]
##  Family: binomial  ( logit )
## Formula: alter.covid ~ 1 + (1 | ego_id)
##    Data: na.omit(alter.df3)
## Control: glmerControl(optimizer = "bobyqa")
##
##      AIC      BIC   logLik deviance df.resid
##   3616.3   3628.7  -1806.1   3612.3     3586
##
## Scaled residuals:
##     Min      1Q  Median      3Q     Max
## -2.6833 -0.4049  0.2633  0.4959  1.8938
##
## Random effects:
##  Groups Name        Variance Std.Dev.
##  ego_id (Intercept) 2.323    1.524
## Number of obs: 3588, groups:  ego_id, 401
##
## Fixed effects:
##             Estimate Std. Error z value Pr(>|z|)
## (Intercept)  1.46425    0.09596   15.26   <2e-16 ***
## ---
## Signif. codes:  0 '***' 0.001 '**' 0.01 '*' 0.05 '.' 0.1 ' ' 1
```

```r
# table of estimates with 95% CI
se0 <- sqrt(diag(vcov(m0)))
(tab0 <- cbind(Est = fixef(m0),
               LL = fixef(m0) - 1.96 * se0,
               UL = fixef(m0) + 1.96 * se0))
```

```
##                  Est       LL       UL
## (Intercept) 1.464246 1.276155 1.652337
```

```r
round((exp(tab0)),2) # we exponentiate the estimates (Odds ratios)
```

```
##              Est   LL   UL
## (Intercept) 4.32 3.58 5.22
```

```r
round((tab0),2) # raw estimates
```

```
##              Est   LL   UL
## (Intercept) 1.46 1.28 1.65
```

```r
library(performance)
```

```r
ICC0 <- performance::icc(m0, by_group = TRUE)
ICC0 # estimate the ICC
```

```
## # ICC by Group
##
## Group  |   ICC
## ---------------
## ego_id | 0.414
```

```r
# standard logistic regression model
m0.glm <- glm(alter.covid # the dependent variable
              ~ 1 # the intercept term (the only predictor)
              + prop.vacc.ex.alter # proportion of alters that are pro-vaccination (minus alter of refe
              , na.omit(alter.df3) # use only complete observations
              , set.seed(1234)
              , family = "binomial"
)

summary(m0.glm) # summary of the model
```

```
##
## Call:
## glm(formula = alter.covid ~ 1 + prop.vacc.ex.alter, family = "binomial",
##     data = na.omit(alter.df3), weights = set.seed(1234))
##
## Deviance Residuals:
##     Min       1Q   Median       3Q      Max
## -2.2208  -0.6563   0.5147   0.7549   1.8118
##
## Coefficients:
##                     Estimate Std. Error z value Pr(>|z|)
## (Intercept)          1.26950    0.04496   28.23   <2e-16 ***
## prop.vacc.ex.alter  1.00975    0.04351   23.21   <2e-16 ***
## ---
## Signif. codes:  0 '***' 0.001 '**' 0.01 '*' 0.05 '.' 0.1 ' ' 1
##
```

```
## (Dispersion parameter for binomial family taken to be 1)
##
##     Null deviance: 4076.5  on 3587  degrees of freedom
## Residual deviance: 3425.0  on 3586  degrees of freedom
## AIC: 3429
##
## Number of Fisher Scoring iterations: 4
```

```r
# Multi-level logistic regression model
m1 <- glmer(alter.covid ~
            # alters' attributes (level 1 predictors)
            alter.sex
            + alter.edu
            + alter.age
            # egos' attributes (level 2 predictors)
            + ego.sex
            + ego.edu
            + ego.income.cat
            + ego.age
            + ego.covid
            + (1 | ego_id) # the intercept can vary by ego (pers. netw.)
            , family = "binomial"
            , na.omit(alter.df3) # use only complete observations
            , set.seed(1234)
            , nAGQ = 100
            , control=glmerControl(optimizer = "bobyqa")) # use R's optimization routine

summary(m1)
```

Model 1 ('attributes model')

```
## Generalized linear mixed model fit by maximum likelihood (Adaptive
##   Gauss-Hermite Quadrature, nAGQ = 100) [glmerMod]
##  Family: binomial  ( logit )
## Formula: alter.covid ~ alter.sex + alter.edu + alter.age + ego.sex + ego.edu +
##     ego.income.cat + ego.age + ego.covid + (1 | ego_id)
##    Data: na.omit(alter.df3)
## Control: glmerControl(optimizer = "bobyqa")
##
##      AIC      BIC   logLik deviance df.resid
##   3462.6   3524.5  -1721.3   3442.6     3578
##
## Scaled residuals:
##     Min      1Q  Median      3Q     Max
## -3.4786 -0.4060  0.3115  0.4734  2.1719
##
## Random effects:
##  Groups Name        Variance Std.Dev.
##  ego_id (Intercept) 1.153    1.074
## Number of obs: 3588, groups:  ego_id, 401
##
## Fixed effects:
##                  Estimate Std. Error z value Pr(>|z|)
## (Intercept)    -0.68771    0.24771  -2.776   0.0055 **
## alter.sex       0.07704    0.09869   0.781   0.4350
## alter.edu       0.45801    0.10603   4.320 1.56e-05 ***
## alter.age       0.15125    0.05424   2.789   0.0053 **
## ego.sex         0.07806    0.17647   0.442   0.6582
## ego.edu        -0.28242    0.16886  -1.673   0.0944 .
## ego.income.cat  0.18297    0.10071   1.817   0.0692 .
## ego.age         0.04584    0.07890   0.581   0.5612
```

```
## ego.covid       2.13808    0.17445  12.256  < 2e-16 ***
## ---
## Signif. codes:  0 '***' 0.001 '**' 0.01 '*' 0.05 '.' 0.1 ' ' 1
##
## Correlation of Fixed Effects:
##             (Intr) altr.s altr.d altr.g ego.sx ego.ed eg.nc. ego.ag
## alter.sex   -0.142
## alter.edu   -0.086 -0.092
## alter.age   -0.018 -0.056  0.311
## ego.sex     -0.552 -0.168  0.016  0.001
## ego.edu     -0.215 -0.013 -0.166 -0.082 -0.065
## ego.incm.ct -0.387  0.018 -0.044 -0.002  0.165 -0.398
## ego.age     -0.049  0.000 -0.057 -0.256  0.225  0.031 -0.106
## ego.covid   -0.519  0.016 -0.010  0.026  0.004  0.096 -0.078 -0.016
```

```
ICC1 <- performance::icc(m1, by_group = TRUE)
ICC1 # estimate of ICC
```

```
## # ICC by Group
##
## Group  |   ICC
## --------------
## ego_id | 0.260
```

```
# Get confidence intervals (CIs)
se1 <- sqrt(diag(vcov(m1)))
(tab1 <- cbind(Est = fixef(m1), # table of estimates with 95% CI
              LL = fixef(m1) - 1.96 * se1,
              UL = fixef(m1) + 1.96 * se1))
```

```
##                        Est          LL          UL
## (Intercept)    -0.68771266 -1.17321931 -0.20220600
## alter.sex       0.07704076 -0.11639820  0.27047972
## alter.edu       0.45801177  0.25019985  0.66582369
## alter.age       0.15125196  0.04493957  0.25756436
## ego.sex         0.07806091 -0.26781983  0.42394166
## ego.edu        -0.28242445 -0.61339578  0.04854688
## ego.income.cat  0.18297476 -0.01441092  0.38036043
## ego.age         0.04584315 -0.10880052  0.20048683
## ego.covid       2.13807724  1.79616112  2.47999336
```

We exponentiate the estimates and CIs, to get odds ratios instead of coefficients on the logit scale

```
round((exp(tab1)),2)
```

```
##                 Est   LL    UL
## (Intercept)    0.50 0.31  0.82
## alter.sex      1.08 0.89  1.31
## alter.edu      1.58 1.28  1.95
## alter.age      1.16 1.05  1.29
## ego.sex        1.08 0.77  1.53
## ego.edu        0.75 0.54  1.05
## ego.income.cat 1.20 0.99  1.46
## ego.age        1.05 0.90  1.22
## ego.covid      8.48 6.03 11.94
```

```r
round((tab1),2) # raw estimates
```

```
##                   Est    LL    UL
## (Intercept)     -0.69 -1.17 -0.20
## alter.sex        0.08 -0.12  0.27
## alter.edu        0.46  0.25  0.67
## alter.age        0.15  0.04  0.26
## ego.sex          0.08 -0.27  0.42
## ego.edu         -0.28 -0.61  0.05
## ego.income.cat   0.18 -0.01  0.38
## ego.age          0.05 -0.11  0.20
## ego.covid        2.14  1.80  2.48
```

```r
# Standard logistic regression model ----
m1.glm <- glm(alter.covid ~
              # alters' attributes (level 1 predictors)
              alter.sex
              + alter.edu
              + alter.age
              # egos' attributes (level 2 predictors)
              + ego.sex
              + ego.edu
              + ego.income.cat
              + ego.age
              + ego.covid
              + prop.vacc.ex.alter
              , family = "binomial"
              , na.omit(alter.df3) # use only complete observations
              , set.seed(1234)
)

summary(m1.glm)
```

```
##
## Call:
## glm(formula = alter.covid ~ alter.sex + alter.edu + alter.age +
##     ego.sex + ego.edu + ego.income.cat + ego.age + ego.covid +
##     prop.vacc.ex.alter, family = "binomial", data = na.omit(alter.df3),
##     weights = set.seed(1234))
##
## Deviance Residuals:
##    Min      1Q  Median      3Q     Max
## -2.380  -0.605   0.487   0.664   1.959
##
## Coefficients:
##                      Estimate Std. Error z value Pr(>|z|)
## (Intercept)           0.55557    0.16570   3.353  0.00080 ***
## alter.sex             0.05250    0.09194   0.571  0.56796
## alter.edu             0.43072    0.09422   4.571 4.85e-06 ***
## alter.age             0.13661    0.04957   2.756  0.00585 **
## ego.sex               0.02108    0.10795   0.195  0.84519
## ego.edu              -0.23651    0.10235  -2.311  0.02085 *
## ego.income.cat        0.02173    0.06048   0.359  0.71930
## ego.age              -0.03011    0.05019  -0.600  0.54856
```

```
## ego.covid           0.71991    0.11413   6.308 2.83e-10 ***
## prop.vacc.ex.alter  0.81874    0.05229  15.659  < 2e-16 ***
## ---
## Signif. codes:  0 '***' 0.001 '**' 0.01 '*' 0.05 '.' 0.1 ' ' 1
##
## (Dispersion parameter for binomial family taken to be 1)
##
##     Null deviance: 4076.5  on 3587  degrees of freedom
## Residual deviance: 3357.5  on 3578  degrees of freedom
## AIC: 3377.5
##
## Number of Fisher Scoring iterations: 4
```

```r
# Multi-level logistic regression model
m2 <- glmer(alter.covid ~
            # node-level properties
            + ego.alter.duration
            + assortativity.var # assortativity variable
            + alter.betw
            # network-level properties
            + comp
            + dens
            + centraliz
            + (1 | ego_id) # the intercept can vary by ego (pers. netw.)
            , family = "binomial"
            , na.omit(alter.df3) # use only complete observations
            , set.seed(1234)
            , nAGQ = 100
            , control=glmerControl(optimizer = "bobyqa")) # use R's optimization routine

summary(m2)
```

Model 2 ('network model')

```
## Generalized linear mixed model fit by maximum likelihood (Adaptive
##   Gauss-Hermite Quadrature, nAGQ = 100) [glmerMod]
##  Family: binomial  ( logit )
## Formula: alter.covid ~ +ego.alter.duration + assortativity.var + alter.betw +
##     comp + dens + centraliz + (1 | ego_id)
##    Data: na.omit(alter.df3)
## Control: glmerControl(optimizer = "bobyqa")
##
##      AIC      BIC   logLik deviance df.resid
##   3576.0   3625.5  -1780.0   3560.0     3580
##
## Scaled residuals:
##     Min      1Q  Median      3Q     Max
## -4.4165 -0.4062  0.2678  0.4874  1.9653
##
## Random effects:
##  Groups Name        Variance Std.Dev.
##  ego_id (Intercept) 2.305    1.518
## Number of obs: 3588, groups:  ego_id, 401
##
## Fixed effects:
##                    Estimate Std. Error z value Pr(>|z|)
## (Intercept)         1.47303    0.09605  15.336  < 2e-16 ***
## ego.alter.duration  0.05128    0.05203   0.986    0.324
## assortativity.var   0.29509    0.04252   6.940 3.93e-12 ***
## alter.betw          0.07171    0.05056   1.419    0.156
## comp                0.03444    0.11165   0.308    0.758
## dens                0.04679    0.11995   0.390    0.696
## centraliz           0.02585    0.10703   0.241    0.809
## ---
## Signif. codes:  0 '***' 0.001 '**' 0.01 '*' 0.05 '.' 0.1 ' ' 1
##
```

```
## Correlation of Fixed Effects:
##              (Intr) eg.lt. assrt. altr.b comp   dens
## eg.ltr.drtn   0.010
## assrttvty.v   0.039  0.003
## alter.betw    0.016 -0.183  0.021
## comp         -0.047 -0.055  0.002  0.119
## dens         -0.007 -0.069 -0.004  0.133  0.609
## centraliz    -0.021 -0.029 -0.015  0.033  0.441  0.443
```

```
ICC2 <- performance::icc(m2, by_group = TRUE)
ICC2 # estimate of ICC
```

```
## # ICC by Group
##
## Group  |   ICC
## --------------
## ego_id | 0.412
```

```
#Get confidence intervals (CIs)
se2 <- sqrt(diag(vcov(m2)))
(tab2 <- cbind(Est = fixef(m2), # table of estimates with 95% CI
               LL = fixef(m2) - 1.96 * se2,
               UL = fixef(m2) + 1.96 * se2))
```

```
##                           Est          LL        UL
## (Intercept)        1.47303238  1.28477353 1.6612912
## ego.alter.duration 0.05127790 -0.05069904 0.1532548
## assortativity.var  0.29508629  0.21174331 0.3784293
## alter.betw         0.07171474 -0.02737419 0.1708037
## comp               0.03443973 -0.18439253 0.2532720
## dens               0.04679128 -0.18831204 0.2818946
## centraliz          0.02584596 -0.18392343 0.2356154
```

We exponentiate the estimates and CIs, to get odds ratios instead of coefficients on the logit scale

```
round((exp(tab2)),2)
```

```
##                     Est   LL   UL
## (Intercept)        4.36 3.61 5.27
## ego.alter.duration 1.05 0.95 1.17
## assortativity.var  1.34 1.24 1.46
## alter.betw         1.07 0.97 1.19
## comp               1.04 0.83 1.29
## dens               1.05 0.83 1.33
## centraliz          1.03 0.83 1.27
```

```
round((tab2),2) # raw estimates
```

```
##                     Est    LL   UL
## (Intercept)        1.47  1.28 1.66
## ego.alter.duration 0.05 -0.05 0.15
## assortativity.var  0.30  0.21 0.38
## alter.betw         0.07 -0.03 0.17
## comp               0.03 -0.18 0.25
## dens               0.05 -0.19 0.28
## centraliz          0.03 -0.18 0.24
```

```r
# Standard logistic regression model
m2.glm <- glm(alter.covid ~
              # node-level properties
              + ego.alter.duration
              + assortativity.var # assortativity
              + alter.betw
              # network-level properties
              + comp
              + dens
              + centraliz

              + prop.vacc.ex.alter

              , family = "binomial"
              , na.omit(alter.df3) # use only complete observations
              , set.seed(1234)
)

summary(m2.glm)
```

```
##
## Call:
## glm(formula = alter.covid ~ +ego.alter.duration + assortativity.var +
##     alter.betw + comp + dens + centraliz + prop.vacc.ex.alter,
##     family = "binomial", data = na.omit(alter.df3), weights = set.seed(1234))
##
## Deviance Residuals:
##     Min       1Q   Median       3Q      Max
## -2.4198  -0.6500   0.4809   0.6561   1.9251
##
## Coefficients:
##                    Estimate Std. Error z value Pr(>|z|)
## (Intercept)         1.28271    0.04550  28.191  < 2e-16 ***
## ego.alter.duration  0.03643    0.04436   0.821   0.4115
## assortativity.var   0.27770    0.03749   7.406  1.3e-13 ***
## alter.betw          0.08201    0.04704   1.743   0.0813 .
## comp                0.05171    0.05622   0.920   0.3577
## dens                0.07001    0.05774   1.213   0.2253
## centraliz           0.03449    0.05003   0.690   0.4905
## prop.vacc.ex.alter  1.01268    0.04389  23.072  < 2e-16 ***
## ---
## Signif. codes:  0 '***' 0.001 '**' 0.01 '*' 0.05 '.' 0.1 ' ' 1
##
## (Dispersion parameter for binomial family taken to be 1)
##
##     Null deviance: 4076.5  on 3587  degrees of freedom
## Residual deviance: 3364.6  on 3580  degrees of freedom
## AIC: 3380.6
##
## Number of Fisher Scoring iterations: 4
```

```r
# Multi-level logistic regression model
m3 <- glmer(alter.covid ~
          # alters' attributes (level 1 predictors)
              alter.sex
            + alter.edu
            + alter.age
            # egos' attributes (level 2 predictors)
            + ego.sex
            + ego.edu
            + ego.income.cat
            + ego.age
            + ego.covid
            # node-level properties
            + ego.alter.duration
            + assortativity.var # assortativity
            + alter.betw
            # network-level properties
            + comp
            + dens
            + centraliz
            + (1 | ego_id) # the intercept can vary by ego (pers. netw.)
            , family = "binomial"
            , na.omit(alter.df3) # use only complete observations
            , set.seed(1234)
            , nAGQ = 100
            , control=glmerControl(optimizer = "bobyqa")) # use R's optimization routine


summary(m3)
```

Model 3 ('full model')

```
## Generalized linear mixed model fit by maximum likelihood (Adaptive
##   Gauss-Hermite Quadrature, nAGQ = 100) [glmerMod]
##  Family: binomial  ( logit )
## Formula: alter.covid ~ alter.sex + alter.edu + alter.age + ego.sex + ego.edu +
##     ego.income.cat + ego.age + ego.covid + ego.alter.duration +
##     assortativity.var + alter.betw + comp + dens + centraliz +
##     (1 | ego_id)
##    Data: na.omit(alter.df3)
## Control: glmerControl(optimizer = "bobyqa")
##
##      AIC      BIC   logLik deviance df.resid
##   3428.7   3527.7  -1698.3   3396.7     3572
##
## Scaled residuals:
##     Min      1Q  Median      3Q     Max
## -4.3709 -0.3958  0.3039  0.4586  2.1455
##
## Random effects:
##  Groups Name        Variance Std.Dev.
##  ego_id (Intercept) 1.183    1.088
## Number of obs: 3588, groups:  ego_id, 401
```

```
##
## Fixed effects:
##                      Estimate Std. Error z value Pr(>|z|)
## (Intercept)         -0.6638284  0.2518847  -2.635  0.00840 **
## alter.sex            0.1133714  0.1006011   1.127  0.25977
## alter.edu            0.4385199  0.1074968   4.079 4.52e-05 ***
## alter.age            0.1611565  0.0603157   2.672  0.00754 **
## ego.sex              0.0597993  0.1794215   0.333  0.73892
## ego.edu             -0.2832974  0.1731318  -1.636  0.10177
## ego.income.cat       0.1892560  0.1024463   1.847  0.06469 .
## ego.age              0.0414790  0.0816748   0.508  0.61155
## ego.covid            2.1158357  0.1765166  11.987  < 2e-16 ***
## ego.alter.duration   0.0001983  0.0596169   0.003  0.99735
## assortativity.var    0.2734970  0.0424730   6.439 1.20e-10 ***
## alter.betw           0.0981108  0.0517184   1.897  0.05783 .
## comp                 0.0930267  0.0919916   1.011  0.31190
## dens                 0.0911704  0.0964507   0.945  0.34453
## centraliz            0.0291055  0.0862102   0.338  0.73566
## ---
## Signif. codes:  0 '***' 0.001 '**' 0.01 '*' 0.05 '.' 0.1 ' ' 1
##
## Correlation matrix not shown by default, as p = 15 > 12.
## Use print(x, correlation=TRUE)  or
##     vcov(x)        if you need it
```

```
vcov(m3) # print correlation matrix
```

```
## 15 x 15 Matrix of class "dpoMatrix"
##                       (Intercept)     alter.sex     alter.edu     alter.age
## (Intercept)          0.0634458776 -3.603868e-03 -2.378139e-03 -1.357172e-04
## alter.sex           -0.0036038684  1.012057e-02 -9.628202e-04 -3.632643e-04
## alter.edu           -0.0023781388 -9.628202e-04  1.155556e-02  1.694286e-03
## alter.age           -0.0001357172 -3.632643e-04  1.694286e-03  3.637986e-03
## ego.sex             -0.0250373215 -3.113659e-03  3.087886e-04  7.900578e-06
## ego.edu             -0.0095230322 -1.990233e-04 -3.030512e-03 -7.642590e-04
## ego.income.cat      -0.0099069717  2.042525e-04 -4.816169e-04 -3.648526e-05
## ego.age             -0.0008810989 -8.899615e-05 -6.024835e-04 -7.124024e-04
## ego.covid           -0.0230844516  3.401234e-04 -1.923776e-04  3.048322e-04
## ego.alter.duration -0.0003806593  2.493576e-04  3.211874e-04 -1.470185e-03
## assortativity.var    0.0001827492  1.189406e-04 -1.008463e-04  5.740180e-05
## alter.betw          -0.0002827831  4.901893e-04  1.099197e-05  5.024977e-04
## comp                 0.0002792319  1.112545e-04  2.830229e-04  9.514608e-05
## dens                -0.0006341460  2.751144e-04  3.750285e-04  1.320148e-04
## centraliz            0.0015317182 -4.025262e-06 -1.353788e-04 -9.173867e-05
##                           ego.sex       ego.edu ego.income.cat       ego.age
## (Intercept)         -2.503732e-02 -9.523032e-03  -9.906972e-03 -8.810989e-04
## alter.sex           -3.113659e-03 -1.990233e-04   2.042525e-04 -8.899615e-05
## alter.edu            3.087886e-04 -3.030512e-03  -4.816169e-04 -6.024835e-04
## alter.age            7.900578e-06 -7.642590e-04  -3.648526e-05 -7.124024e-04
## ego.sex              3.219208e-02 -1.723103e-03   2.993973e-03  3.209438e-03
## ego.edu             -1.723103e-03  2.997462e-02  -7.167844e-03  3.737651e-04
## ego.income.cat       2.993973e-03 -7.167844e-03   1.049525e-02 -8.502195e-04
## ego.age              3.209438e-03  3.737651e-04  -8.502195e-04  6.670765e-03
## ego.covid            1.847064e-04  2.970313e-03  -1.418794e-03 -2.223099e-04
```

```
## ego.alter.duration  2.482452e-05  4.441529e-05   6.351266e-05 -9.855623e-04
## assortativity.var  -6.207176e-05  6.963576e-05   5.250215e-06  7.027529e-06
## alter.betw         -1.790137e-04 -3.167312e-06   6.061504e-06  1.760376e-04
## comp               -4.259217e-04 -2.254115e-03   7.557767e-04  4.166824e-05
## dens                1.324824e-04 -4.216691e-04   1.401181e-04 -2.745059e-04
## centraliz          -9.999143e-04 -1.052026e-03  -9.504317e-05 -1.596288e-04
##                         ego.covid ego.alter.duration assortativity.var
## (Intercept)        -2.308445e-02      -3.806593e-04      1.827492e-04
## alter.sex           3.401234e-04       2.493576e-04      1.189406e-04
## alter.edu          -1.923776e-04       3.211874e-04     -1.008463e-04
## alter.age           3.048322e-04      -1.470185e-03      5.740180e-05
## ego.sex             1.847064e-04       2.482452e-05     -6.207176e-05
## ego.edu             2.970313e-03       4.441529e-05      6.963576e-05
## ego.income.cat     -1.418794e-03       6.351266e-05      5.250215e-06
## ego.age            -2.223099e-04      -9.855623e-04      7.027529e-06
## ego.covid           3.115811e-02      -7.712057e-05     -2.489903e-05
## ego.alter.duration -7.712057e-05       3.554175e-03     -3.059471e-05
## assortativity.var  -2.489903e-05      -3.059471e-05      1.803958e-03
## alter.betw          2.900375e-04      -7.215567e-04      5.270726e-05
## comp                1.280617e-04      -3.368913e-04      2.450397e-05
## dens                3.959472e-04      -3.778960e-04     -1.563006e-05
## centraliz          -5.523050e-05      -1.179894e-04     -7.450755e-05
##                        alter.betw          comp          dens     centraliz
## (Intercept)        -2.827831e-04  2.792319e-04 -6.341460e-04  1.531718e-03
## alter.sex           4.901893e-04  1.112545e-04  2.751144e-04 -4.025262e-06
## alter.edu           1.099197e-05  2.830229e-04  3.750285e-04 -1.353788e-04
## alter.age           5.024977e-04  9.514608e-05  1.320148e-04 -9.173867e-05
## ego.sex            -1.790137e-04 -4.259217e-04  1.324824e-04 -9.999143e-04
## ego.edu            -3.167312e-06 -2.254115e-03 -4.216691e-04 -1.052026e-03
## ego.income.cat      6.061504e-06  7.557767e-04  1.401181e-04 -9.504317e-05
## ego.age             1.760376e-04  4.166824e-05 -2.745059e-04 -1.596288e-04
## ego.covid           2.900375e-04  1.280617e-04  3.959472e-04 -5.523050e-05
## ego.alter.duration -7.215567e-04 -3.368913e-04 -3.778960e-04 -1.179894e-04
## assortativity.var   5.270726e-05  2.450397e-05 -1.563006e-05 -7.450755e-05
## alter.betw          2.674790e-03  6.813260e-04  8.082328e-04  1.533354e-04
## comp                6.813260e-04  8.462449e-03  5.342547e-03  3.494531e-03
## dens                8.082328e-04  5.342547e-03  9.302742e-03  3.645936e-03
## centraliz           1.533354e-04  3.494531e-03  3.645936e-03  7.432205e-03
```

```
ICC3 <- performance::icc(m3, by_group = TRUE)
ICC3 # estimate of ICC
```

```
## # ICC by Group
##
## Group  |   ICC
## ---------------
## ego_id | 0.265
```

```
# Get confidence intervals (CIs)
se3 <- sqrt(diag(vcov(m3)))
(tab3 <- cbind(Est = fixef(m3), # table of estimates with 95% CI
               LL = fixef(m3) - 1.96 * se3,
               UL = fixef(m3) + 1.96 * se3))
```

```
##                                 Est            LL          UL
```

```
## (Intercept)        -0.6638283761 -1.157522293 -0.1701345
## alter.sex           0.1133714057 -0.083806670  0.3105495
## alter.edu           0.4385198669  0.227826122  0.6492136
## alter.age           0.1611564537  0.042937645  0.2793753
## ego.sex             0.0597993126 -0.291866831  0.4114655
## ego.edu            -0.2832974412 -0.622635779  0.0560409
## ego.income.cat      0.1892560253 -0.011538808  0.3900509
## ego.age             0.0414789583 -0.118603558  0.2015615
## ego.covid           2.1158356981  1.769863136  2.4618083
## ego.alter.duration  0.0001983095 -0.116650815  0.1170474
## assortativity.var   0.2734970276  0.190249893  0.3567442
## alter.betw          0.0981108480 -0.003257161  0.1994789
## comp                0.0930267188 -0.087276759  0.2733302
## dens                0.0911703729 -0.097873040  0.2802138
## centraliz           0.0291054664 -0.139866603  0.1980775
```

We exponentiate the estimates and CIs, to get odds ratios instead of coefficients on the logit scale

```
round((exp(tab3)),2)
```

```
##                     Est   LL    UL
## (Intercept)        0.51 0.31  0.84
## alter.sex          1.12 0.92  1.36
## alter.edu          1.55 1.26  1.91
## alter.age          1.17 1.04  1.32
## ego.sex            1.06 0.75  1.51
## ego.edu            0.75 0.54  1.06
## ego.income.cat     1.21 0.99  1.48
## ego.age            1.04 0.89  1.22
## ego.covid          8.30 5.87 11.73
## ego.alter.duration 1.00 0.89  1.12
## assortativity.var  1.31 1.21  1.43
## alter.betw         1.10 1.00  1.22
## comp               1.10 0.92  1.31
## dens               1.10 0.91  1.32
## centraliz          1.03 0.87  1.22
```

```
round((tab3),2) # raw estimates
```

```
##                      Est    LL    UL
## (Intercept)        -0.66 -1.16 -0.17
## alter.sex           0.11 -0.08  0.31
## alter.edu           0.44  0.23  0.65
## alter.age           0.16  0.04  0.28
## ego.sex             0.06 -0.29  0.41
## ego.edu            -0.28 -0.62  0.06
## ego.income.cat      0.19 -0.01  0.39
## ego.age             0.04 -0.12  0.20
## ego.covid           2.12  1.77  2.46
## ego.alter.duration  0.00 -0.12  0.12
## assortativity.var   0.27  0.19  0.36
## alter.betw          0.10  0.00  0.20
## comp                0.09 -0.09  0.27
## dens                0.09 -0.10  0.28
## centraliz           0.03 -0.14  0.20
```

```r
# Standard logistic regression model
m3.glm <- glm(alter.covid ~
           # alters' attributes (level 1 predictors)
                alter.sex
              + alter.edu
              + alter.age
              # egos' attributes (level 2 predictors)
              + ego.sex
              + ego.edu
              + ego.income.cat
              + ego.age
              + ego.covid
              # node-level properties
              + ego.alter.duration

              + assortativity.var
              + alter.betw
              # network-level properties
              + comp
              + dens
              + centraliz
              + prop.vacc.ex.alter

              , family = "binomial"
              , na.omit(alter.df3) # use only complete observations
              , set.seed(1234)
)


summary(m3.glm)
```

```
##
## Call:
## glm(formula = alter.covid ~ alter.sex + alter.edu + alter.age +
##     ego.sex + ego.edu + ego.income.cat + ego.age + ego.covid +
##     ego.alter.duration + assortativity.var + alter.betw + comp +
##     dens + centraliz + prop.vacc.ex.alter, family = "binomial",
##     data = na.omit(alter.df3), weights = set.seed(1234))
##
## Deviance Residuals:
##     Min       1Q   Median       3Q      Max
## -2.4271  -0.5950   0.4775   0.6393   1.9469
##
## Coefficients:
##                     Estimate Std. Error z value Pr(>|z|)
## (Intercept)         0.609283   0.168143   3.624 0.000291 ***
## alter.sex           0.093458   0.093584   0.999 0.317959
## alter.edu           0.408938   0.095656   4.275 1.91e-05 ***
## alter.age           0.137236   0.055268   2.483 0.013024 *
## ego.sex            -0.001669   0.109795  -0.015 0.987869
## ego.edu            -0.226983   0.104739  -2.167 0.030226 *
## ego.income.cat      0.021095   0.061416   0.343 0.731235
## ego.age            -0.042454   0.053044  -0.800 0.423506
## ego.covid           0.666393   0.116114   5.739 9.52e-09 ***
```

```
## ego.alter.duration  0.014591   0.055572   0.263 0.792891    
## assortativity.var   0.259223   0.038330   6.763 1.35e-11 ***
## alter.betw          0.095490   0.048690   1.961 0.049856 *  
## comp                0.077810   0.057380   1.356 0.175081    
## dens                0.093930   0.058348   1.610 0.107435    
## centraliz           0.036950   0.050787   0.728 0.466895    
## prop.vacc.ex.alter  0.837285   0.052974  15.806  < 2e-16 ***
## ---
## Signif. codes:  0 '***' 0.001 '**' 0.01 '*' 0.05 '.' 0.1 ' ' 1
## 
## (Dispersion parameter for binomial family taken to be 1)
## 
##     Null deviance: 4076.5  on 3587  degrees of freedom
## Residual deviance: 3306.2  on 3572  degrees of freedom
## AIC: 3338.2
## 
## Number of Fisher Scoring iterations: 4
```

```
library(sjPlot)
library(sjmisc)
library(sjlabelled)
library(jtools)
```

2.2.2 Print other results

```
# Print log-Likelihood
my_models <- list(m0, m1, m2, m3)
names(my_models) <- c("null_model", "attributes_model", "network_model", "full_model")
my_logLik <- lapply(my_models, logLik)
my_logLik
```

Log-likelihood, AIC, BIC

```
## $null_model
## 'log Lik.' -1806.144 (df=2)
##
## $attributes_model
## 'log Lik.' -1721.305 (df=10)
##
## $network_model
## 'log Lik.' -1780.003 (df=8)
##
## $full_model
## 'log Lik.' -1698.35 (df=16)
```

```
# Print AIC scores for each model
my_aic <- lapply(my_models, AIC)
my_aic
```

```
## $null_model
## [1] 3616.287
##
## $attributes_model
## [1] 3462.61
##
## $network_model
## [1] 3576.006
##
## $full_model
## [1] 3428.7
```

```
# Print BIC scores for each model
my_bic <- lapply(my_models, BIC)
my_bic
```

```
## $null_model
## [1] 3628.658
##
## $attributes_model
## [1] 3524.464
##
## $network_model
```

```
## [1] 3625.489
##
## $full_model
## [1] 3527.665
```

```
# Print log-Likelihood
my_glm_models <- list(m0.glm, m1.glm, m2.glm, m3.glm)
names(my_glm_models) <- c("null_model", "attributes_model", "network_model", "full_model")
my_glm_logLik <- lapply(my_glm_models, logLik)
my_glm_logLik
```

```
## $null_model
## 'log Lik.' -1712.485 (df=2)
##
## $attributes_model
## 'log Lik.' -1678.763 (df=10)
##
## $network_model
## 'log Lik.' -1682.285 (df=8)
##
## $full_model
## 'log Lik.' -1653.085 (df=16)
```

```
# Print AIC scores for each model
my_glm_aic <- lapply(my_glm_models, AIC)
my_glm_aic
```

```
## $null_model
## [1] 3428.97
##
## $attributes_model
## [1] 3377.527
##
## $network_model
## [1] 3380.57
##
## $full_model
## [1] 3338.17
```

```
# Print BIC scores for each model
my_glm_bic <- lapply(my_glm_models, BIC)
my_glm_bic
```

```
## $null_model
## [1] 3441.34
##
## $attributes_model
## [1] 3439.38
##
## $network_model
## [1] 3430.053
##
## $full_model
## [1] 3437.136
```

Confidence intervals for the estimates We compute confidence intervals for estimates in each model standard logistic regression model

```
library(MASS)
```

```
round(confint(m0.glm),2) # model 0
```

```
## Waiting for profiling to be done...
```

```
##                    2.5 % 97.5 %
## (Intercept)         1.18   1.36
## prop.vacc.ex.alter  0.93   1.10
```

```
round(confint(m1.glm),2) # model 1
```

```
## Waiting for profiling to be done...
```

```
##                    2.5 % 97.5 %
## (Intercept)         0.23   0.88
## alter.sex          -0.13   0.23
## alter.edu           0.25   0.62
## alter.age           0.04   0.23
## ego.sex            -0.19   0.23
## ego.edu            -0.44  -0.04
## ego.income.cat     -0.10   0.14
## ego.age            -0.13   0.07
## ego.covid           0.50   0.94
## prop.vacc.ex.alter  0.72   0.92
```

```
round(confint(m2.glm),2) # model 2
```

```
## Waiting for profiling to be done...
```

```
##                    2.5 % 97.5 %
## (Intercept)         1.19   1.37
## ego.alter.duration -0.05   0.12
## assortativity.var   0.20   0.35
## alter.betw         -0.01   0.18
## comp               -0.06   0.16
## dens               -0.04   0.18
## centraliz          -0.06   0.13
## prop.vacc.ex.alter  0.93   1.10
```

```
round(confint(m3.glm),2) # model 3
```

```
## Waiting for profiling to be done...
```

```
##                    2.5 % 97.5 %
## (Intercept)         0.28   0.94
## alter.sex          -0.09   0.28
## alter.edu           0.22   0.60
## alter.age           0.03   0.25
## ego.sex            -0.22   0.21
## ego.edu            -0.43  -0.02
## ego.income.cat     -0.10   0.14
## ego.age            -0.15   0.06
## ego.covid           0.44   0.89
## ego.alter.duration -0.09   0.12
## assortativity.var   0.18   0.33
```

```
## alter.betw          0.00   0.19
## comp               -0.03   0.19
## dens               -0.02   0.21
## centraliz          -0.06   0.14
## prop.vacc.ex.alter  0.73   0.94
```

```
# Compute deviance for each model
m0.glm.deviance <- round((m0.glm$deviance),2) # model 0
paste(c("deviance for model 0 is", m0.glm.deviance))
```

Model deviance

```
## [1] "deviance for model 0 is" "3424.97"
```

```
m1.glm.deviance <-round((m1.glm$deviance),2) # model 1
paste(c("deviance for model 1 is", m1.glm.deviance))
```

```
## [1] "deviance for model 1 is" "3357.53"
```

```
m2.glm.deviance <-round((m2.glm$deviance),2) # model 2
paste(c("deviance for model 2 is", m2.glm.deviance))
```

```
## [1] "deviance for model 2 is" "3364.57"
```

```
m3.glm.deviance <-round((m3.glm$deviance),2) # model 3
paste(c("deviance for model 3 is", m3.glm.deviance))
```

```
## [1] "deviance for model 3 is" "3306.17"
```

Model comparison We compare the models (both classes: multi-level and standard)

```
# Comparing multi-level logistic regression models using anova
anova(m0, m1, m2, m3,
      test = "Chisq")
```

```
## Data: na.omit(alter.df3)
## Models:
## m0: alter.covid ~ 1 + (1 | ego_id)
## m2: alter.covid ~ +ego.alter.duration + assortativity.var + alter.betw + comp + dens + centraliz + (
## m1: alter.covid ~ alter.sex + alter.edu + alter.age + ego.sex + ego.edu + ego.income.cat + ego.age +
## m3: alter.covid ~ alter.sex + alter.edu + alter.age + ego.sex + ego.edu + ego.income.cat + ego.age +
##    npar    AIC    BIC  logLik deviance   Chisq Df Pr(>Chisq)
## m0    2 3616.3 3628.7 -1806.1   3612.3
## m2    8 3576.0 3625.5 -1780.0   3560.0  52.281  6  1.637e-09 ***
## m1   10 3462.6 3524.5 -1721.3   3442.6 117.396  2  < 2.2e-16 ***
## m3   16 3428.7 3527.7 -1698.3   3396.7  45.911  6  3.084e-08 ***
## ---
## Signif. codes:  0 '***' 0.001 '**' 0.01 '*' 0.05 '.' 0.1 ' ' 1
```

```
# Comparing standard logistic regression models using anova
anova(m0.glm, m1.glm, m2.glm, m3.glm,
      test = "Chisq")
```

```
## Analysis of Deviance Table
##
## Model 1: alter.covid ~ 1 + prop.vacc.ex.alter
## Model 2: alter.covid ~ alter.sex + alter.edu + alter.age + ego.sex + ego.edu +
```

```
##     ego.income.cat + ego.age + ego.covid + prop.vacc.ex.alter
## Model 3: alter.covid ~ +ego.alter.duration + assortativity.var + alter.betw +
##     comp + dens + centraliz + prop.vacc.ex.alter
## Model 4: alter.covid ~ alter.sex + alter.edu + alter.age + ego.sex + ego.edu +
##     ego.income.cat + ego.age + ego.covid + ego.alter.duration +
##     assortativity.var + alter.betw + comp + dens + centraliz +
##     prop.vacc.ex.alter
##   Resid. Df Resid. Dev Df Deviance  Pr(>Chi)
## 1      3586     3425.0
## 2      3578     3357.5  8   67.443 1.584e-11 ***
## 3      3580     3364.6 -2   -7.044   0.02954 *
## 4      3572     3306.2  8   58.400 9.591e-10 ***
## ---
## Signif. codes:  0 '***' 0.001 '**' 0.01 '*' 0.05 '.' 0.1 ' ' 1
```

```r
library(lmtest)
```

```r
# Compare multi-level logistic regression models using likelihood ratio test
lrtest(m0, m1, m2, m3)
```

```
## Likelihood ratio test
##
## Model 1: alter.covid ~ 1 + (1 | ego_id)
## Model 2: alter.covid ~ alter.sex + alter.edu + alter.age + ego.sex + ego.edu +
##     ego.income.cat + ego.age + ego.covid + (1 | ego_id)
## Model 3: alter.covid ~ +ego.alter.duration + assortativity.var + alter.betw +
##     comp + dens + centraliz + (1 | ego_id)
## Model 4: alter.covid ~ alter.sex + alter.edu + alter.age + ego.sex + ego.edu +
##     ego.income.cat + ego.age + ego.covid + ego.alter.duration +
##     assortativity.var + alter.betw + comp + dens + centraliz +
##     (1 | ego_id)
##   #Df  LogLik Df  Chisq Pr(>Chisq)
## 1   2 -1806.1
## 2  10 -1721.3  8 169.68  < 2.2e-16 ***
## 3   8 -1780.0 -2 117.40  < 2.2e-16 ***
## 4  16 -1698.3  8 163.31  < 2.2e-16 ***
## ---
## Signif. codes:  0 '***' 0.001 '**' 0.01 '*' 0.05 '.' 0.1 ' ' 1
```

```r
# Compare standard logistic regression models using likelihood ratio test
lrtest(m0.glm, m1.glm, m2.glm, m3.glm)
```

```
## Likelihood ratio test
##
## Model 1: alter.covid ~ 1 + prop.vacc.ex.alter
## Model 2: alter.covid ~ alter.sex + alter.edu + alter.age + ego.sex + ego.edu +
##     ego.income.cat + ego.age + ego.covid + prop.vacc.ex.alter
## Model 3: alter.covid ~ +ego.alter.duration + assortativity.var + alter.betw +
##     comp + dens + centraliz + prop.vacc.ex.alter
## Model 4: alter.covid ~ alter.sex + alter.edu + alter.age + ego.sex + ego.edu +
##     ego.income.cat + ego.age + ego.covid + ego.alter.duration +
##     assortativity.var + alter.betw + comp + dens + centraliz +
##     prop.vacc.ex.alter
##   #Df  LogLik Df   Chisq Pr(>Chisq)
## 1   2 -1712.5
## 2  10 -1678.8  8 67.4428  1.584e-11 ***
```

```
## 3   8 -1682.3 -2  7.0437    0.02954 *
## 4  16 -1653.1  8 58.4003  9.591e-10 ***
## ---
## Signif. codes:  0 '***' 0.001 '**' 0.01 '*' 0.05 '.' 0.1 ' ' 1
```

Multi-collinearity We inspect multi-collinearity in multi-level logistic regression models. Multicollinearity (reasons for concern: vif >4)

```
library(car)
```

```
(vif.m1 <- car::vif(m1))
```

```
##      alter.sex      alter.edu      alter.age        ego.sex        ego.edu
##       1.042675       1.154939       1.188755       1.136133       1.247218
## ego.income.cat        ego.age      ego.covid
##       1.270088       1.162738       1.013682
```

```
(vif.m1>4) # model 1 (multilevel)
```

```
##      alter.sex      alter.edu      alter.age        ego.sex        ego.edu
##          FALSE          FALSE          FALSE          FALSE          FALSE
## ego.income.cat        ego.age      ego.covid
##          FALSE          FALSE          FALSE
```

```
(vif.m2 <- car::vif(m2))
```

```
## ego.alter.duration  assortativity.var         alter.betw               comp
##           1.036950           1.000822           1.055118           1.691929
##               dens          centraliz
##           1.704894           1.323793
```

```
(vif.m2>4) # model 2 (multilevel)
```

```
## ego.alter.duration  assortativity.var         alter.betw               comp
##              FALSE              FALSE              FALSE              FALSE
##               dens          centraliz
##              FALSE              FALSE
```

```
(vif.m3 <- car::vif(m3))
```

```
##          alter.sex          alter.edu          alter.age            ego.sex
##           1.060569           1.161965           1.436586           1.146727
##            ego.edu     ego.income.cat            ego.age          ego.covid
##           1.281867           1.284387           1.216965           1.015704
## ego.alter.duration  assortativity.var         alter.betw               comp
##           1.409964           1.003213           1.109773           1.726637
##               dens          centraliz
##           1.715429           1.338494
```

```
(vif.m3>4) # model 3 (multilevel)
```

```
##          alter.sex          alter.edu          alter.age            ego.sex
##              FALSE              FALSE              FALSE              FALSE
##            ego.edu     ego.income.cat            ego.age          ego.covid
##              FALSE              FALSE              FALSE              FALSE
## ego.alter.duration  assortativity.var         alter.betw               comp
##              FALSE              FALSE              FALSE              FALSE
##               dens          centraliz
##              FALSE              FALSE
```

```r
(vif.m1.glm <- car::vif(m1.glm))
```

```
##          alter.sex          alter.edu          alter.age            ego.sex
##           1.086171           1.187425           1.281257           1.197984
##            ego.edu     ego.income.cat            ego.age          ego.covid
##           1.280022           1.274780           1.300391           1.407043
## prop.vacc.ex.alter
##           1.435424
```

```r
(vif.m1.glm>4) # model 1 (standard)
```

```
##          alter.sex          alter.edu          alter.age            ego.sex
##              FALSE              FALSE              FALSE              FALSE
##            ego.edu     ego.income.cat            ego.age          ego.covid
##              FALSE              FALSE              FALSE              FALSE
## prop.vacc.ex.alter
##              FALSE
```

```r
(vif.m2.glm <- car::vif(m2.glm))
```

```
## ego.alter.duration  assortativity.var         alter.betw               comp
##           1.031836           1.001632           1.109483           1.677929
##               dens          centraliz prop.vacc.ex.alter
##           1.751255           1.308890           1.006770
```

```r
(vif.m2.glm>4) # model 2 (standard)
```

```
## ego.alter.duration  assortativity.var         alter.betw               comp
##              FALSE              FALSE              FALSE              FALSE
##               dens          centraliz prop.vacc.ex.alter
##              FALSE              FALSE              FALSE
```

```r
(vif.m3.glm <- car::vif(m3.glm))
```

```
##          alter.sex          alter.edu          alter.age            ego.sex
##           1.105650           1.200214           1.559161           1.215098
##            ego.edu     ego.income.cat            ego.age          ego.covid
##           1.316120           1.289585           1.425881           1.424728
## ego.alter.duration  assortativity.var         alter.betw               comp
##           1.580171           1.007086           1.158171           1.716178
##               dens          centraliz prop.vacc.ex.alter
##           1.754051           1.326787           1.459287
```

```r
(vif.m3.glm>4) # model 3 (standard)
```

```
##          alter.sex          alter.edu          alter.age            ego.sex
##              FALSE              FALSE              FALSE              FALSE
##            ego.edu     ego.income.cat            ego.age          ego.covid
##              FALSE              FALSE              FALSE              FALSE
## ego.alter.duration  assortativity.var         alter.betw               comp
##              FALSE              FALSE              FALSE              FALSE
##               dens          centraliz prop.vacc.ex.alter
##              FALSE              FALSE              FALSE
```

Model fit assessment We assess the fit of the models by looking at the PseudoR square. We compute various algorithms in this respect

```r
library(DescTools)
```

```r
# Model 0 (null model) - standard logistic regression
DescTools::PseudoR2(m0.glm, c("McFadden", "McFaddenAdj", "Nagel",
                              "CoxSnell", "AldrichNelson", "Nagelkerke",
                              "VeallZimmermann", "McKelveyZavoina",
                              "Efron", "Tjur", "AIC", "G2",
                              "LogLik", "LogLikNull"))
```

```
##        McFadden     McFaddenAdj      Nagelkerke        CoxSnell   AldrichNelson
##       0.1598322       0.1588509       0.2445844       0.1660605       0.1536859
##      Nagelkerke VeallZimmermann McKelveyZavoina           Efron            Tjur
##       0.2445844       0.2889541       0.2365435       0.1873091       0.1889855
##             AIC              G2
##    3428.9695207     651.5606355
```

```r
# Model 1 (attributes model) - standard logistic regression
DescTools::PseudoR2(m1.glm, c("McFadden", "McFaddenAdj", "Nagel",
                              "CoxSnell", "AldrichNelson", "Nagelkerke",
                              "VeallZimmermann", "McKelveyZavoina",
                              "Efron", "Tjur", "AIC", "G2",
                              "LogLik", "LogLikNull"))
```

```
##        McFadden     McFaddenAdj      Nagelkerke        CoxSnell   AldrichNelson
##       0.1763763       0.1714702       0.2674565       0.1815894       0.1669382
##      Nagelkerke VeallZimmermann McKelveyZavoina           Efron            Tjur
##       0.2674565       0.3138706       0.2537885       0.2097560       0.2110387
##             AIC              G2
##    3377.5267160     719.0034403
```

```r
# Model 2 (network model) - standard logistic regression
DescTools::PseudoR2(m2.glm, c("McFadden", "McFaddenAdj", "Nagel",
                              "CoxSnell", "AldrichNelson", "Nagelkerke",
                              "VeallZimmermann", "McKelveyZavoina",
                              "Efron", "Tjur", "AIC", "G2",
                              "LogLik", "LogLikNull"))
```

```
##        McFadden     McFaddenAdj      Nagelkerke        CoxSnell   AldrichNelson
##       0.1746485       0.1707236       0.2650878       0.1799812       0.1655736
##      Nagelkerke VeallZimmermann McKelveyZavoina           Efron            Tjur
##       0.2650878       0.3113049       0.2518643       0.2053669       0.2073296
##             AIC              G2
##    3380.5704126     711.9597436
```

```r
# Model 3 (full model) - standard logistic regression
DescTools::PseudoR2(m3.glm, c("McFadden", "McFaddenAdj", "Nagel",
                              "CoxSnell", "AldrichNelson", "Nagelkerke",
                              "VeallZimmermann", "McKelveyZavoina",
                              "Efron", "Tjur", "AIC", "G2",
                              "LogLik", "LogLikNull"))
```

```
##        McFadden     McFaddenAdj      Nagelkerke        CoxSnell   AldrichNelson
##       0.1889745       0.1811246       0.2845871       0.1932203       0.1767546
##      Nagelkerke VeallZimmermann McKelveyZavoina           Efron            Tjur
##       0.2845871       0.3323269       0.2676566       0.2235843       0.2256365
##             AIC              G2
```

```
##     3338.1700697      770.3600865
```

3. Predicting egos' opinions (optional analysis)

We fit to the ego dataframe, logistic regression models to predict ego's opinions concerning COVID-19 vaccination

3.1 Descriptive statistics

```
# ego's opinion about COVID-19 vaccination

dat.ego.factor <- ego.df # we use the ego dataset: ego-level variables
dat.ego.factor$ego.covid <- factor(dat.ego.factor$ego.covid,
                                    levels = c(0,1),
                                    labels = c("'very bad & bad' opinion",
                                               "'very good & good' opinion"))
freq(dat.ego.factor$ego.covid)
```

3.1.1 Factor variables

```
## Frequencies
## dat.ego.factor$ego.covid
## Type: Factor
##
##                                    Freq   % Valid   % Valid Cum.   % Total   % Total Cum.
## -------------------------------- ------ --------- -------------- --------- --------------
##         'very bad & bad' opinion     87     21.43          21.43     19.64          19.64
##       'very good & good' opinion    319     78.57         100.00     72.01          91.65
##                             <NA>     37                               8.35         100.00
##                            Total    443    100.00         100.00    100.00         100.00
```

```
# ego's sex
dat.ego.factor$ego.sex <- factor(dat.ego.factor$ego.sex,
                                  levels = c(0,1),
                                  labels = c("male", "female"))
freq(dat.ego.factor$ego.sex)
```

```
## Frequencies
## dat.ego.factor$ego.sex
## Type: Factor
##
##                Freq   % Valid   % Valid Cum.   % Total   % Total Cum.
## ------------ ------ --------- -------------- --------- --------------
##         male    108     24.38          24.38     24.38          24.38
##       female    335     75.62         100.00     75.62         100.00
##         <NA>      0                               0.00         100.00
##        Total    443    100.00         100.00    100.00         100.00
```

```
# ego's education
dat.ego.factor$ego.edu <- factor(dat.ego.factor$ego.edu,
                                  levels = c(0,1),
                                  labels = c("no - higher edu.",
                                             "yes - higher edu."))

freq(dat.ego.factor$ego.edu)
```

```
## Frequencies
## dat.ego.factor$ego.edu
## Type: Factor
##
##                            Freq   % Valid   % Valid Cum.   % Total   % Total Cum.
## ----------------------- ------ --------- -------------- --------- --------------
##         no - higher edu.    158     35.67          35.67     35.67          35.67
##        yes - higher edu.    285     64.33         100.00     64.33         100.00
##                     <NA>      0                               0.00         100.00
##                    Total    443    100.00         100.00    100.00         100.00
```

```
# ego's income
dat.ego.factor$ego.income.cat <- factor(dat.ego.factor$ego.income.cat,
           levels = c(0, 1, 2, 3),
           labels = c("Less than minimum wage",
          "In-between minimum & median wage",
           "In-between median wage & median wage plus one minimum wage",
           "More than median wage plus one minimum wage"))

freq(dat.ego.factor$ego.income.cat)
```

```
## Frequencies
## dat.ego.factor$ego.income.cat
## Type: Factor
##
##                                                                   Freq   % Valid   % Valid Cum.   %
## ----------------------------------------------------------------- ------ --------- -------------- ----
##                                           Less than minimum wage     78     17.61          17.61
##                                 In-between minimum & median wage    193     43.57          61.17
##       In-between median wage & median wage plus one minimum wage    145     32.73          93.91
##                      More than median wage plus one minimum wage     27      6.09         100.00
##                                                             <NA>      0
##                                                            Total    443    100.00         100.00     1
```

```

# Egos' age
descr(ego.df$ego.age)
```

3.1.2 Numeric

```
##
## ## Basic descriptive statistics
##
##  var    type label   n NA.prc  mean   sd   se md trimmed      range iqr skew
##   dd numeric    dd 443      0 36.47 11.2 0.53 34   35.62 56 (19-75)  16 0.66
```

```
# Proportion of females in each ego's network
descr(ego.df$prop.fem)
```

```
##
## ## Basic descriptive statistics
##
##  var    type label   n NA.prc mean   sd   se  md trimmed   range iqr  skew
##   dd numeric    dd 443      0 0.61 0.19 0.01 0.6    0.63 1 (0-1) 0.3 -0.57
```

```
# Proportion of alters with higher education studies
descr(ego.df$prop.edu)
```

```
##
## ## Basic descriptive statistics
##
##   var    type label   n NA.prc mean   sd   se  md trimmed   range iqr  skew
##    dd numeric    dd 443      0  0.5 0.27 0.01 0.5     0.5 1 (0-1) 0.4 -0.08
```

```r
# The mean age of alters in each ego-network
descr(ego.df$mean.age)
```

```
##
## ## Basic descriptive statistics
##
##   var    type label   n NA.prc  mean   sd   se   md trimmed            range
##    dd numeric    dd 443      0 41.71 7.82 0.37 41.4   41.56 47.2 (23.2-70.4)
##     iqr skew
##  10.95 0.23
```

```r
# Proportion of alters in favor of vaccination in each ego-network
descr(ego.df$prop.vacc)
```

```
##
## ## Basic descriptive statistics
##
##   var    type label   n NA.prc mean   sd   se  md trimmed   range iqr  skew
##    dd numeric    dd 432   2.48 0.73 0.27 0.01 0.8    0.77 1 (0-1) 0.4 -0.98
```

```r
# Ego's betweenness
descr(ego.df$ego.betw)
```

```
##
## ## Basic descriptive statistics
##
##   var    type label   n NA.prc  mean  sd   se md trimmed     range iqr  skew
##    dd numeric    dd 443      0 24.07 8.9 0.42 25    24.6 45 (0-45)  11 -0.57
```

```r
# The pro-vaccination alter with the highest score of betweenness
descr(ego.df$alter.betw.max.pro)
```

```
##
## ## Basic descriptive statistics
##
##   var    type label   n NA.prc mean   sd   se md trimmed     range iqr skew
##    dd numeric    dd 443      0 8.73 8.59 0.41  6    7.68 34 (0-34)  14 0.82
```

```r
# Each ego's network centralization
descr(ego.df$centraliz)
```

```
##
## ## Basic descriptive statistics
##
##   var    type label   n NA.prc mean   sd   se   md trimmed         range  iqr
##    dd numeric    dd 443      0 0.31 0.15 0.01 0.31    0.31 0.73 (0-0.73) 0.18
##   skew
##   0.06
```

```r
# The number of components in each ego-network
descr(ego.df$comp)
```

```
##
## ## Basic descriptive statistics
##
##   var    type label   n NA.prc mean   sd   se md trimmed    range iqr skew
##    dd numeric    dd 443      0 1.67 1.28 0.06  1    1.39 9 (1-10)   1 3.09
```

3.2 Egos' analysis

3.2.1 The ego-models

```
m1.ego <- glm(ego.covid ~
              # network composition
                prop.fem
              + prop.edu
              + mean.age
              + prop.vacc
              # egos' attributes
              + ego.edu
              + ego.sex
              + ego.age
              + ego.income.cat
              , family = "binomial"
              , na.omit(ego.df2.complete) # use only complete observations
              , set.seed(1234)
)

summary(m1.ego)
```

Ego-Model 1 ('attributes model')

```
##
## Call:
## glm(formula = ego.covid ~ prop.fem + prop.edu + mean.age + prop.vacc +
##     ego.edu + ego.sex + ego.age + ego.income.cat, family = "binomial",
##     data = na.omit(ego.df2.complete), weights = set.seed(1234))
##
## Deviance Residuals:
##     Min       1Q   Median       3Q      Max
## -2.7351   0.1391   0.2703   0.4874   2.0461
##
## Coefficients:
##                 Estimate Std. Error z value Pr(>|z|)
## (Intercept)     1.588031   0.780636   2.034   0.0419 *
## prop.fem        0.005885   0.281112   0.021   0.9833
## prop.edu       -0.192501   0.284918  -0.676   0.4993
## mean.age       -0.512695   0.318503  -1.610   0.1075
## prop.vacc       1.985890   0.268157   7.406  1.3e-13 ***
## ego.edu         0.372705   0.596901   0.624   0.5324
## ego.sex        -0.559426   0.662259  -0.845   0.3983
## ego.age         0.142993   0.338878   0.422   0.6731
## ego.income.cat  0.290308   0.320817   0.905   0.3655
## ---
## Signif. codes:  0 '***' 0.001 '**' 0.01 '*' 0.05 '.' 0.1 ' ' 1
##
```

```
## (Dispersion parameter for binomial family taken to be 1)
##
##     Null deviance: 271.89  on 261  degrees of freedom
## Residual deviance: 163.62  on 253  degrees of freedom
## AIC: 181.62
##
## Number of Fisher Scoring iterations: 6
```

```
## Model 2: network model ----
m2.ego <- glm(ego.covid ~
              # ego-alter ties
                mean.duration
              # node-level (ego)
              + ego.betw
              # node-level (alter)
              + alter.betw.max.pro
              # network-level
              + centraliz
              + comp
              , family = "binomial"
              , na.omit(ego.df2.complete) # use only complete observations
              , set.seed(1234)

)
summary(m2.ego)
```

Ego-Model 2 ('network model')

```
##
## Call:
## glm(formula = ego.covid ~ mean.duration + ego.betw + alter.betw.max.pro +
##     centraliz + comp, family = "binomial", data = na.omit(ego.df2.complete),
##     weights = set.seed(1234))
##
## Deviance Residuals:
##     Min       1Q   Median       3Q      Max
## -2.7472   0.2713   0.5251   0.7212   1.6066
##
## Coefficients:
##                    Estimate Std. Error z value Pr(>|z|)
## (Intercept)         1.46127    0.17640   8.284  < 2e-16 ***
## mean.duration      -0.09876    0.18310  -0.539   0.5897
## ego.betw           -0.56778    0.25099  -2.262   0.0237 *
## alter.betw.max.pro  1.25552    0.26201   4.792 1.65e-06 ***
## centraliz          -0.21676    0.21494  -1.008   0.3132
## comp                0.39031    0.24720   1.579   0.1144
## ---
## Signif. codes:  0 '***' 0.001 '**' 0.01 '*' 0.05 '.' 0.1 ' ' 1
##
## (Dispersion parameter for binomial family taken to be 1)
##
##     Null deviance: 271.89  on 261  degrees of freedom
## Residual deviance: 241.76  on 256  degrees of freedom
## AIC: 253.76
```

```
##
## Number of Fisher Scoring iterations: 5
```

```
m3.ego <- glm(ego.covid ~
              # network composition
                prop.fem
              + prop.edu
              + mean.age
              + prop.vacc
              # egos' attributes
              + ego.edu
              + ego.sex
              + ego.age
              + ego.income.cat
              # ego-alter ties
              + mean.duration
              # node-level (ego)
              + ego.betw
              # node-level (alter)
              + alter.betw.max.pro
              # network-level
              + centraliz
              + comp

              , family = "binomial"
              , na.omit(ego.df2.complete) # use only complete observations
              , set.seed(1234)
)
summary(m3.ego)
```

Ego-Model 3 ('full model')

```
##
## Call:
## glm(formula = ego.covid ~ prop.fem + prop.edu + mean.age + prop.vacc +
##     ego.edu + ego.sex + ego.age + ego.income.cat + mean.duration +
##     ego.betw + alter.betw.max.pro + centraliz + comp, family = "binomial",
##     data = na.omit(ego.df2.complete), weights = set.seed(1234))
##
## Deviance Residuals:
##     Min       1Q   Median       3Q      Max
## -2.8357   0.1291   0.2474   0.4605   2.0421
##
## Coefficients:
##                    Estimate Std. Error z value Pr(>|z|)
## (Intercept)         1.48968    0.79562   1.872   0.0612 .
## prop.fem           -0.05921    0.28173  -0.210   0.8335
## prop.edu           -0.19070    0.29156  -0.654   0.5131
## mean.age           -0.76195    0.36426  -2.092   0.0365 *
## prop.vacc           1.94538    0.28225   6.892 5.49e-12 ***
## ego.edu             0.55206    0.62199   0.888   0.3748
## ego.sex            -0.44090    0.66331  -0.665   0.5062
## ego.age            -0.24724    0.41714  -0.593   0.5534
## ego.income.cat      0.23988    0.32976   0.727   0.4670
```

```
## mean.duration       0.68163    0.45815   1.488   0.1368
## ego.betw           -0.09421    0.34662  -0.272   0.7858
## alter.betw.max.pro  0.35960    0.29992   1.199   0.2305
## centraliz          -0.08757    0.25616  -0.342   0.7325
## comp               -0.05192    0.33639  -0.154   0.8773
## ---
## Signif. codes:  0 '***' 0.001 '**' 0.01 '*' 0.05 '.' 0.1 ' ' 1
##
## (Dispersion parameter for binomial family taken to be 1)
##
##     Null deviance: 271.89  on 261  degrees of freedom
## Residual deviance: 158.36  on 248  degrees of freedom
## AIC: 186.36
##
## Number of Fisher Scoring iterations: 6
```

3.2.2 Print other results

Log-likelihood, AIC, BIC

```
# Print log-Likelihood
my_models_ego <- list(m1.ego, m2.ego, m3.ego)
names(my_models_ego) <- c("attributes_ego_model", "network_ego_model", "full_ego_model")
my_logLik_ego <- lapply(my_models_ego, logLik)
my_logLik_ego
```

```
## $attributes_ego_model
## 'log Lik.' -81.81133 (df=9)
##
## $network_ego_model
## 'log Lik.' -120.8797 (df=6)
##
## $full_ego_model
## 'log Lik.' -79.18122 (df=14)
```

```
# Print AIC scores for each model
my_aic_ego <- lapply(my_models_ego, AIC)
my_aic_ego
```

```
## $attributes_ego_model
## [1] 181.6227
##
## $network_ego_model
## [1] 253.7593
##
## $full_ego_model
## [1] 186.3624
```

```
# Print BIC scores for each model
my_bic_ego <- lapply(my_models_ego, BIC)
my_bic_ego
```

```
## $attributes_ego_model
## [1] 213.7378
##
## $network_ego_model
## [1] 275.1694
##
```

```
## $full_ego_model
## [1] 236.3193
```

Confidence intervals for the estimates

We compute confidence intervals for the estimates in each ego model

```r
round(confint(m1.ego),2) # ego-model 1
```

```
## Waiting for profiling to be done...

##                 2.5 % 97.5 %
## (Intercept)      0.09   3.17
## prop.fem        -0.55   0.56
## prop.edu        -0.76   0.36
## mean.age        -1.14   0.11
## prop.vacc        1.50   2.56
## ego.edu         -0.81   1.55
## ego.sex         -1.90   0.71
## ego.age         -0.52   0.82
## ego.income.cat -0.33   0.93
```

```r
round(confint(m2.ego),2) # ego-model 2
```

```
## Waiting for profiling to be done...

##                     2.5 % 97.5 %
## (Intercept)          1.13   1.83
## mean.duration       -0.45   0.27
## ego.betw            -1.07  -0.08
## alter.betw.max.pro  0.77   1.80
## centraliz           -0.65   0.20
## comp                -0.07   0.91
```

```r
round(confint(m3.ego),2) # ego-model 3
```

```
## Waiting for profiling to be done...

##                     2.5 % 97.5 %
## (Intercept)         -0.03   3.11
## prop.fem            -0.61   0.50
## prop.edu            -0.78   0.37
## mean.age            -1.48  -0.04
## prop.vacc            1.43   2.54
## ego.edu             -0.67   1.79
## ego.sex             -1.79   0.83
## ego.age             -1.07   0.58
## ego.income.cat      -0.40   0.90
## mean.duration       -0.19   1.63
## ego.betw            -0.79   0.58
## alter.betw.max.pro -0.22   0.96
## centraliz           -0.60   0.41
## comp                -0.70   0.60
```

Model deviance

We compute deviance for each of the ego-models

```r
m1.ego.deviance <- round((m1.ego$deviance),2) # ego-model 1
paste(c("deviance of ego-model 1 is", m1.ego.deviance))
```

```
## [1] "deviance of ego-model 1 is" "163.62"
```

```r
m2.ego.deviance <- round((m2.ego$deviance),2) # ego-model 2
paste(c("deviance of ego-model 2 is", m2.ego.deviance))
```

```
## [1] "deviance of ego-model 2 is" "241.76"
```

```r
m3.ego.deviance <- round((m3.ego$deviance),2) # ego-model 3
paste(c("deviance of ego-model 3 is", m3.ego.deviance))
```

```
## [1] "deviance of ego-model 3 is" "158.36"
```

Model comparison

We compare models using ANOVA

```r
anova(m1.ego, m2.ego, m3.ego,
      test = "Chisq")
```

```
## Analysis of Deviance Table
##
## Model 1: ego.covid ~ prop.fem + prop.edu + mean.age + prop.vacc + ego.edu +
##     ego.sex + ego.age + ego.income.cat
## Model 2: ego.covid ~ mean.duration + ego.betw + alter.betw.max.pro + centraliz +
##     comp
## Model 3: ego.covid ~ prop.fem + prop.edu + mean.age + prop.vacc + ego.edu +
##     ego.sex + ego.age + ego.income.cat + mean.duration + ego.betw +
##     alter.betw.max.pro + centraliz + comp
##   Resid. Df Resid. Dev Df Deviance  Pr(>Chi)
## 1       253     163.62
## 2       256     241.76 -3  -78.137 < 2.2e-16 ***
## 3       248     158.36  8   83.397  1.01e-14 ***
## ---
## Signif. codes:  0 '***' 0.001 '**' 0.01 '*' 0.05 '.' 0.1 ' ' 1
```

We compare models using Likelihood ratio test

```r
lrtest(m1.ego, m2.ego, m3.ego)
```

```
## Likelihood ratio test
##
## Model 1: ego.covid ~ prop.fem + prop.edu + mean.age + prop.vacc + ego.edu +
##     ego.sex + ego.age + ego.income.cat
## Model 2: ego.covid ~ mean.duration + ego.betw + alter.betw.max.pro + centraliz +
##     comp
## Model 3: ego.covid ~ prop.fem + prop.edu + mean.age + prop.vacc + ego.edu +
##     ego.sex + ego.age + ego.income.cat + mean.duration + ego.betw +
##     alter.betw.max.pro + centraliz + comp
##   #Df   LogLik Df  Chisq Pr(>Chisq)
## 1   9  -81.811
## 2   6 -120.880 -3 78.137  < 2.2e-16 ***
## 3  14  -79.181  8 83.397   1.01e-14 ***
## ---
## Signif. codes:  0 '***' 0.001 '**' 0.01 '*' 0.05 '.' 0.1 ' ' 1
```

Multi-collinearity

We check for multicollinearity (reasons for concern: vif >4)

```r
(vif.m1.ego <- car::vif(m1.ego))
```

```
##       prop.fem       prop.edu       mean.age      prop.vacc        ego.edu
##       1.746947       1.607954       2.290983       1.156820       1.599052
##        ego.sex        ego.age ego.income.cat
##       1.883378       2.337072       1.247618
```

```r
(vif.m1.ego>4) # model 1 (ego-model)
```

```
##       prop.fem       prop.edu       mean.age      prop.vacc        ego.edu
##          FALSE          FALSE          FALSE          FALSE          FALSE
##        ego.sex        ego.age ego.income.cat
##          FALSE          FALSE          FALSE
```

```r
(vif.m2.ego <- car::vif(m2.ego))
```

```
##      mean.duration           ego.betw alter.betw.max.pro          centraliz
##           1.030051           2.166341           1.494297           1.434199
##               comp
##           2.331611
```

```r
(vif.m2.ego>4) # model 2 (ego-model)
```

```
##      mean.duration           ego.betw alter.betw.max.pro          centraliz
##              FALSE              FALSE              FALSE              FALSE
##               comp
##              FALSE
```

```r
(vif.m3.ego <- car::vif(m3.ego))
```

```
##           prop.fem           prop.edu           mean.age          prop.vacc
##           1.684476           1.653444           2.713731           1.268985
##            ego.edu            ego.sex            ego.age     ego.income.cat
##           1.683036           1.841891           3.488748           1.295283
##      mean.duration           ego.betw alter.betw.max.pro          centraliz
##           3.883711           2.399938           1.846119           1.430491
##               comp
##           2.430775
```

```r
(vif.m3.ego>4) # model 3 (ego-model)
```

```
##           prop.fem           prop.edu           mean.age          prop.vacc
##              FALSE              FALSE              FALSE              FALSE
##            ego.edu            ego.sex            ego.age     ego.income.cat
##              FALSE              FALSE              FALSE              FALSE
##      mean.duration           ego.betw alter.betw.max.pro          centraliz
##              FALSE              FALSE              FALSE              FALSE
##               comp
##              FALSE
```

Model fit assessment

We assess the fit of the ego-models by looking at the PseudoR^2. We compute various algorithms in this respect

```r
# Model 1 ('attributes model')
DescTools::PseudoR2(m1.ego, c("McFadden", "McFaddenAdj", "Nagel",
                              "CoxSnell", "AldrichNelson", "Nagelkerke",
                              "VeallZimmermann", "McKelveyZavoina",
```

```
                            "Efron", "Tjur", "AIC", "G2",
                            "LogLik", "LogLikNull"))
```

```
##        McFadden     McFaddenAdj      Nagelkerke        CoxSnell   AldrichNelson
##       0.3981986       0.3319949       0.5241812       0.3384879       0.2923996
##      Nagelkerke VeallZimmermann McKelveyZavoina           Efron            Tjur
##       0.5241812       0.5741651       0.5329357       0.4496241       0.4445057
##             AIC              G2
##     181.6226682     108.2654809
```

```
# Model 2 ('network model')
DescTools::PseudoR2(m2.ego, c("McFadden", "McFaddenAdj", "Nagel",
                            "CoxSnell", "AldrichNelson", "Nagelkerke",
                            "VeallZimmermann", "McKelveyZavoina",
                            "Efron", "Tjur", "AIC", "G2",
                            "LogLik", "LogLikNull"))
```

```
##        McFadden     McFaddenAdj      Nagelkerke        CoxSnell   AldrichNelson
##      0.11081337      0.06667758      0.16822392      0.10862992      0.10313546
##      Nagelkerke VeallZimmermann McKelveyZavoina           Efron            Tjur
##      0.16822392      0.20252005      0.21409662      0.14040134      0.12770852
##             AIC              G2
##    253.75930612     30.12884295
```

```
# Model 3 ('full model')
DescTools::PseudoR2(m3.ego, c("McFadden", "McFaddenAdj", "Nagel",
                            "CoxSnell", "AldrichNelson", "Nagelkerke",
                            "VeallZimmermann", "McKelveyZavoina",
                            "Efron", "Tjur", "AIC", "G2",
                            "LogLik", "LogLikNull"))
```

```
##        McFadden     McFaddenAdj      Nagelkerke        CoxSnell   AldrichNelson
##       0.4175456       0.3145621       0.5445436       0.3516368       0.3023114
##      Nagelkerke VeallZimmermann McKelveyZavoina           Efron            Tjur
##       0.5445436       0.5936283       0.5581350       0.4657614       0.4623797
##             AIC              G2
##     186.3624353     113.5257138
```

4. Network-level measurements (auxiliary section)

4.1 Components

"A `component` is defined as *a maximal set of nodes in which every node can reach every other by some path (a sequence of adjacent nodes forms a `path`).*" (Borgatti, Everett, Johnson, 2013, p. 21). In a social network, nodes from different components are disconnected (do not have a path). Therefore, for instance, information does not circulate from one component to another (assuming social networks as conduits for information).

4.2 Density

"`Density` is the number of ties in a social network, expressed as a proportion of the number of possible. Density is a network property measuring network `cohesion` (the connectedness or knittedness in a network)" (Borgatti, Everett, Johnson, 2013, p. 161). Networks that display high levels of density are networks in which, for instance, information spreads at higher rates.

4.3 Centralization

"`Centralization` refers to the extent a network is dominated by a single node. A maximally centralized network looks like a star: the node at the center of a network has ties to all other nodes, and no other ties exist. A measure of centralization is a measure of the extent to which a network resembles a star" (Borgatti, Everett, Johnson, 2013, p. 170). As the centralization increases, the dominating nodes have higher impact in controlling the information.

4.4 Betweenness centrality

"`Betweenness centrality` is a measure of how often a given node falls along the shortest path between two other nodes. [...] More specifically, it is calculated for a given focal node by computing, for each pair of nodes other than the focal node, what proportion of all the shortest paths from one to the other pass through the focal node. These proportions are summed across all pairs and the result is a single value for each node in the network. [...] Betweenness is typically interpreted in terms of the potential for controlling flows through the network – that is, playing a gatekeeping or toll-taking role. In a sense, nodes with high betweenness are in a position to threaten the network with disruption of operations. More generally, high-betweenness nodes are in a position to filter information and to color or distort it as they pass it along." (Borgatti, Everett, Johnson, 2013, p. 116)

Reference - SP Borgatti, MG Everett, JC Johnson, *Analyzing Social Networks*, London: Sage, 2013

4.5 Computing network-level variables

To compute the number of `components`, `density`, and `centralization`, first we create an `egor object` (see: https://cran.r-project.org/web/packages/egor/vignettes/using_egor.html).

We use the data files publicly available on https://doi.org/10.6084/m9.figshare.22309174.v1

```
library(egor)
library(igraph)
```

```
# create egor object
egor.obj <- egor::threefiles_to_egor(egos = ego.df,
                                     alters.df = alter.df1,
                                     edges = aa.ties,
                                     ID.vars = list (ego = "ego_id",
                                                     alter = "alter_id",
                                                     source = "from",
                                                     target = "to"))
```

We convert the egor object into a list that includes all the personal networks as `igraph` objects.

```r
# Convert the egor object to a list of igraph objects
gr.list <- egor::as_igraph(egor.obj)
```

We compute the density scores for each personal network and save these results as a data frame. The resulting data frame has 'ego_id' as a joining variable that allows transferring the `density` scores (i.e., *dens*) to the other files (*aa.ties*, *ego.df*, *alter.df*).

```r
# compute density for each personal network
density_data <- gr.list %>%
  map_dbl(edge_density) %>%
  enframe(name = "ego_id", value = "dens")
head(density_data)
```

```
## # A tibble: 6 x 2
##   ego_id  dens
##   <chr>  <dbl>
## 1 1      0.2
## 2 2      0.533
## 3 3      0.644
## 4 4      0.578
## 5 6      0.356
## 6 8      0.244
```

We compute the number of components for each personal network and save these results as a data frame. The resulting data frame has 'ego_id' as a joining variable that allows transferring the number of `components` (i.e., *comp*) to the other files (*aa.ties*, *ego.df*, *alter.df*).

```r
# compute the number of components for each personal network
components_data <- gr.list %>%
  map_dbl(~ components(.x)$no) %>%
  enframe()
names(components_data)[1:2] <- c("ego_id", "comp")
head(components_data)
```

```
## # A tibble: 6 x 2
##   ego_id  comp
##   <chr>  <dbl>
## 1 1          2
## 2 2          1
## 3 3          1
## 4 4          1
## 5 6          1
## 6 8          1
```

We compute the centralization scores for each personal network and save these results as a data frame. The resulting data frame has 'ego_id' as a joining variable that allows transferring the scores of `centralization` (i.e., *centraliz*) to the other files (*aa.ties*, *ego.df*, *alter.df*).

```r
# compute centralization for each personal network
centralization_data <- gr.list %>%
  map_dfr(~ tibble(deg.centr= centr_degree(.x)$centralization),
          .id = "ego_id")
names(centralization_data)[1:2] <- c("ego_id", "centraliz")
head(centralization_data)
```

```
## # A tibble: 6 x 2
##   ego_id centraliz
```

```
##   <chr>      <dbl>
## 1 1          0.244
## 2 2          0.467
## 3 3          0.356
## 4 4          0.311
## 5 6          0.2
## 6 8          0.2
```

We compute the betweenness centrality scores (`alter.betw`) for the alters from each personal network and save these results as a data frame. The resulting data frame has 'ego_id' as a joining variable that allows transferring the scores of `alter.betw` to the other files (*aa.ties*, *ego.df*, *alter.df*).

```r
library(igraph)
library(dplyr)
library(purrr)
library(tidyr)
```

```r
# compute alter betweenness (alter.betw)
alter.betw.data <- gr.list %>%
  map_dfr(~ {
    # Compute betweenness for each node
    node_betweenness <- betweenness(.x, weights = NA)
    # Create a data frame with node names and their betweenness scores
    data.frame(alter_id = names(node_betweenness), alter.betw = node_betweenness)
  },
  .id = "ego_id") %>%
  arrange(ego_id, alter_id)
head(alter.betw.data)
```

```
##     ego_id alter_id alter.betw
## 101      1      101         17
## 102      1      102          0
## 103      1      103          0
## 104      1      104          7
## 105      1      105          0
## 106      1      106          0
```

5. Model evaluation

5.1 Plotting the estimates from our models of interest

```
library(jtools)
library(broom.mixed)
```

```
plot_summs(m1, m2, m3)
```

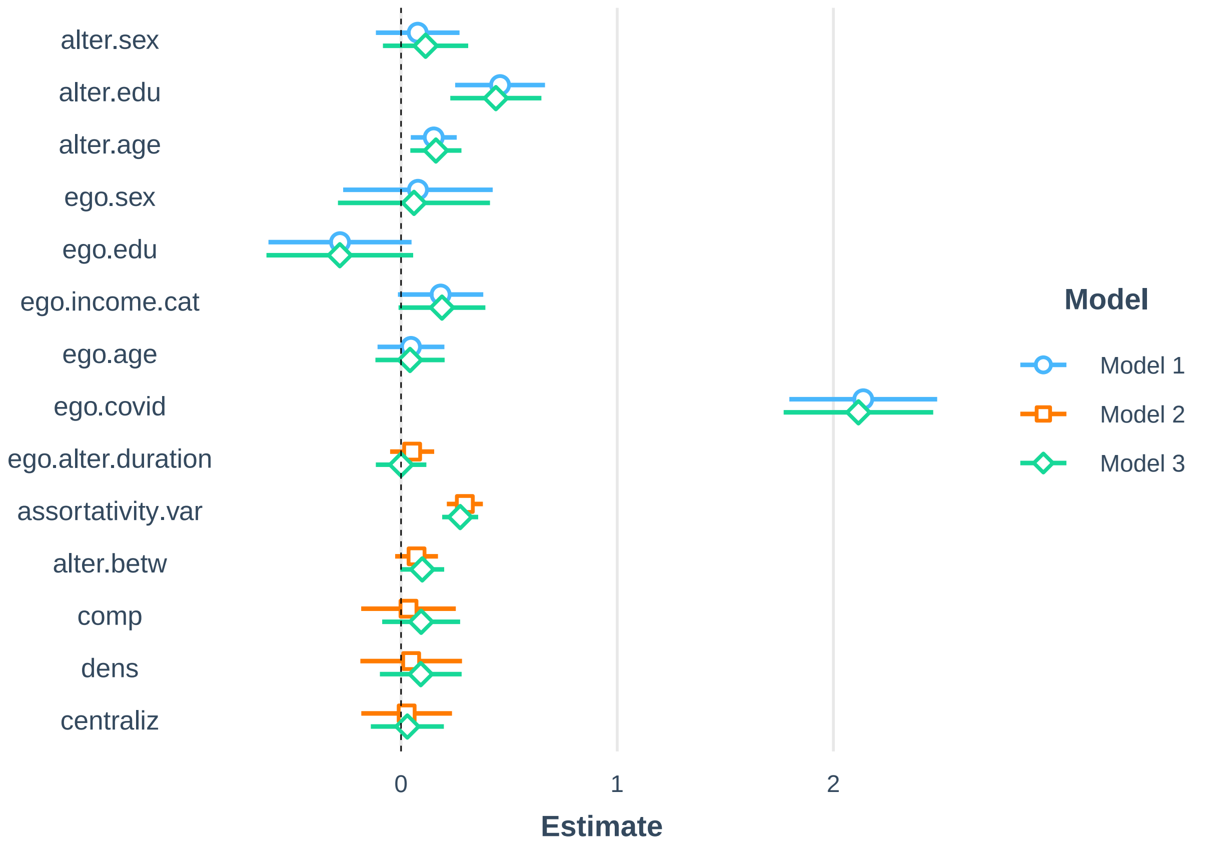

The plot of the estimates from the three alter multi-level logistic regression models visually allows the comparison of the estimated coefficients across models. This plot provides information to help understanding how the inclusion of different variables or the use of different model specifications can influence the estimates of the predictors.

In this plot, we illustrate the magnitude and direction of the effect of each predictor (i.e., dots far from zero indicate a larger effect sizes). Further, we stress that if the confidence intervals do not overlap with the zero line, the predictor is generally considered statistically significant. Also, this plot allows comparing across models and illustrates how the inclusion of different predictors affects the estimates. We showcase that the *assortativity.var* (the assortativity effect, the key variable in our study) is not affected by the inclusion of other types (blocks) of variables. Put it differently, this means consistency in the direction and magnitude across different models which suggests robustness.

5.2 Model interpretation

```
# 'attributes model' (predicting alters' opinions)
summ(m1
     , confint = TRUE
```

```r
      , scale = TRUE
      , exp = TRUE
      , digits = 2)
```

Observations	3588
Dependent variable	alter.covid
Type	Mixed effects generalized linear model
Family	binomial
Link	logit

AIC	3462.61
BIC	3524.46
Pseudo-R² (fixed effects)	0.16
Pseudo-R² (total)	0.38

Fixed Effects					
	exp(Est.)	2.5%	97.5%	z val.	p
(Intercept)	0.64	0.41	1.01	-1.91	0.06
alter.sex	1.08	0.89	1.31	0.78	0.44
alter.edu	1.58	1.28	1.95	4.32	0.00
alter.age	1.16	1.05	1.29	2.79	0.01
ego.sex	1.08	0.77	1.53	0.44	0.66
ego.edu	0.75	0.54	1.05	-1.67	0.09
ego.income.cat	1.16	0.99	1.37	1.82	0.07
ego.age	1.05	0.90	1.22	0.58	0.56
ego.covid	8.48	6.03	11.94	12.26	0.00

; Continuous predictors are mean-centered and scaled by 1 s.d. The outcome variable remains in its original units.

Random Effects		
Group	Parameter	Std. Dev.
ego_id	(Intercept)	1.07

Grouping Variables		
Group	# groups	ICC
ego_id	401	0.26

```r
# 'network model' (predicting alters' opinions)
summ(m2
      , confint = TRUE
      , scale = TRUE
      , exp = TRUE
      , digits = 2)
```

```r
# 'full model' (predicting alters' opinions)
summ(m3
```

Observations	3588
Dependent variable	alter.covid
Type	Mixed effects generalized linear model
Family	binomial
Link	logit

AIC	3576.01
BIC	3625.49
Pseudo-R^2 (fixed effects)	0.02
Pseudo-R^2 (total)	0.42

Fixed Effects					
	exp(Est.)	2.5%	97.5%	z val.	p
(Intercept)	4.36	3.61	5.27	15.34	0.00
ego.alter.duration	1.05	0.95	1.17	0.99	0.32
assortativity.var	1.34	1.24	1.46	6.94	0.00
alter.betw	1.07	0.97	1.19	1.42	0.16
comp	1.04	0.83	1.29	0.31	0.76
dens	1.05	0.83	1.33	0.39	0.70
centraliz	1.03	0.83	1.27	0.24	0.81

; Continuous predictors are mean-centered and scaled by 1 s.d. The outcome variable remains in its original units.

Random Effects		
Group	Parameter	Std. Dev.
ego_id	(Intercept)	1.52

Grouping Variables		
Group	# groups	ICC
ego_id	401	0.41

```
      , confint = TRUE
      , scale = TRUE
      , exp = TRUE
      , digits = 2)
```

Observations	3588
Dependent variable	alter.covid
Type	Mixed effects generalized linear model
Family	binomial
Link	logit

This output presents the results of our three regression models (`m1`, `m2`, `m3`) with a binomial distribution and logit link function, used to model the variable *alter.covid*, i.e., whether alter has a good & very good opinion (1) or bad & very bad opinion (0) about vaccination.

Model Information Observations: All models are based on 3588 observations and have the same dependent

AIC	3428.70
BIC	3527.67
Pseudo-R^2 (fixed effects)	0.18
Pseudo-R^2 (total)	0.39

Fixed Effects					
	exp(Est.)	2.5%	97.5%	z val.	p
(Intercept)	0.66	0.42	1.05	-1.74	0.08
alter.sex	1.12	0.92	1.36	1.13	0.26
alter.edu	1.55	1.26	1.91	4.08	0.00
alter.age	1.17	1.04	1.32	2.67	0.01
ego.sex	1.06	0.75	1.51	0.33	0.74
ego.edu	0.75	0.54	1.06	-1.64	0.10
ego.income.cat	1.17	0.99	1.38	1.85	0.06
ego.age	1.04	0.89	1.22	0.51	0.61
ego.covid	8.30	5.87	11.73	11.99	0.00
ego.alter.duration	1.00	0.89	1.12	0.00	1.00
assortativity.var	1.31	1.21	1.43	6.44	0.00
alter.betw	1.10	1.00	1.22	1.90	0.06
comp	1.10	0.92	1.31	1.01	0.31
dens	1.10	0.91	1.32	0.95	0.34
centraliz	1.03	0.87	1.22	0.34	0.74

; Continuous predictors are mean-centered and scaled by 1 s.d. The outcome variable remains in its original units.

Random Effects		
Group	Parameter	Std. Dev.
ego_id	(Intercept)	1.09

Grouping Variables		
Group	# groups	ICC
ego_id	401	0.26

variable (*alter.covid*). Model Type: These are mixed effects models, which account for both fixed and random effects.

Model Fit AIC and BIC: AIC (Akaike Information Criterion) and BIC (Bayesian Information Criterion) are measures of the model's goodness of fit with a penalty for the number of parameters. Lower values are better. Comparing these values across models helps in model selection. Models `m1` and `m3` have lower AIC and BIC values compared to `m2`, indicating potentially better fit. Pseudo R^2: This gives an idea of the variance explained by the model. Higher values indicate a better model fit. Total Pseudo R^2 is higher in `m2` and `m3` (0.42 and 0.39) compared to `m1` (0.38).

Fixed Effects exp(Est.): This column shows the exponentiated coefficients (odds ratios) for the predictors (see also the OR column in Table 3 of the manuscript). Values greater than 1 indicate a positive effect on the likelihood of 'alter.covid', and values less than 1 indicate a negative effect. 95% Confidence Interval (2.5%, 97.5%): This interval gives a range within which the true coefficient is expected to fall, with 95% confidence. If this interval does not include 1, the effect is considered statistically significant. z val.: The z-value is the ratio of the coefficient to its standard error. Larger absolute values indicate higher significance. p: p-values

test the hypothesis that each coefficient is different from zero. A low p-value (typically <0.05) indicates that the effect of the predictor is statistically significant.

Model m1: (Intercept): The exp(Est.) of 0.64 with a p-value of 0.06 suggests that the baseline odds (when all predictors are at their mean values) of the outcome 'alter.covid' being positive are lower than the reference group, but this effect is marginally significant.

alter.sex: An odds ratio of 1.08 ($p = 0.44$) implies a slight increase in the odds of 'alter.covid' being positive with a unit increase in 'alter.sex', but this effect is not statistically significant.

alter.edu: With an odds ratio of 1.58 ($p < 0.01$), this shows a significant positive effect on 'alter.covid', indicating that increases in 'alter.edu' are associated with higher odds of the outcome being positive.

alter.age: The odds ratio of 1.16 ($p = 0.01$) indicates a significant positive association with 'alter.covid'.

ego variables: Most ego-related variables (ego.sex, ego.edu, ego.income.cat, ego.age) show no significant effect on 'alter.covid', except for 'ego.covid', which has a very high odds ratio of 8.48 ($p < 0.01$), indicating a strong and significant association.

Model m2: This model introduces predictors like 'ego.alter.duration', 'assortativity.var', 'alter.betw', 'comp', 'dens', and 'centraliz'. Among these, 'assortativity.var' is significant with an odds ratio of 1.34 ($p < 0.01$), suggesting its strong positive effect on 'alter.covid'.

Model m3: Similar to `m1`, but includes additional predictors from `m2.` Notably, 'assortativity.var' remains significant. The 'ego.covid' effect remains strong and significant. The addition of new variables slightly changes the effect sizes and significance levels of some predictors compared to `m1.`

Random Effects Std. Dev.: This shows the standard deviation of the random effects (here, random intercepts for 'ego_id'). It indicates how much the intercept varies across groups (egos).

Model `m1`: The standard deviation of the random intercepts is 1.07. This indicates a moderate level of variability in the intercept across different egos. It suggests that the baseline log-odds of 'alter.covid' (the outcome) varies from one ego to another.

Model `m2`: Here, the standard deviation is higher at 1.52, indicating greater variability in the intercept across egos compared to `m1.` This suggests that the baseline tendency towards 'alter.covid' is more diverse across different egos in this model.

Model `m3`: The standard deviation is 1.09, which is similar to Model `m1`, indicating a comparable level of variability in the intercept across different egos. A model with a higher random effect variance (like in `m2`) might be capturing more of the unobserved heterogeneity across groups (egos) than a model with lower random effect variance.

Grouping Variables ICC (Intraclass Correlation Coefficient): It quantifies the proportion of total variance explained by the grouping structure. Higher values indicate that much of the variability is accounted for by the differences between groups. An ICC of 0.26 in `m1`, for instance, means that 26% of the total variability in 'alter.covid' can be explained by the differences between different egos. A higher ICC (as in `m2`, with an ICC of 0.41) suggests a stronger clustering effect or greater homogeneity within egos and more heterogeneity between different egos.

Pseudo-Rˆ2 Pseudo-Rˆ2 (Fixed Effects): This value represents the proportion of variance explained by the fixed effects alone in the model. It is a measure of how well the fixed effects (independent variables) explain the variability in the dependent variable, without accounting for the random effects. In our models, these values range from 0.16 in `m1` to 0.02 in `m2`, and 0.18 in `m3`. This suggests that the fixed effects in `m3` explain a slightly higher proportion of the variance in the outcome compared to `m1` and `m2.`

Pseudo-Rˆ2 (Total): This score accounts for both the fixed and random effects in the model. It provides an overall measure of model fit, including the variability explained by the grouping structure (random effects). In our models, the total Pseudo-Rˆ2 is highest in `m2` (0.42), followed by `m3` (0.39), and `m1` (0.38). This indicates that `m2`, despite having a lower fixed effects Pseudo-Rˆ2, explains more total variance when considering its random effects.

Comparative Measure: Pseudo-R^2 is more useful as a comparative measure rather than an absolute one. Higher values indicate a better fit, but the absolute values are less interpretable than in linear regression.

Model Selection: While these scores provide insight into model fit, they should be considered along with AIC/BIC, the model interpretability, and the research objectives underlining the model selection.

Overall, the Pseudo-R^2 scores in our models suggest varying degrees of model fit, with `m2` (the network model) performing slightly better in terms of total variance explained when both fixed and random effects are considered.

Additional Notes Continuous Predictors: These are mean-centered and scaled, making the coefficients more interpretable in terms of standard deviations. Model `m3` incorporates more variables compared to `m1` and `m2`, as indicated by the larger number of fixed effects.

5.3 Model comparison interpretation

```
anova(m0, m1, m2, m3,
      test = "Chisq")
```

```
## Data: na.omit(alter.df3)
## Models:
## m0: alter.covid ~ 1 + (1 | ego_id)
## m2: alter.covid ~ +ego.alter.duration + assortativity.var + alter.betw + comp + dens + centraliz + (
## m1: alter.covid ~ alter.sex + alter.edu + alter.age + ego.sex + ego.edu + ego.income.cat + ego.age +
## m3: alter.covid ~ alter.sex + alter.edu + alter.age + ego.sex + ego.edu + ego.income.cat + ego.age +
##    npar    AIC    BIC  logLik deviance   Chisq Df Pr(>Chisq)
## m0    2 3616.3 3628.7 -1806.1   3612.3
## m2    8 3576.0 3625.5 -1780.0   3560.0  52.281  6  1.637e-09 ***
## m1   10 3462.6 3524.5 -1721.3   3442.6 117.396  2  < 2.2e-16 ***
## m3   16 3428.7 3527.7 -1698.3   3396.7  45.911  6  3.084e-08 ***
## ---
## Signif. codes:  0 '***' 0.001 '**' 0.01 '*' 0.05 '.' 0.1 ' ' 1
```

Model Comparisons: Comparing `m0` and `m2`, the significant chi-square statistic (Chisq = 52.281, $p < 0.001$) indicates that the addition of ego-alter and network variables in `m2` significantly improves the model fit over the null model `m0`.

Comparing `m2` and `m1`, the substantial improvement in model fit is again significant (Chisq = 117.396, $p < 0.001$), suggesting that including demographic and individual-related variables in `m1` provides a better explanation of the outcome alter.covid.

Finally, comparing `m1` and `m3`, there is a significant improvement (Chisq = 45.911, $p < 0.001$), indicating that the most comprehensive model (`m3`), which combines the predictors of `m1` and `m2`, provides the best fit among the compared models.

Best Model: Based on this ANOVA comparison, `m3` is the best model in terms of explaining the variation in the dependent variable `alter.covid`, as it combines all sets of predictors and significantly improves upon the simpler models. The ANOVA results suggest that each additional set of variables included in the successive models significantly improves the model's ability to explain the variability in the dependent variable. The comprehensive model `m3`, which includes demographic, individual-related, ego-alter interaction, and network characteristics, along with random effects, provides the most substantial improvement in fit according to the data and the models considered.

5.4 Predictive performance

```
library(pROC)
```

```r
# model 1
data_m1 <- getData(m1)
predicted_probs_m1 <- predict(m1, type = "response")
roc_curve <- roc(data_m1$alter.covid, predicted_probs_m1)
```

```
## Setting levels: control = 0, case = 1

## Setting direction: controls < cases
```

```r
auc_value <- auc(roc_curve)
plot(roc_curve, main = sprintf("ROC Curve (AUC = %.2f)", auc_value))
```

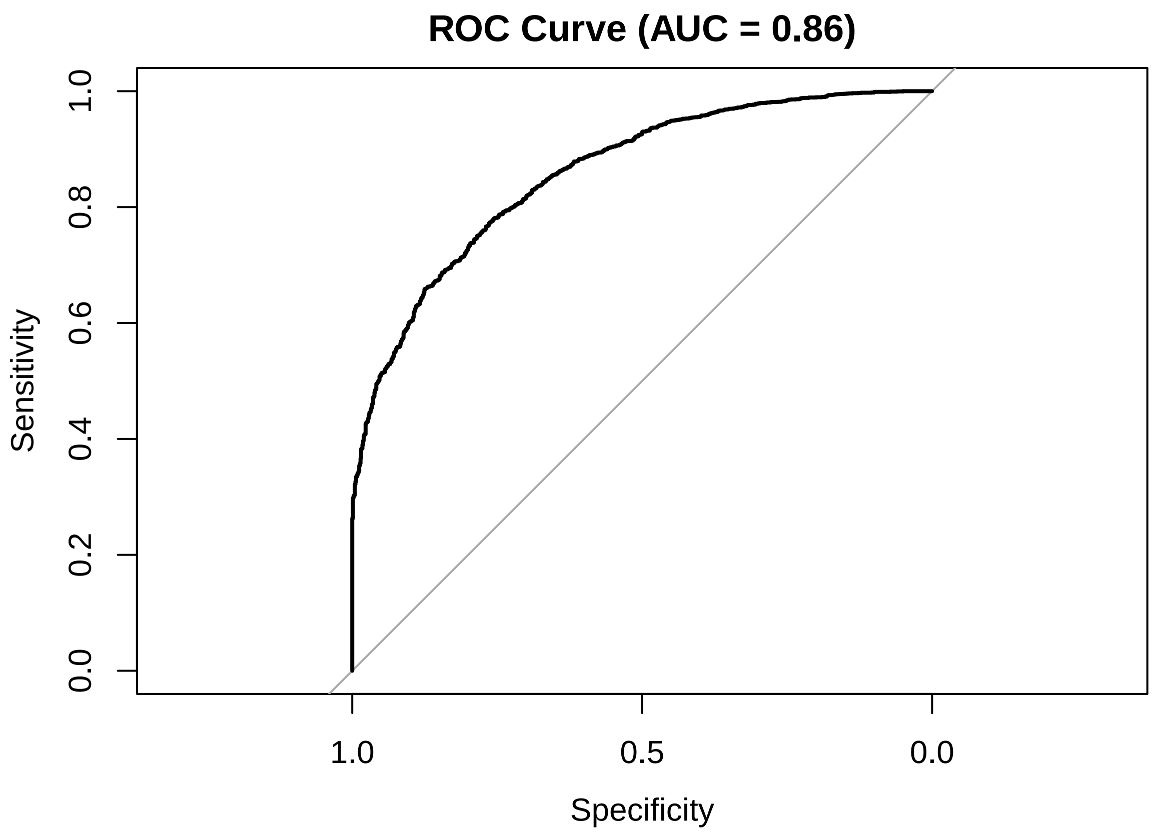


```r
# model 2
data_m2 <- getData(m2)
predicted_probs_m2 <- predict(m2, type = "response")
roc_curve <- roc(data_m2$alter.covid, predicted_probs_m2)
```

```
## Setting levels: control = 0, case = 1
## Setting direction: controls < cases
```

```r
auc_value <- auc(roc_curve)
plot(roc_curve, main = sprintf("ROC Curve (AUC = %.2f)", auc_value))
```

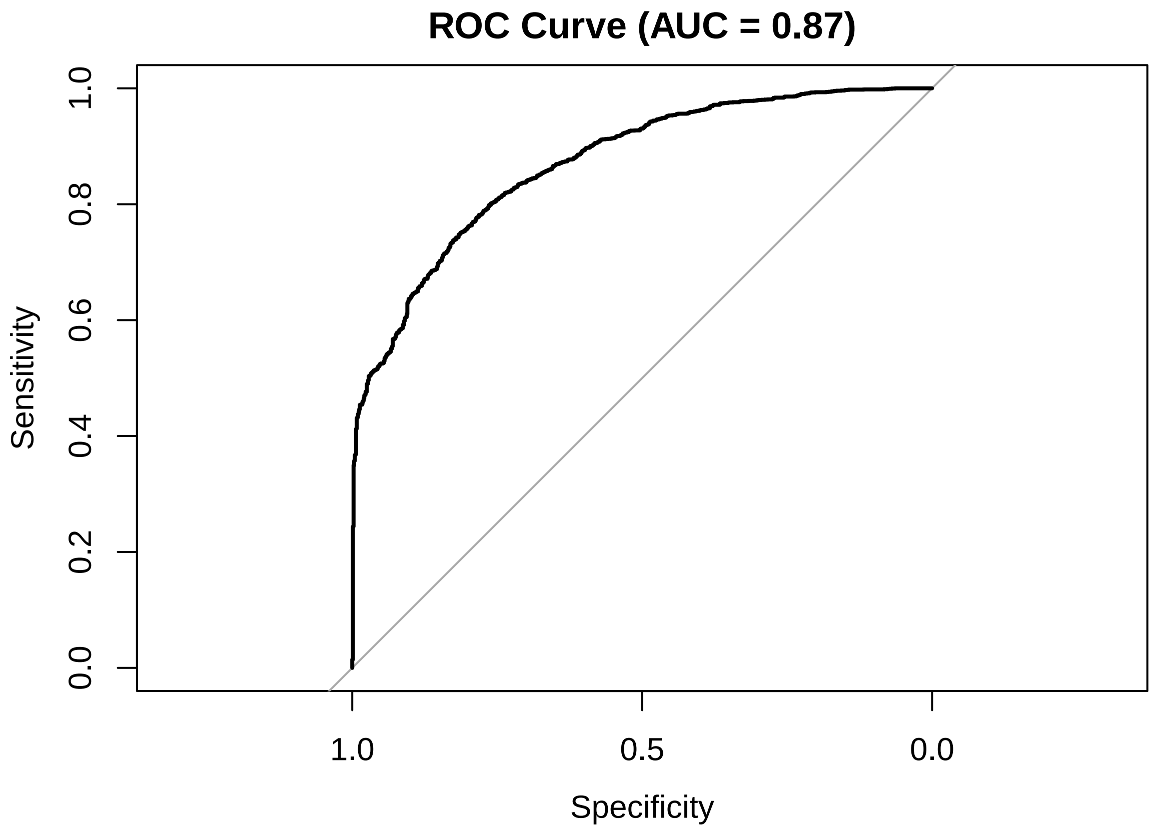


```
# model 3
data_m3 <- getData(m3)
predicted_probs_m3 <- predict(m3, type = "response")
roc_curve <- roc(data_m3$alter.covid, predicted_probs_m3)
```

```
## Setting levels: control = 0, case = 1
## Setting direction: controls < cases
```

```
auc_value <- auc(roc_curve)
plot(roc_curve, main = sprintf("ROC Curve (AUC = %.2f)", auc_value))
```

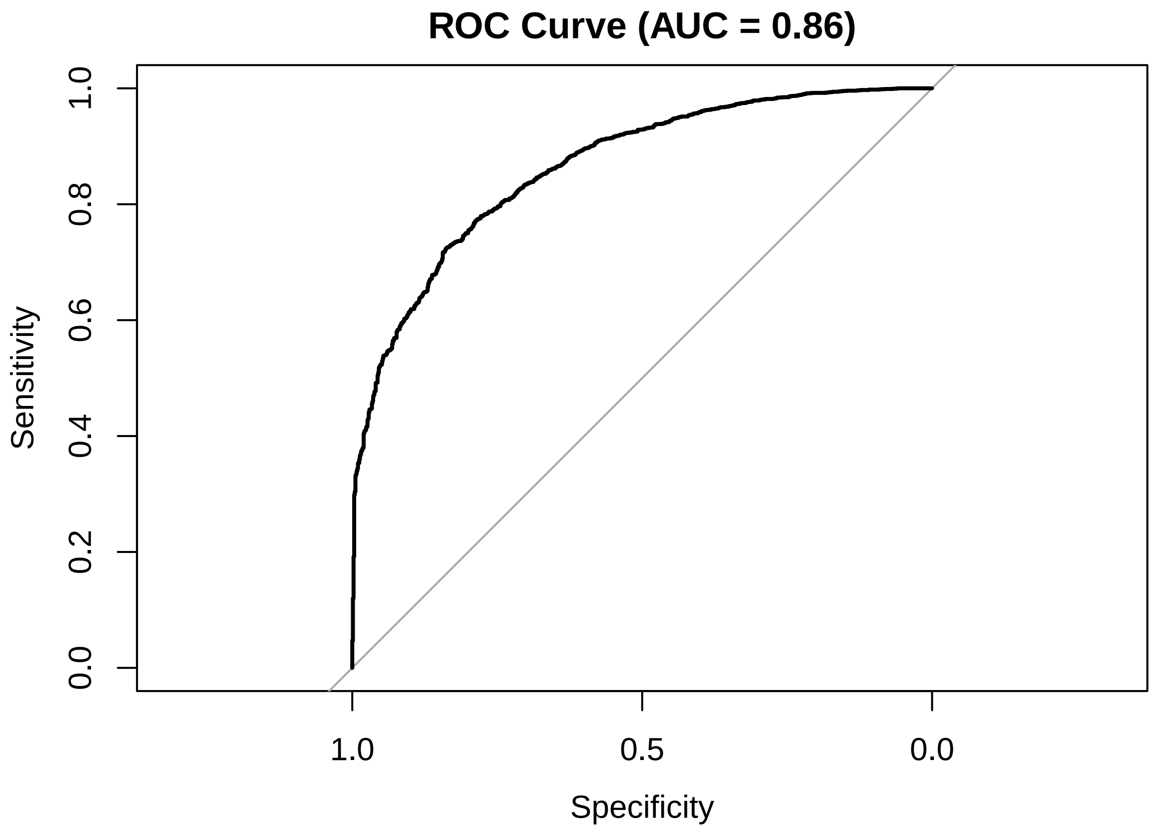

Our models have AUC (Area under the ROC curve) values of 0.86 (`m1`), 0.87 (`m2`) and 0.86 (`m3`) which can be considered to be very good. These indicate that our models have a high ability to discriminate between the binary outcomes (alter.covid). Basically, `m1`, `m2` and `m3` have similar predictive performances. Further, the models effectively account for the variability both at the individual level and across the ego-level.

5.5 Multi-collinearity check

```
library(car)
```

```
vif_values_m1 <- vif(m1)
vif_values_m2 <- vif(m2)
vif_values_m3 <- vif(m3)
vif_values_m1
```

```
##      alter.sex      alter.edu      alter.age        ego.sex        ego.edu
##       1.042675       1.154939       1.188755       1.136133       1.247218
## ego.income.cat        ego.age      ego.covid
##       1.270088       1.162738       1.013682
```

```
vif_values_m2
```

```
## ego.alter.duration  assortativity.var         alter.betw               comp
##           1.036950           1.000822           1.055118           1.691929
##               dens          centraliz
##           1.704894           1.323793
```

```
vif_values_m3
```

```
##           alter.sex           alter.edu           alter.age             ego.sex
##            1.060569            1.161965            1.436586            1.146727
##             ego.edu      ego.income.cat             ego.age           ego.covid
##            1.281867            1.284387            1.216965            1.015704
## ego.alter.duration   assortativity.var          alter.betw                comp
##            1.409964            1.003213            1.109773            1.726637
##                dens           centraliz
##            1.715429            1.338494
```

Model m1: All VIF values are close to 1 and well below 5, suggesting minimal multicollinearity among the predictors. This indicates that the variables (like alter.sex, alter.edu, alter.age, etc.) in model `m1` can be considered relatively independent in terms of their effect on the model.

Model m2: The VIF values are also low for most variables, indicating minimal multicollinearity. However, `comp` and `dens` have VIF values above 1.5, suggesting moderate correlation but still within generally acceptable limits.

Model m3: Similar to Model `m1`, most variables have low VIF values. But in this model, `alter.age`, `ego.alter.duration`, `comp`, and `dens` show higher VIF values, indicating moderate multicollinearity. While these values are slightly elevated, they are still below the threshold of 5, suggesting that multicollinearity may not be a significant concern.

Special note on the assortativity variable: The `assortativity.var` has a VIF score of 1.003 (`m3`) and 1.001 (`m2`) suggesting it is not correlated with the other predictors in the models.

Conclusion: The predictors in our models exhibit low to moderate multicollinearity based on the VIF values. There are no strong indicators of problematic multicollinearity in any of the models. Models `m1` and `m2` show very low multicollinearity overall, while Model `m3` has a few predictors with moderate multicollinearity. However, these are not at a level that would typically warrant major concern or require model modification. Given these VIF results, the stability and interpretability of the regression coefficients in our models are likely reliable.

5.6 Statistical tests for variable inclusion

We compute the `m2` and `m3` without the *assortativity* variable (our variable of interest). We will use these models (`m2_without`, `m3_without`) to perform statistical tests.

```
# estimate model 3 without the 'assortativity.var'
m3_without <- glmer(alter.covid ~
                      # alters' attributes (level 1 predictors)
                      alter.sex
                    + alter.edu
                    + alter.age
                    # egos' attributes (level 2 predictors)
                    + ego.sex
                    + ego.edu
                    + ego.income.cat
                    + ego.age
                    + ego.covid
                    # node-level properties
                    + ego.alter.duration
                    + alter.betw
                    # network-level properties
                    + comp
                    + dens
                    + centraliz
                    + (1 | ego_id) # the intercept can vary by ego (pers. netw.)
```

```
                    , family = "binomial"
                    , na.omit(alter.df3) # use only complete observations
                    , set.seed(1234)
                    , nAGQ = 100
                    , control=glmerControl(optimizer = "bobyqa")) # use R's optimization routine

# estimate model 2 without the 'assortativity.var'
m2_without <- glmer(alter.covid ~
                      # node-level properties
                      + ego.alter.duration
                    + alter.betw
                    # network-level properties
                    + comp
                    + dens
                    + centraliz
                    + (1 | ego_id) # the intercept can vary by ego (pers. netw.)
                    , family = "binomial"
                    , na.omit(alter.df3) # use only complete observations
                    , set.seed(1234)
                    , nAGQ = 100
                    , control=glmerControl(optimizer = "bobyqa")) # use R's optimization routine
```

We compare models *with* and *without* the `assortativity` variable to see if its inclusion significantly improves the model fit. We perform the *Likelihood Ratio Test*, comparing models with and without the assortativity variable (`assortativity.var`)

```
lr_test <- anova(m3, m3_without)
print(lr_test)
```

```
## Data: na.omit(alter.df3)
## Models:
## m3_without: alter.covid ~ alter.sex + alter.edu + alter.age + ego.sex + ego.edu + ego.income.cat + eg
## m3: alter.covid ~ alter.sex + alter.edu + alter.age + ego.sex + ego.edu + ego.income.cat + ego.age +
##            npar    AIC    BIC  logLik deviance  Chisq Df Pr(>Chisq)
## m3_without   15 3468.7 3561.4 -1719.3   3438.7
## m3           16 3428.7 3527.7 -1698.3   3396.7 41.963  1  9.303e-11 ***
## ---
## Signif. codes:  0 '***' 0.001 '**' 0.01 '*' 0.05 '.' 0.1 ' ' 1
```

Significant improvement with ***`assortativity.var`***: The chi-square statistic of 41.963 with a p-value of approximately 9.3e-11 (very close to 0) indicates that including `assortativity.var` in the model significantly improves the model fit. *Model Fit*: Both the AIC and BIC are lower in `m3` than in `m3_without`, further supporting that `m3` (with `assortativity.var`) is a better fit to the data.

Effect of ***`assortativity.var`***: The significant improvement in model fit implies that `assortativity.var` is an important predictor in understanding individuals' opinions about COVID-19 vaccination. It suggests that considering whether individuals surrounded by others with similar opinions impacts their own opinion adds significant explanatory power to the model.

Conclusion: In summary, the inclusion of `assortativity.var` in `m3` leads to a significant improvement in the model's ability to explain the variability in individuals' opinions about COVID-19 vaccination. This finding supports the theoretical relevance of `assortativity.var` and its practical importance in the analysis.

```
lr_test <- anova(m2, m2_without)
print(lr_test)
```

```
## Data: na.omit(alter.df3)
```

```
## Models:
## m2_without: alter.covid ~ +ego.alter.duration + alter.betw + comp + dens + centraliz + (1 | ego_id)
## m2: alter.covid ~ +ego.alter.duration + assortativity.var + alter.betw + comp + dens + centraliz + (1
##            npar    AIC    BIC  logLik deviance  Chisq Df Pr(>Chisq)
## m2_without    7 3623.1 3666.4 -1804.6   3609.1
## m2            8 3576.0 3625.5 -1780.0   3560.0 49.135  1  2.389e-12 ***
## ---
## Signif. codes:  0 '***' 0.001 '**' 0.01 '*' 0.05 '.' 0.1 ' ' 1
```

Model Improvement with `assortativity.var`: The significant chi-square statistic of 49.135 with a very low p-value (approximately 2.389e-12) indicates that including `assortativity.var` significantly improves the model fit.

Model Fit Indicators: Both the AIC and BIC are lower in `m2`, reinforcing that the model with `assortativity.var` provides a better fit for the data.

Importance of `assortativity.var`: The significant improvement in model fit suggests that `assortativity.var` is an important variable in explaining the variation in `alter.covid`. It implies that considering whether individuals are surrounded by others with similar opinions about COVID-19 vaccination is crucial for understanding their own opinions.

Conclusion: The inclusion of `assortativity.var` in `m2` results in a substantial improvement in the model's explanatory power. This finding underscores the relevance and importance of this variable in the context of studying individuals' opinions about COVID-19 vaccination.

Model comparisons (with and without the assortativity variable)

```
summ(m2_without)
```

Observations	3588
Dependent variable	alter.covid
Type	Mixed effects generalized linear model
Family	binomial
Link	logit

AIC	3623.14
BIC	3666.44
Pseudo-R^2 (fixed effects)	0.00
Pseudo-R^2 (total)	0.41

Fixed Effects				
	Est.	S.E.	z val.	p
(Intercept)	1.46	0.10	15.23	0.00
ego.alter.duration	0.05	0.05	0.94	0.35
alter.betw	0.06	0.05	1.25	0.21
comp	0.03	0.11	0.29	0.77
dens	0.04	0.12	0.37	0.71
centraliz	0.04	0.11	0.34	0.73

```
summ(m2)
```

```
summ(m3_without)
```

Random Effects		
Group	Parameter	Std. Dev.
ego_id	(Intercept)	1.52

Grouping Variables		
Group	# groups	ICC
ego_id	401	0.41

Observations	3588
Dependent variable	alter.covid
Type	Mixed effects generalized linear model
Family	binomial
Link	logit

AIC	3576.01
BIC	3625.49
Pseudo-R^2 (fixed effects)	0.02
Pseudo-R^2 (total)	0.42

Fixed Effects				
	Est.	S.E.	z val.	p
(Intercept)	1.47	0.10	15.34	0.00
ego.alter.duration	0.05	0.05	0.99	0.32
assortativity.var	0.30	0.04	6.94	0.00
alter.betw	0.07	0.05	1.42	0.16
comp	0.03	0.11	0.31	0.76
dens	0.05	0.12	0.39	0.70
centraliz	0.03	0.11	0.24	0.81

Random Effects		
Group	Parameter	Std. Dev.
ego_id	(Intercept)	1.52

Grouping Variables		
Group	# groups	ICC
ego_id	401	0.41

Observations	3588
Dependent variable	alter.covid
Type	Mixed effects generalized linear model
Family	binomial
Link	logit

AIC	3468.66
BIC	3561.44
Pseudo-R² (fixed effects)	0.16
Pseudo-R² (total)	0.38

Fixed Effects				
	Est.	S.E.	z val.	p
(Intercept)	-0.70	0.25	-2.81	0.01
alter.sex	0.10	0.10	0.97	0.33
alter.edu	0.46	0.11	4.36	0.00
alter.age	0.16	0.06	2.62	0.01
ego.sex	0.07	0.18	0.40	0.69
ego.edu	-0.30	0.17	-1.73	0.08
ego.income.cat	0.19	0.10	1.86	0.06
ego.age	0.04	0.08	0.53	0.60
ego.covid	2.14	0.17	12.28	0.00
ego.alter.duration	0.00	0.06	0.04	0.97
alter.betw	0.09	0.05	1.73	0.08
comp	0.09	0.09	0.99	0.32
dens	0.09	0.10	0.92	0.36
centraliz	0.04	0.09	0.46	0.64

Random Effects		
Group	Parameter	Std. Dev.
ego_id	(Intercept)	1.07

Grouping Variables		
Group	# groups	ICC
ego_id	401	0.26

```
summ(m3)
```

Observations	3588
Dependent variable	alter.covid
Type	Mixed effects generalized linear model
Family	binomial
Link	logit

AIC	3428.70
BIC	3527.67
Pseudo-R² (fixed effects)	0.18
Pseudo-R² (total)	0.39

Impact of `assortativity.var`

Model m2_without vs. m2 (Inclusion of `assortativity.var`):

Fixed Effects				
	Est.	S.E.	z val.	p
(Intercept)	-0.66	0.25	-2.64	0.01
alter.sex	0.11	0.10	1.13	0.26
alter.edu	0.44	0.11	4.08	0.00
alter.age	0.16	0.06	2.67	0.01
ego.sex	0.06	0.18	0.33	0.74
ego.edu	-0.28	0.17	-1.64	0.10
ego.income.cat	0.19	0.10	1.85	0.06
ego.age	0.04	0.08	0.51	0.61
ego.covid	2.12	0.18	11.99	0.00
ego.alter.duration	0.00	0.06	0.00	1.00
assortativity.var	0.27	0.04	6.44	0.00
alter.betw	0.10	0.05	1.90	0.06
comp	0.09	0.09	1.01	0.31
dens	0.09	0.10	0.95	0.34
centraliz	0.03	0.09	0.34	0.74

Random Effects		
Group	Parameter	Std. Dev.
ego_id	(Intercept)	1.09

Grouping Variables		
Group	# groups	ICC
ego_id	401	0.26

- *AIC/BIC Reduction*: Including `assortativity.var` reduced AIC from 3623.14 to 3576.01 and BIC from 3666.44 to 3625.49, indicating a better model fit.
- *Pseudo R^2 Improvement*: The total Pseudo R^2 increased marginally from 0.41 to 0.42, suggesting a slightly better explanation of the variance.
- *Significant Predictor*: `assortativity.var` has a significant effect (Est. = 0.30, z = 6.94, p < 0.01), highlighting its importance in predicting `alter.covid`.

Model m3_without vs. m3 (Inclusion of assortativity.var):

- *AIC/BIC Reduction*: The inclusion of `assortativity.var` led to a decrease in AIC from 3468.66 to 3428.70 and BIC from 3561.44 to 3527.67, suggesting a more efficient model.
- *Pseudo R^2 Improvement*: The total Pseudo R^2 increased from 0.38 to 0.39, indicating a marginally better model performance.
- *Significant Predictor*: Again, `assortativity.var` is significant (Est. = 0.27, z = 6.44, p < 0.01), affirming its role in the model.

General interpretation:

- *Model Efficiency*: The inclusion of `assortativity.var` leads to more efficient models (as indicated by lower AIC and BIC) and a slight improvement in explanatory power.
- *Significance of* `assortativity.var`: This variable consistently shows a strong, significant association with the outcome `alter.covid` across models where it is included, justifying its inclusion.

- *Comparison with other predictors*: When `assortativity.var` is included, it stands out as one of the more influential predictors, considering its significant z-values and p-values, compared to many other predictors that do not show significant effects.

5.7 Wald test

In this sub-section, we evaluate the significance of the assortativity effect (`assortativity.var`) in our models, using the `Wald test`.

```
library(aod)
```

```
# Wald test for 'assortativity.var'
set.seed(1234)
wald.test(b = fixef(m2), Sigma = vcov(m2), Terms = c(3))
```

```
## Wald test:
## ----------
##
## Chi-squared test:
## X2 = 48.2, df = 1, P(> X2) = 3.9e-12
```

```
set.seed(1234)
wald.test(b = fixef(m3), Sigma = vcov(m3), Terms = c(11))
```

```
## Wald test:
## ----------
##
## Chi-squared test:
## X2 = 41.5, df = 1, P(> X2) = 1.2e-10
```

Statistical significance: For both models `m2` and `m3`, the Wald tests indicate that 'assortativity.var' is statistically significant. The p-values are well below the conventional threshold of 0.05, implying strong evidence against the null hypothesis (that `assortativity.var` has no effect).

Effect of `assortativity.var`: The significance of `assortativity.var` in both models suggests that this variable has an important positive relationship with the outcome variable in each model.

Model comparison: The fact that `assortativity.var` is significant in both models strengthens the argument for its importance in explaining the dependent variable in our study. This consistency across the two models adds robustness to our findings.
